# Cenozoic origins of the genus *Calliarcys* (Insecta, Ephemeroptera) revealed by Micro-CT, with DNA barcode gap analysis of Leptophlebiinae and Habrophlebiinae

**DOI:** 10.1038/s41598-022-18234-4

**Published:** 2022-09-08

**Authors:** Roman J. Godunko, Javier Alba-Tercedor, Michal Grabowski, Tomasz Rewicz, Arnold H. Staniczek

**Affiliations:** 1 Biology Centre of the Czech Academy of Sciences, Institute of Entomology, Branišovská 31, 37005 České Budějovice, Czech Republic; 2 Department of Invertebrate Zoology and Hydrobiology, University of Łódź, Banacha 12/16, 90237 Łódź, Poland; 3 State Museum of Natural History, NAS Ukraine, Teatralna 18, Lviv, 79008 Ukraine; 4 Department of Zoology, Faculty of Sciences, University of Granada, Avenida de Fuente Nueva s/n, 18071 Granada, Spain; 5 Department of Entomology, State Museum of Natural History Stuttgart, Rosenstein 1, 70191 Stuttgart, Germany

**Keywords:** Molecular biology, Zoology, Entomology

## Abstract

Mayflies (Ephemeroptera) are among the oldest pterygote insects, with the earliest fossils dating back to the Late Carboniferous. Within mayflies, Leptophlebiidae are a highly diverse and widespread group, with approximately 140 genera and 640 species. Whereas taxonomy, systematics, and phylogeny of extant Leptophlebiidae are in the focus of extensive studies, little is known about leptophlebiid fossil taxa. Because fossil remains of Ephemeroptera in sedimentary rocks are relatively rare, inclusions of mayflies in amber are a unique source of information on their evolution and diversity in the past. Leptophlebiidae found in Cenozoic resins mostly belong to the subfamilies Leptophlebiinae (in Eocene Baltic amber) and Atalophlebiinae (in Miocene Dominican and Mexican ambers). In the present contribution, we confirm the first finding of the genus *Calliarcys* from Eocene Baltic amber by using Micro-CT, which allowed confirming its generic placement by visualizing diagnostic key characters otherwise hidden by a cloud of turbidity. Additionally, we present first molecular data on the extant species *Calliarcys humilis* Eaton, 1881 from the Iberian Peninsula and the barcode gap analysis for Leptophlebiinae and Habrophlebiinae.

The genus *Calliarcys* Eaton, 1881 was established for the species *C. humilis* Eaton, 1881, which was described based on male and female adults from Spain^1^. A detailed overview on taxonomy, distribution, and current knowledge of *C. humilis* was given by Godunko et al.^2^. Originally thought to be an isolated West Palearctic species, more than 130 years later a second extant species, *Calliarcys van* Godunko and Bauernfeind, 2015, was described from two quite isolated localities in Turkey, namely in the Eastern Mediterranean (Izmir Province; W Turkey) and in East Anatolia (Bitlis Province). Its description led to a revision of diagnostic characters for *Calliarcys* and to the conclusion that *Calliarcys* has rather circum-Mediterranean origins^2^. However, since the original description of the genus, its phylogenetic position within Leptophlebiidae remained enigmatic and controversial.

The higher classification of Leptophlebiidae proposed by Peters^3^ recognized only two subfamilies, Leptophlebiinae and Atalophlebiinae. Discussing the systematic position of some extinct Mesozoic and Cenozoic taxa, Kluge^4^ only recognized Leptophlebiinae as monophyletic group and considered Atalophlebiinae as paraphyletic. Later, Kluge suggested a split of Leptophlebiinae s.l. into two subfamilies, Habrophlebiinae and Leptophlebiinae s.str.^4^. Peters and Gilles^5^ introduced the presence of squared ommatidia of the upper portion of the male compound eyes as convincing autapomorphic character supporting the monophyly of Atalophlebiinae. Peters^6^ added the fused styliger plate as additional apomorphic character shared by the Atalophlebiinae lineage (including the *Terpides* lineage + *Castanophlebia*). Kluge^7^ in a revised phylogenetic classification of Atalophlebiinae s.l. split off three new subfamilies: Terpidinae and Castanophlebiinae were established for 19 species previously assigned to Atalophlebiinae. For *Calliarcys,* the monotypic subfamily Calliarcyinae was proposed^7^.

Based on a set of 20 morphological characters of nymphs and adults, Leptophlebiinae were confirmed as sister group to the remaining Leptophlebiidae in the first strict cladistic analysis. Calliarcyinae were revealed as sister group of the clade Habrophlebiinae + Atalophlebiinae s.l. (= (Atalophlebiinae s.str. + Castanophlebiinae) + Terpidinae)^2^. However, this concept proposed by Kluge^7^ and later corroborated by Godunko et al.^2^ remained controversial: Bauernfeind and Soldán^8^ rather followed Kluge^4^, placing *Calliarcys* provisionally within Leptophlebiinae, at the same time pointing to some characters of *Calliarcys* common with Habrophlebiinae.

All these higher phylogenies of Leptophlebiidae discussed above were solely based on morphological evidence. In a first molecular analysis using two nuclear markers, O’Donnell and Jockusch^9^ did not even recover the monophyly of Leptophlebiidae (see also^2^). Atalophlebiinae were also not recovered as monophyletic, only each Leptophlebiinae and Habrophlebiinae were reported as monophyletic^9^. Contrary to these findings, the monophyly of Leptophlebiidae was later confirmed by Ogden et al.^10^ based on a large, combined set of morphological and molecular characters. Analysing 153 leptophlebiid taxa within 53 genera using the two molecular markers COI + 28S, Monjardim et al.^11^ yet proposed a new higher phylogeny of this family. Basically, there were three monophyletic branches recovered: Atalophlebiinae s.l. was confirmed, though with uncertain position of Castanophlebiinae, and the third branch was represented by Habrophlebiinae + Leptophlebiinae. Unfortunately, *Calliarcys* was not included in this molecular study. More recently, Gatti et al.^12^ also used a molecular dataset to investigate historical events linked to the vicariance and dispersal of Atalophlebiinae in the second phase of the Gondwanan breakup. Fossils were used to calibrate the time of origin of phylogenetic nodes, but until now no fossil representatives of *Calliarcys* have been available.

In this contribution, we aim to broaden the knowledge of this enigmatic genus, describing the first fossil representative *Calliarcys antiquus* **sp. nov.**, from the Eocene Baltic Amber. Its description is also the first establishment of a mayfly species by Micro-CT investigation. This method has by now frequently proven its potential to address the problems linked to taxonomy and systematics of fossils embedded in Mesozoic and Cenozoic resins (see, e.g.^13–15^). Its application in mayfly systematics however has been limited so far, with the visualisation of genitalia in the redescription of two extant mayfly species^16^ and the report of a potential case of phoresis in a fossil mayfly^17^. With the Micro-CT investigation of *Calliarcys antiquus* **sp. nov.**, we intend to reconstruct and describe its genitalia to confirm its generic placement, and to specify diagnostic generic characters based on both extant and extinct species. In addition, we provide the first DNA barcode data for the extant *Calliarcys humilis* as well as provide a gap analysis of the DNA barcode data for all the extant Leptophlebiinae and Habrophlebiinae.

## Results

**Systematic Paleontology**

Subphylum Hexapoda Latreille, 1825

Class Insecta Linnaeus, 1758

Order Ephemeroptera Hyatt & Arms, 1890

Family Leptophlebiidae Banks, 1900

Genus ***Calliarcys*** Eaton, 1881

*Calliarcys* Eaton, 1881: *Entomol. Mon. Mag*. 18: 21.

**Type species:** *Calliarcys humilis* Eaton, 1881; ibid.: 21 [*original designation*].

**Included species:** *Calliarcys humilis* Eaton, 1881 [extant; Portugal and Spain]; *Calliarcys van* Godunko & Bauernfeind, 2015 [extant; Turkey]; *Calliarcys antiquus* **sp. nov.** [extinct; Eocene Baltic amber].

**Revised diagnosis of adults** (modified from^2,8^). (i) Two pairs of connected intercalary veins of different length in cubital field of forewing; (ii) costal process of hind wing situated nearly at half length, well developed, slightly asymmetrical or symmetrical, bluntly pointed or rounded apically; (iii) penis lobes straight, simple, and tubular; (iv) tip of penis lobes slightly bent inwards, without blade-like process apically; (v) posterior margin of ventral forceps base deeply concave medially, with V-shaped medial incision and two long, submedian projections rounded apically and directed caudally; (vi) segment I of forceps longest, at least 3 × longer than segment II, without inner process or appendages; (vii) terminal and subterminal segments of forceps shorter than first segment; terminal segment shortest.

*Calliarcys antiquus* **sp. nov.** Godunko, Alba-Tercedor & Staniczek

urn:lsid:zoobank.org:act:697F8977-69BB-45CE-A947-F7772AD70F3D.

**Figure 1:**
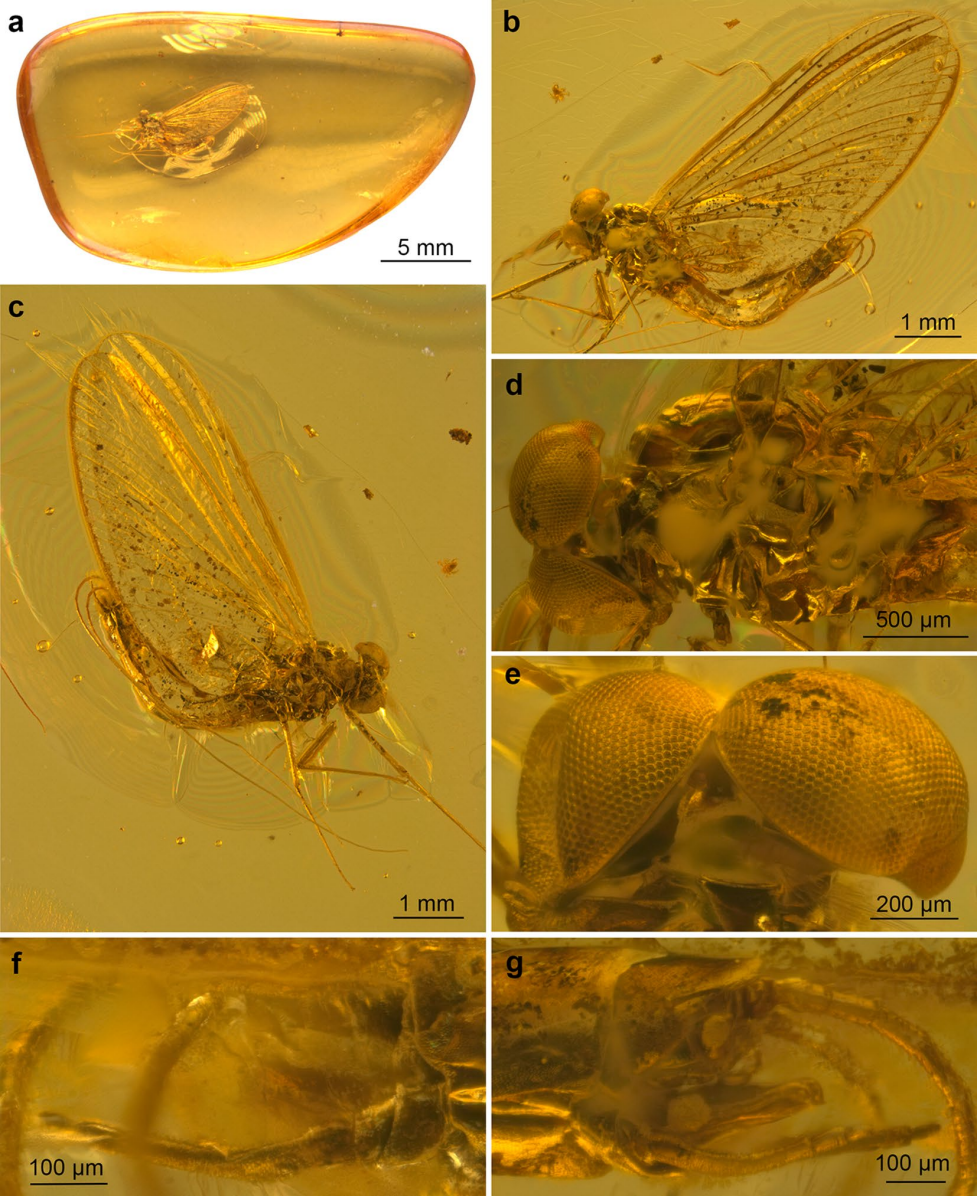
*Calliarcys antiquus* **sp. nov.,** holotype, male imago; Eocene Baltic amber; BB 2515 [SMNS coll.]; (**a**) entire piece of amber with embedded holotype in lateral view; (**b**) enlarged total lateral view from left side; (**c**) total lateral view from right side; (**d**) head and thorax in dorsolateral view from left side; (**e**) details of compound eyes in dorsal view; (**f**) genitalia in right lateral view; (**g**) genitalia in left lateral view.

**Figure 2:**
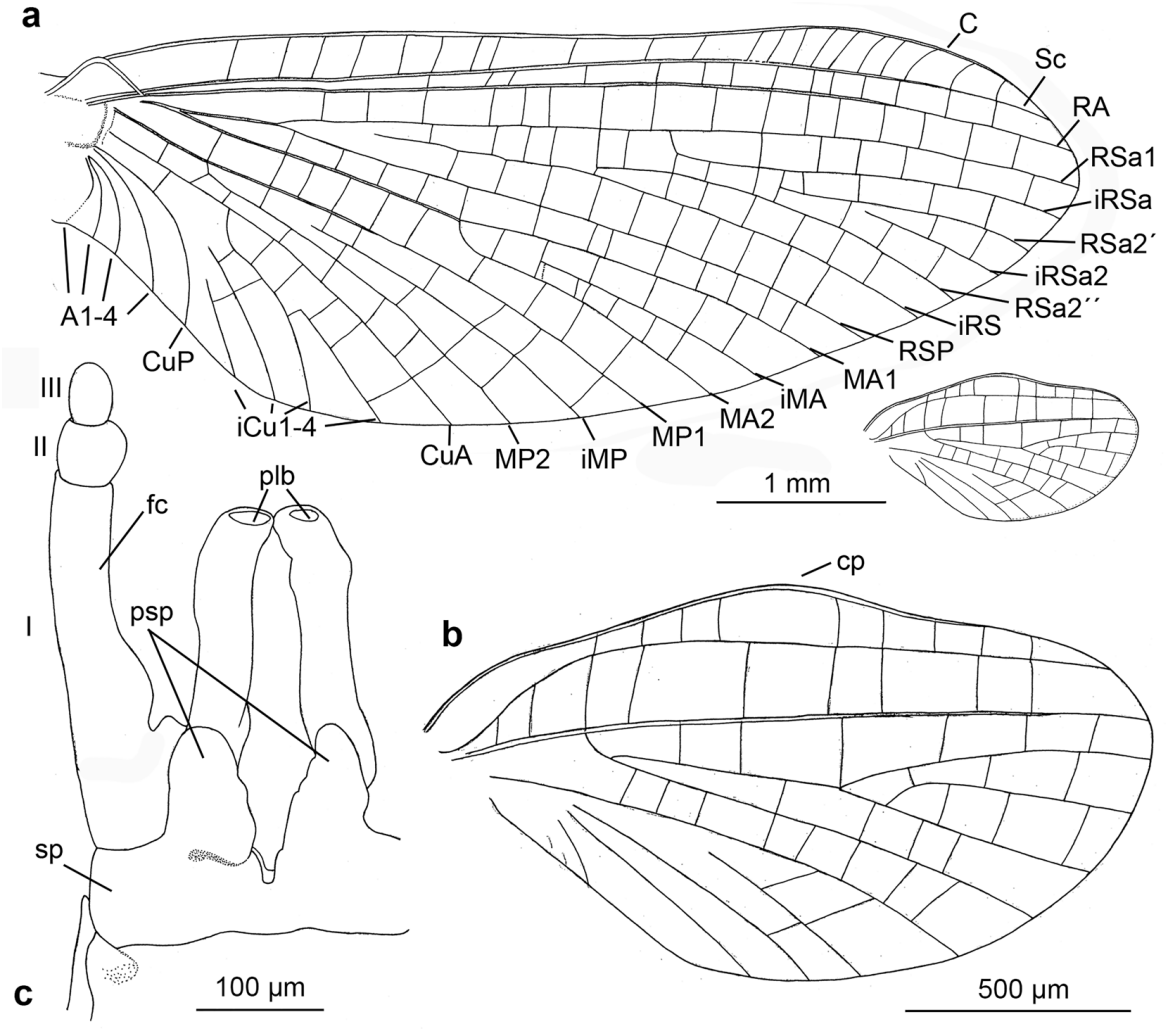
*Calliarcys antiquus* **sp. nov.,** holotype, male imago; Eocene Baltic amber; BB 2515 [SMNS coll.]; (**a**) fore- and hind wings (wing proportions are preserved), (**b**) enlarged hind wing, (**c**) genitalia in ventral view; fc—forceps; I-III—forceps segments; plb—penis lobe; psp—projections of styliger plate; sp—styliger plate; cp—costal process. Nomenclature of wing veins used throughout the text is based on ^37^.

**Figure 3:**
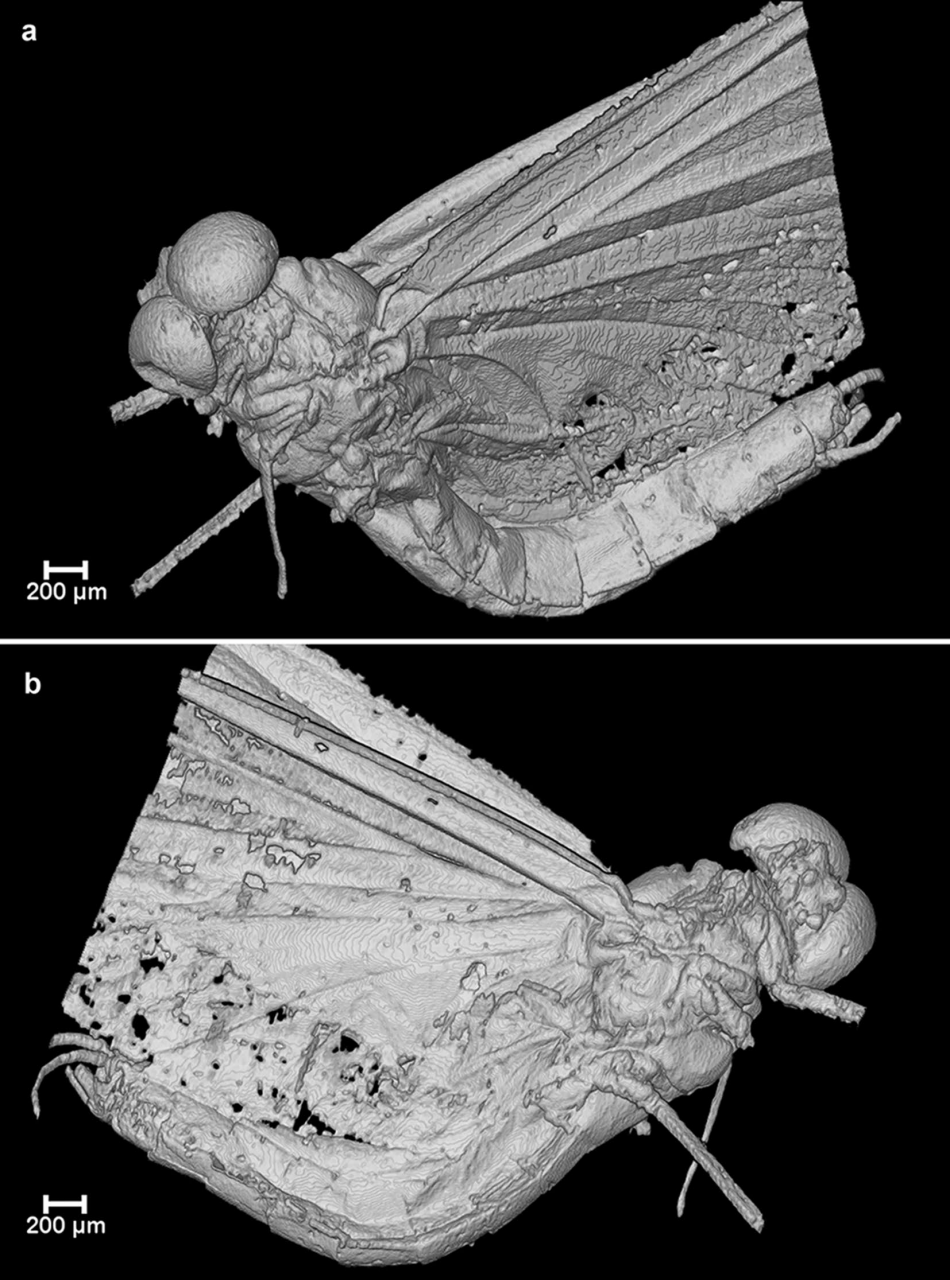
Micro-CT volume-rendered images of *Calliarcys antiquus* **sp. nov.**, holotype, male imago; Eocene Baltic amber; BB 2515 [SMNS coll.]; (**a**) body in left lateral view; (**b**) body in right lateral view.

**Figure 4:**
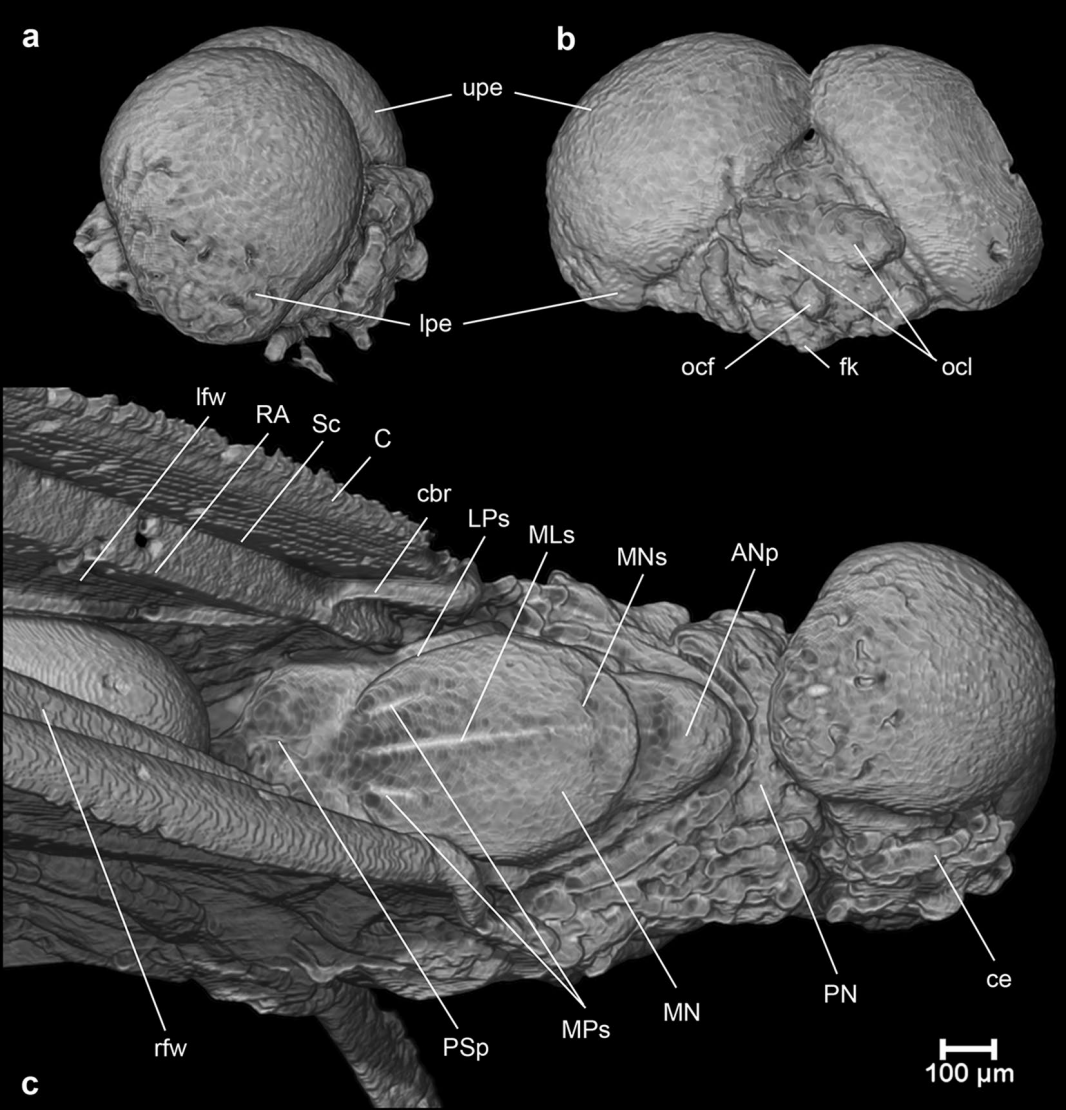
Micro-CT volume-rendered images of *Calliarcys antiquus* **sp. nov.**, holotype, male imago; Eocene Baltic amber; BB 2515 [SMNS coll.]; (**a**) head in right lateral view; (**b**) head in frontal view; (**c**) head and thorax in dorsal view. Abbreviations: ce—head; fk—facial keel; foc—frontal ocellus; loc—lateral ocelli; lp—lower portion of eye; up—upper portion of eye; C—costa; Sc—subcosta; RA—radius anterior; cbr—costal brace; lfw—left forewing; rfw—right forewing; ANp—anteronotal protuberance; LPs—lateroparapsidal suture; MN—mesonotum; MNs—mesonotal suture; MLs—median longitudinal suture; MPs—medioparapsidal suture; PN—pronotum; PSp—posterior scutal protuberance. Nomenclature of thoracic structures used throughout the text is based on ^37^.

**Figure 5:**
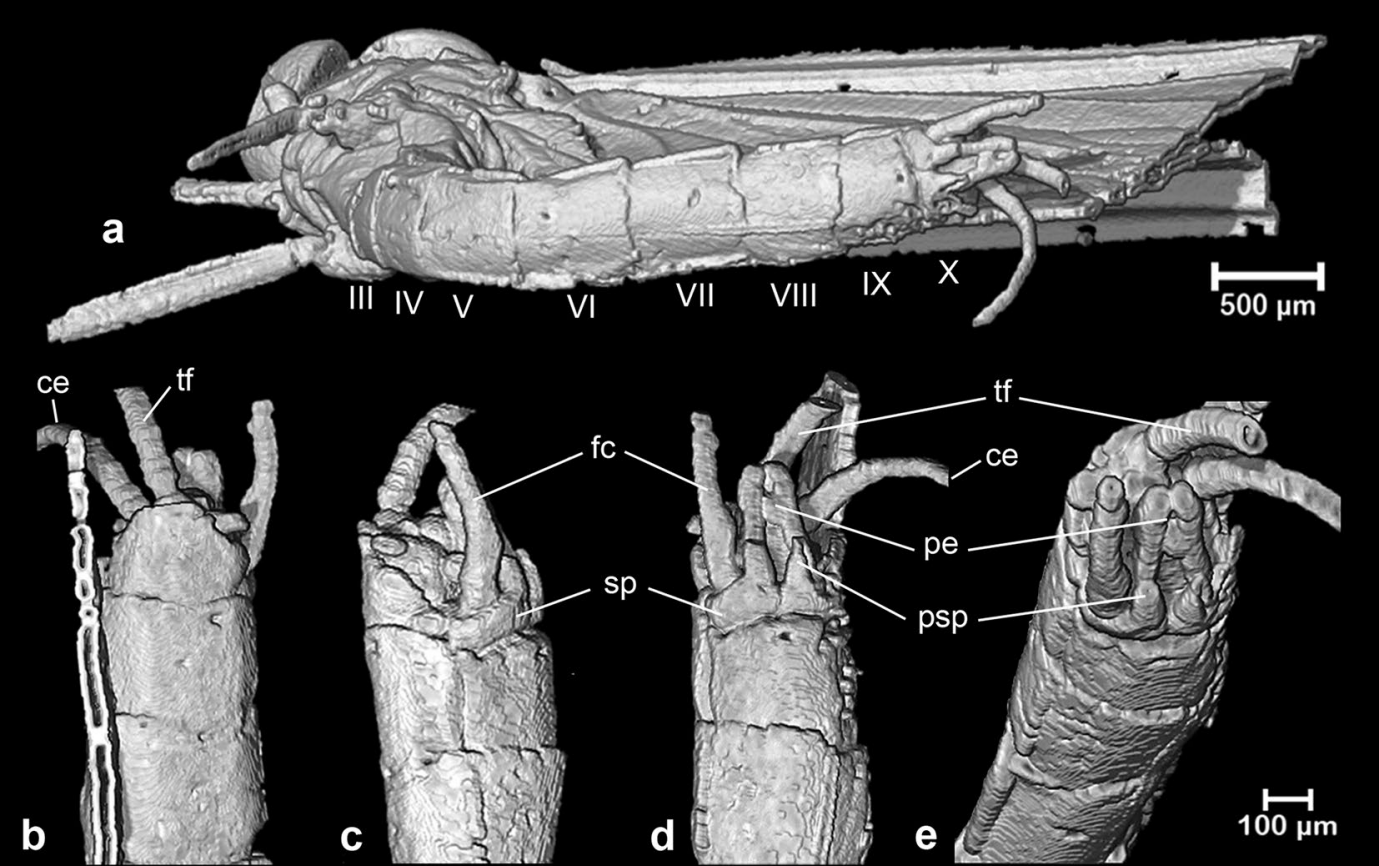
Micro-CT volume-rendered images of *Calliarcys antiquus* **sp. nov.**, holotype, male imago; Eocene Baltic amber; BB 2515 [SMNS coll.]; (**a**) body in ventral view; (**b**) tip of abdomen in dorsal view; (**c**) tip of abdomen with genitalia in left lateral view; (**d**) tip of abdomen with genitalia in ventral view; (**e**) tip of abdomen with genitalia in ventrocaudal view; ce—cercus; tf—paracercus; III-X—abdominal segments; fc—forceps; sp—styliger plate; pe—penes; psp—projection of styliger plate.

Figures 1–5; Tables 1 and 2.

### Type material

***Holotype.*** Male imago in Baltic Amber, Middle Eocene (35–47 million years), SMNS collection, inventory number BB 2515.

### Derivation of name

The genus name “*Calliarcys*” is a compound noun of masculine gender derived from the Greek κάλλη (kalli, for “beauty”) and ἄρκυς (arkys, latinized arcys, for net). The species epithet “*antiquus*” (masculine adjective, Latin for “ancient”) refers to the ancient origin of the fossil preserved in Eocene resin.

### Diagnosis

***Male imago.*** The extinct species *Calliarcys antiquus* **sp. nov.** differs from the two extant representatives of the genus by the following combination of characters: (i) upper portion of *c*ompound eyes large, medially contiguous at full length; (ii) hind wings with well-developed cross venation; (iii) costal process of hind wings widely rounded, strongly symmetrical, situated centrally and slightly rising above inner margin of wing; (iv) penis lobes simple, not obliquely truncate apically, without processes or appendices; (v) penis lobes seem to be separated basally, with tips slightly bent inwards and touching apically.

### Description

***Male imago*** (Figs. 1–5 and Supplementary Videos S2 and S3). Well preserved and almost complete specimen in clear, translucent amber, well visible in the lateral aspect; right foreleg and left hind leg missing (Fig. 1a–c). Lateral sides of thorax and tip of abdomen covered by a cloud of turbidity (so-called “Verlumung”, see Fig. 1d). Piece of amber with numerous cracks, organic debris, and multiple resin layers, thus details of male genitalia are hardly visible from dorsal and ventral sides in optical view (Fig. 1f–g). Additionally, the ventral side of styliger plate is partly covered by the preserved cercus and medial terminal filament, so shape of styliger plate and details of penis lobes are hardly distinguishable. Hind wings present, but details of venation are poorly discernible. All these hidden details in regular view however could be made visible by Micro-CT reconstruction (see below).

*Colours*. Preserved colour of specimen is yellowish to brown, with blackish-brown spots laterally on thorax and abdomen. Eyes pale, dirty yellow to light brown. Facial keel intensely brown, darker than eyes. Mesonotum darkest, dark brown, with blackish maculae laterally; wing surface covered by artificial, small brownish and blackish maculae [*as result of fossilisation*]; narrow brownish strip along outer margins of forewings. Legs unicoloured brown. Abdomen with translucent terga III–VII; abdominal sterna and three last abdominal segments intensively brown; traces of dark brown maculation along lateral margins of abdominal segments (Fig. 1b–c).

*Measurements*. Body length 5.40 mm; forewing length 5.68 / 5.72 mm; length of right cercus 6.10 mm; terminal filament length 7.70 mm. Maximum forewing width 0.38 × maximum length; hind wing 0.27 × of forewing length. For other measurements, see Table 1.

*Head* (Figs. 1d,e, 3a,b and 4a–c)*.* General colour yellowish-brown to dark brown. Relatively small, brown facial keel. Antennae brown*.* Ocelli indistinct, relatively small, without conspicuous coloration. Upper portion of compound eyes well developed, large, widely rounded, contiguous medially; facets of compound eyes hexagonal. Border between upper and lower portions of compound eyes well distinguishable.

*Thorax* (Figs. 1b–d, 3 and 4a). Thoracic terga darker than pleurae, brown to dark brown; pleurae light brown. Thoracic sterna paler than terga, brown. Mesonotal suture transverse centrally, curved posteriorly, stretching backward distally; medioparapsidal sutures straight anteriorly, slightly curved inward posteriorly; lateroparapsidal suture distinct, relatively short, smoothly curved inward posteriorly (Figs. 3 and 4a). Scutellum not modified. Natural colouration of pigmented area of mesonotum not preserved (Fig. 1d). Basisternum of mesonotum slightly elongated; furcasternal protuberances clearly separated; mesosternum with intensively brown basisternum and furcasternal protuberances (Figs. 1b–d and 3b).

*Wings* (Figs. 2a–b, 3a,b and 4c). *Forewings* hyaline, translucent, not frosted. Longitudinal venation distally light brown to brown proximally; cross venation well developed, whitish yellow to yellow, poorly recognizable distally. No short free intercalary veins between Sc and CuA (Figs. 1b–c and 2a).

Pterostigma is translucent, mainly with 6–7 forked veins and only 2–3 simple veins. C and Sc dark brown and well visible throughout their length. RS forked near base, after 0.15 of its length; cross venation of RS well-developed. iRS well-developed, connected to RSp by 9–10 cross veins, slightly approximated to RSa1. Asymmetric MA fork, forked after 0.44 to 0.46 of its length; MA1 and MA2 connected to iMA by 5 to 7 cross veins. MP slightly asymmetrical, forked after 0.18 of its length; MP1 and MP2 are basally connected by a single cross vein; iMP relatively long, connected to MP1 and MP2 by 4–6 cross veins on each side. Cubital field with four intercalaries connected by several cross veins; CuA and CuP connected by a single cross vein proximally; iCu2 longest, connected with CuA distally; Cu–A angle smoothly curved; CuP approaching A1; no cross veins in anal field (Fig. 2a).

*Hind wings* hyaline, translucent, relatively wide, 3.74–3.81 × shorter than forewings, with width/length ratio 0.54. Longitudinal venation is light brown; cross venation is well developed and relatively numerous, whitish yellow to yellow, and is poorly recognizable. Costal process of hind wings widely rounded, semilunar and strongly symmetric, located in middle of wing length, slightly rising above inner margin (Fig. 2a–b). Numerous cross veins between C and RA; RS fork well developed, slightly asymmetrical, iRS connected to Rsa and RSp by 7 cross veins; MA and MP not forked, 8 cross veins between MA and MP; cubital triad well-developed; veins of cubital field not touched; AA not forked (Fig. 2a–b).

*Legs*. Femora and tibiae brown, tarsi paler, yellowish brown to yellow distally. Forefemora covered with artificial, small, dark brown to blackish maculations [*as a result of specimen fossilization*]. Ratio of foreleg segments: 0.57 : 1.00 : 0.05 : 0.38 : 0.42 : 0.30 : 0.09. Patellotibial suture present on middle and hind legs, absent in foreleg. First tarsomere of middle and hind legs fused with tibia. Pretarsal claws with outer claw hooked and inner claw blunt.

*Abdominal segments* (Figs. 1b–c, 3 and Fig. 5) completely preserved, partly translucent, with last three segments darkest ones. No vestigial gill sockets and bases on lateral terga. Abdominal segments without large and prominent posterolateral projections (Fig. 5); three last abdominal segments not elongated compared to anterior segments (Fig. 5). Abdominal sterna are light brown; sternum I darkest, dark brown; no trace of coloured nerve ganglia (Fig. 1b–c). Preserved right cercus brown proximally, yellow to light yellow distally; terminal filament completely preserved, slightly longer and darker than cercus.

**Table 1:** Measurements of the holotype of *Calliarcys antiquus* **sp. nov.** (male imago; BB 2515; SMNS coll.).

Characters	(mm)
Length of body	5.40
Length of left foreleg	5.88
Length of femur	1.20
Length of tibia	2.10
Length of tarsus	2.58
Segment I	0.10
Segment II	0.80
Segment III	0.88
Segment IV	0.62
Segment V	0.18
Length of right middle leg	2.53
Length of femur	0.90
Length of tibia	1.05
Length of tarsus	0.58
Segment I	0.05
Segment II	0.15
Segment III	0.12
Segment IV	0.12
Segment V	0.14
Length of left middle leg*	0.84
Length of femur	0.35
Length of tibia	0.30
Length of tarsus	019
Length of right hind leg	2.64
Length of femur	0.96
Length of tibia	1.15
Length of tarsus	0.53
Segment I	0.06
Segment II	0.12
Segment III	0.09
Segment IV	0.10
Segment V	0.16
Length of right forewing	5.68
Length of left forewing	5.72
Length of right hind wing	1.52
Length of left hind wing	1.50
Hind/Fore wings length ratio	0.27
Length of right cercus	6.10
Length of terminal filament	7.70

*Malformed leg as a result of nymphal leg regeneration.

**Table 2:** The summary of morphological characters of the male imagines to distinguish extant and extinct representatives of the genus *Calliarcys*. Distinct differential characters are marked in bold.

Characters (male imago)	*Calliarcys antiquus* sp. nov.	*Calliarcys humilis*	*Calliarcys van*
*Head*			
Compound eyes colour: upper portion	–	**Creamy to orange pink**	**Brick-red**
Compound eyes colour: lower portion	–	Black to blackish grey	Black to blackish grey
Compound eyes: shape	**Distinctly large, contiguous**	**Middle-sized, widely separated**	**Large, separated**
***Wings: cross venation***	**Well-developed**	**Ordinarily developed**	**Ordinarily developed**
*Forewing*			
*Width/length ratio*	0.38	0.35–0.38	0.36–0.38
**Pterostigmatic venation**	**6–7 forked and 2–3 simple veins**	**8–12 simple veins**	**6–8 simple veins**
**Cubital venation: longest vein**	**iCu2**	**iCu3**	**iCu3**
*Hind wing*			
**Ratio to forewing length**	**3.74–3.81**	**3.30–3.60**	**3.8–4.0**
Width/length ratio	0.54	0.50–0.54	0.52–0.54
**Costal process: location**	**Middle of wing length**	**Before middle of length**	**Middle of wing length**
**Costal process: shape**	**Widely rounded apically**	**Step-like, acute apically**	**Smoothly rounded apically**
**Number of cross veins between MA and MP**	**8**	**1–2**	**1–2**
**Number of cross veins between MP and CuA**	**2**	**0**	**0**
**Cubital triad**	**Complete**	**Not complete**	**Not complete**
*Abdomen*			
Colour of terga	–	**Uniformly brown to dark brown**	**Yellowish-brown strip anteriorly**
*Genitalia*			
**Median projections of styliger: shape**	**Nearly finger-like**	**Finger-like**	**Nearly triangular**
**Forceps segment I: inner hump**	**Present**	**Absent**	**Absent**
**I. Penis lobes: general shape**	**(?) separated basally**	**Fussed basally**	**Fussed basally**
**II. Penis lobes: general shape**	**Relatively robust, shortened**	**Relatively elongated**	**Relatively robust, shortened**
III. Penis lobes: shape of tip	**Slightly bent inward**	**Distinctly bent inward**	**Slightly bent inward**

*Genitalia* (Figs. 1f–g, 2c, 3 and 5) only visible laterally, ventral side of body is covered by cercus and terminal filament. Due to the position of the specimen in the resin as well as cracks and streaks in the resin, styliger plate, base of forceps, and penis lobes are hardly visible in optical view (Fig. 1a,b,f–g). Styliger plate brown, deeply incised mediocaudally by a V-shaped incision; two median, nearly finger-shaped projections, markedly protruding above the anterior margin of the styliger; projections relatively large, rounded apically (Fig. 2c and 5d–e). Forceps base dark brown to brown; distal segments of forceps slightly paler. Forceps 3-segmented; ratio of forceps segments: 1.00: 0.21: 0.18; segment I strongly elongated, with distinct rounded hump basally, without prominent appendages; segment II relatively short with convex inner margin, slightly longer than segment III; distal segment of forceps (i.e. segment III) nearly oval, rounded apically (Figs. 1f–g, 2c and 5). Simple, straight and tubular, slightly club-shaped, penis lobes separated basally and touching apically; tip of penis only slightly bent inward, not obliquely truncated, without appendices or processes (Figs. 1f–g, 2c and 5).

### Female imago, male and female subimago and nymph

Unknown.

### DNA barcode data for the extant *Calliarcys humilis* and gap analysis for Leptophlebiinae and Habrophlebiinae

DNA barcoding proved that seven specimens of *C. humilis* belong to one BIN (BOLD:AEG9529), with two other specimens from Spain previously deposited in BOLD as private data. We identified six haplotypes within our sequences and the maximum K2p distance between the specimens did not exceed 1.86%. The sequences of *C. humilis* cluster among representatives of the Leptophlebiinae subfamily.

We analysed 104 species from the subfamilies Leptophlebiinae and Habrophlebiinae, for which we found only 39 species (37.5%) with public barcodes and BINs assigned. Number of sequences per species varied from one (e.g. *Paraleptophlebia gregalis*, *Neoleptophlebia vaciva*, *Habrophlebiodes zijinensis*, *Habroleptoides modesta*, *Habroleptoides pauliana*) to the maximum of 147 for *Neoleptophlebia mollis* with the mean value of 8.4 barcodes per species. We observed a maximum intraspecific distance higher than 3% in case of 19 species. The highest distance was observed among undetermined individuals of *Leptophlebia*, *Paraleptophlebia*, *Habroleptoides* (24.3%, 28.47%, and 28.25% respectively). A maximum intraspecific distance lower than 3% was observed in case of 13 species (Tables 3 and S1).

**Table 3:** Overview of barcoding statistics and molecular distances based on the Kimura 2-parameter model of the analysed specimens of the analysed Leptophlebiinae and Habrophlebiinae. BINs are based on the barcode analysis from 20.06.2022.

Subfamily	Genus	No of species	Species with barcodes	No of barcodes	No of BINs	Mean Intra-Sp	Max Intra-Sp
Leptophlebiinae	Leptophlebia	11	5	226	10	0.29–5.58	1.16–14.34
		Leptophlebia sp.	–	30	5	15.63	24.3
	Paraleptophlebia	34	14	184	26	0.45–20.6	1.03–26.07
		Paraleptophlebia sp.	–	28	13	22.32	28.47
	Neoleptophlebia	19	8	289	14	0–11.01	0–22.31
	Habrophlebiodes	8	2	37	2	1.67	4.2
	Dipterophlebiodes	1	0	0	0	0	0
	Gilliesia	3	0	0	0	0	0
	Calliarcys	2	1	7	1	0.97	1.86
Habrophlebiinae	Habrophlebia	8	3	43	6	0.35–6.97	1.54–15.08
	Hesperaphlebia	1	1	31	2	1.16	15.08
	Habroleptoides	17	5	57	17	0.15–9.1	0.15–23.96
		Habroleptoides sp.	–	9	3	11.14	28.25

## Discussion

### Taxonomy

We attribute *C. antiquus* **sp. nov.** to Leptophlebiidae based on the characteristic appearance of its mesonotal suture, which is transverse centrally, distinctly curved posteriorly, and stretching backward, and based on the furcasternal protuberance, which is clearly separated along its entire length. In addition to these characteristics, the presence of an asymmetric MA in the forewing in combination with a strongly curved CuP and distinct cubital intercalaries also indicate a systematic position of this extinct species within Leptophlebiidae (Figs. 1c, 2a, 3; Table 2).

The placement of *C. antiquus* **sp. nov.** within the genus *Calliarcys* can be verified by the presence of four free intercalaries in the cubital field of the forewing and the well-developed costal process at half length of the hind wing (Fig. 2b). Furthermore, the generic attribution is clearly justified by the characteristic shapes of styliger plate and genitalia as revealed by tomography: **(i)** simple and tubular penis lobes, deprived of a blade-like process at the tip; **(ii)** posterior margin of the styliger plate with V-shaped medial incision, and two long submedian projections; **(iii)** segment I without inner process or appendages, and **(iv)** forceps segments II and III shorter than the first segment, terminal segment shortest (Figs. 3c, 5; Table 2).

**Figure 6:**
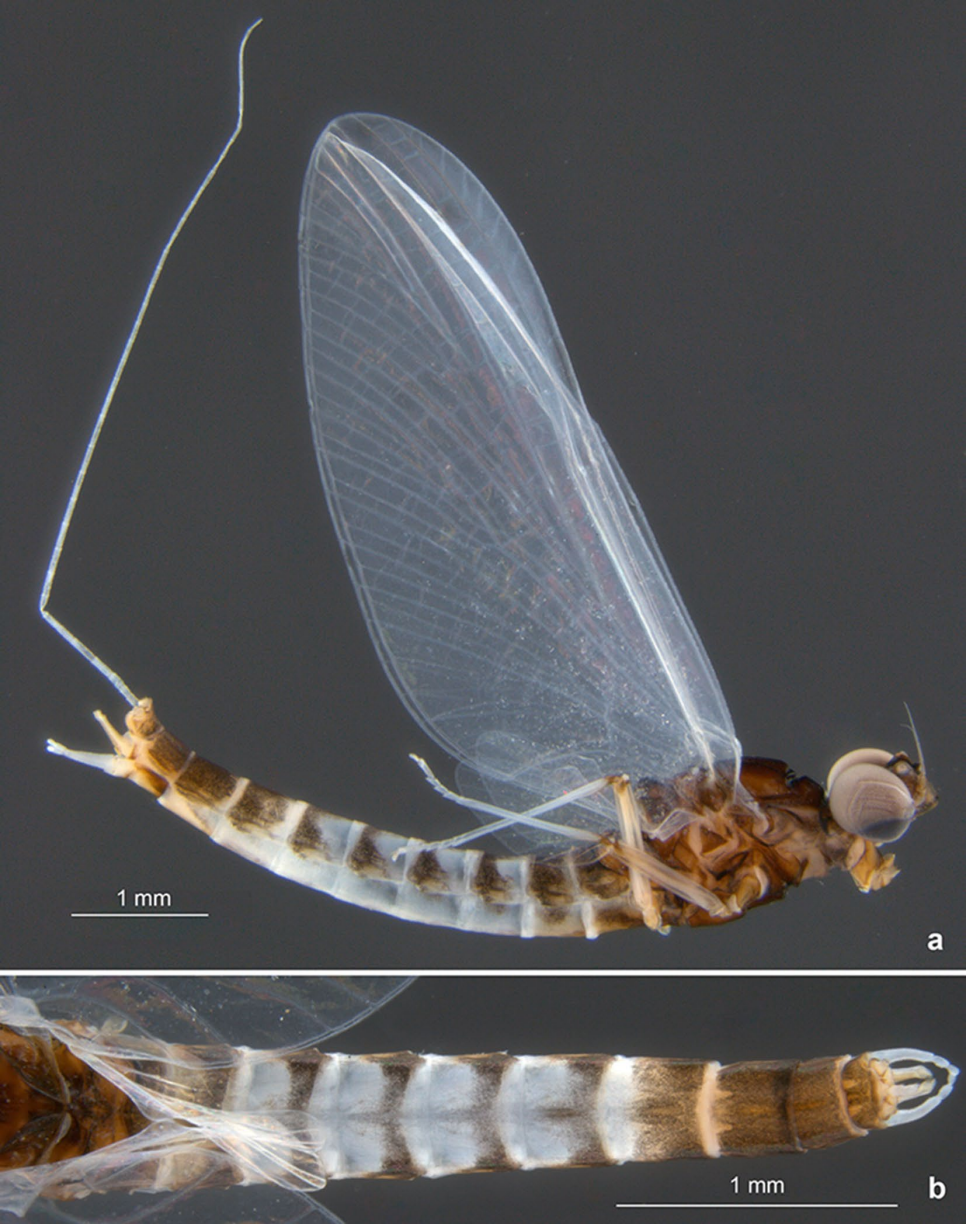
*Calliarcys van* Godunko & Bauernfeind, 2015***, ***male imago, paratype [Turkey; Soldán T. & Bojková J. coll.]: (**a**) body in right lateral view; (**b**) abdomen in dorsal view.

**Figure 7:**
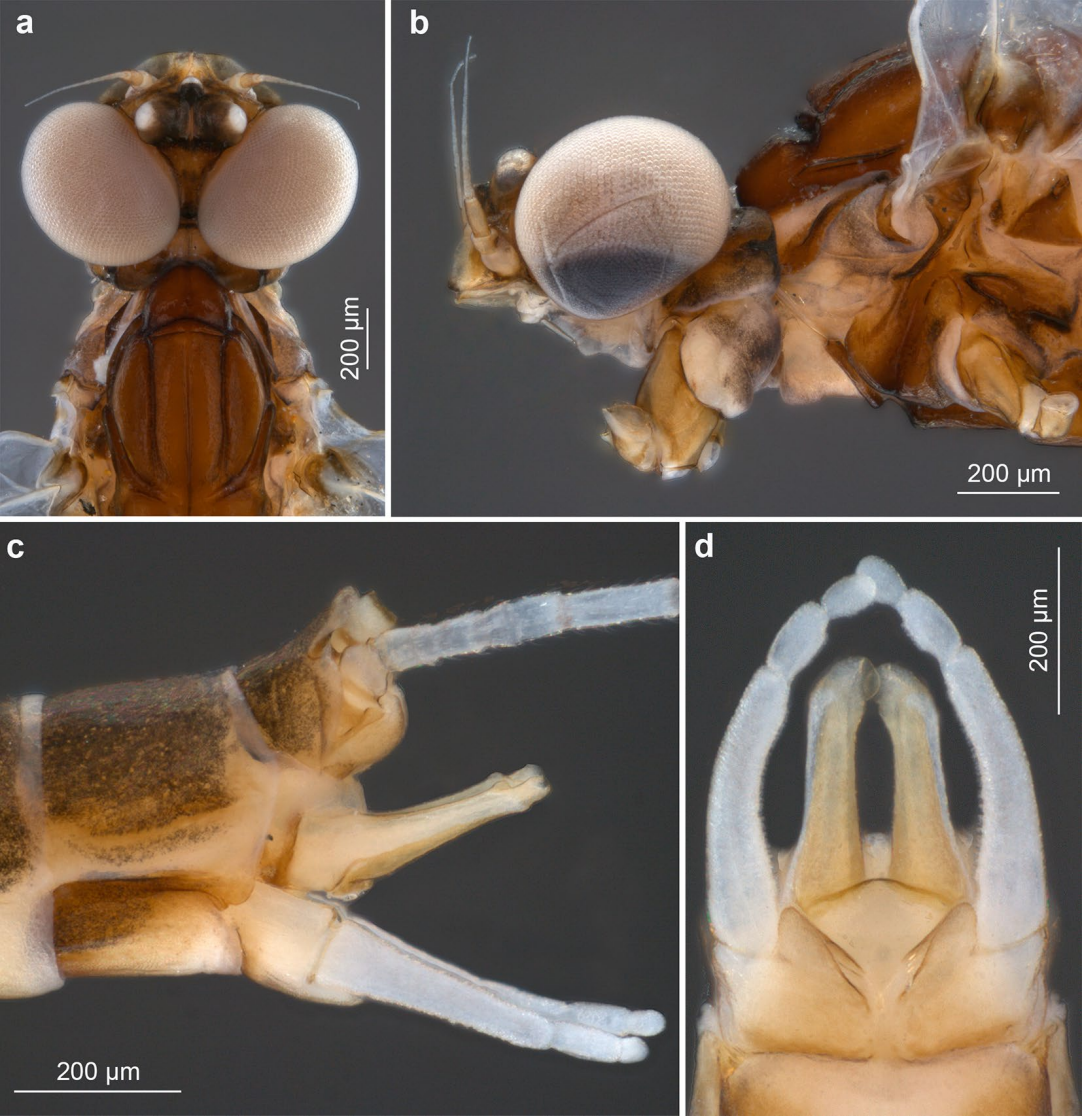
*Calliarcys van* Godunko & Bauernfeind, 2015***, ***male imago, paratype [Turkey; Soldán T. & Bojková J. coll.]: (**a**) head and anterior part of thorax in dorsal view; (**b**) head and anterior part of thorax in left lateral view; (**c**) tip of abdomen with genitalia in left lateral view; (**d**) genitalia in ventral view.

**Figure 8:**
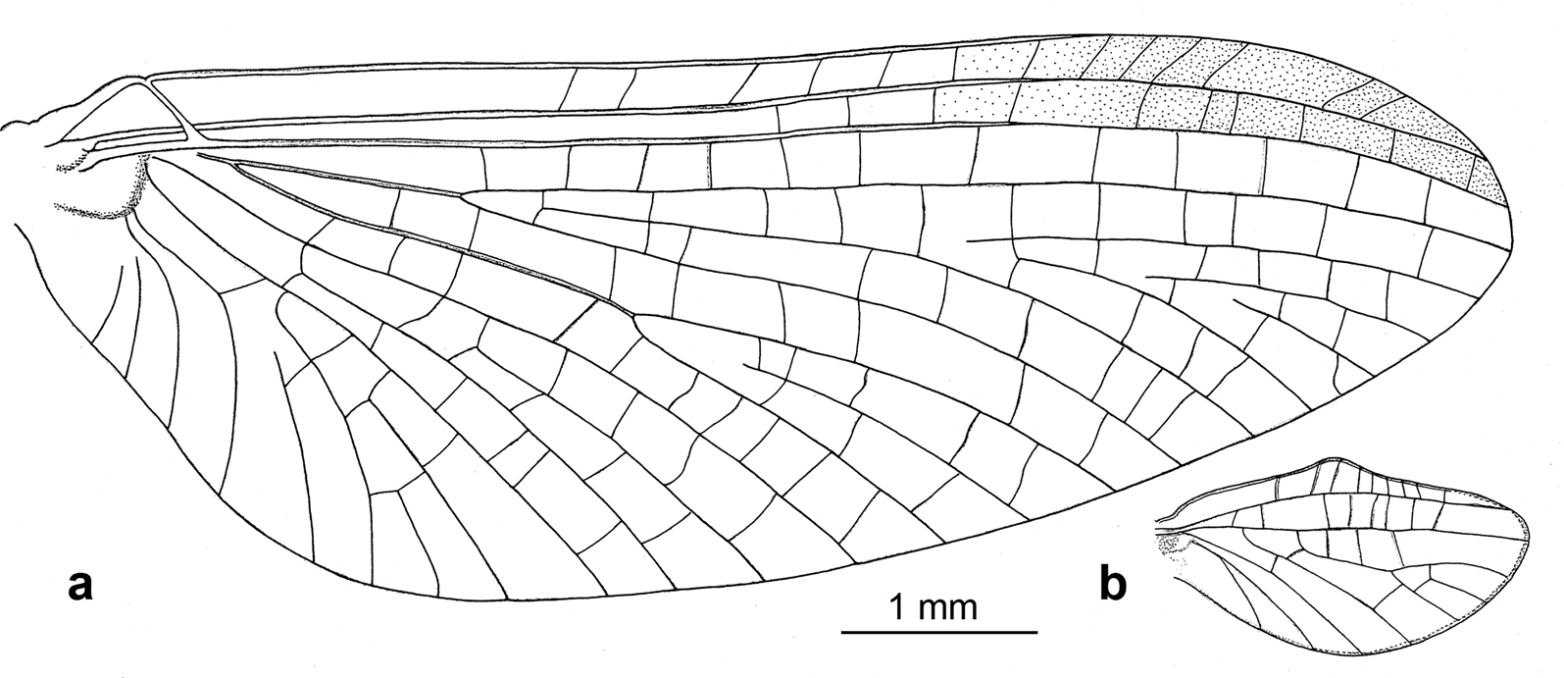
*Calliarcys van* Godunko & Bauernfeind, 2015***, ***male imago [Turkey; Soldán T. & Bojková J. coll.]: (**a**) forewing; (**b**) hind wing.

**Figure 9:**
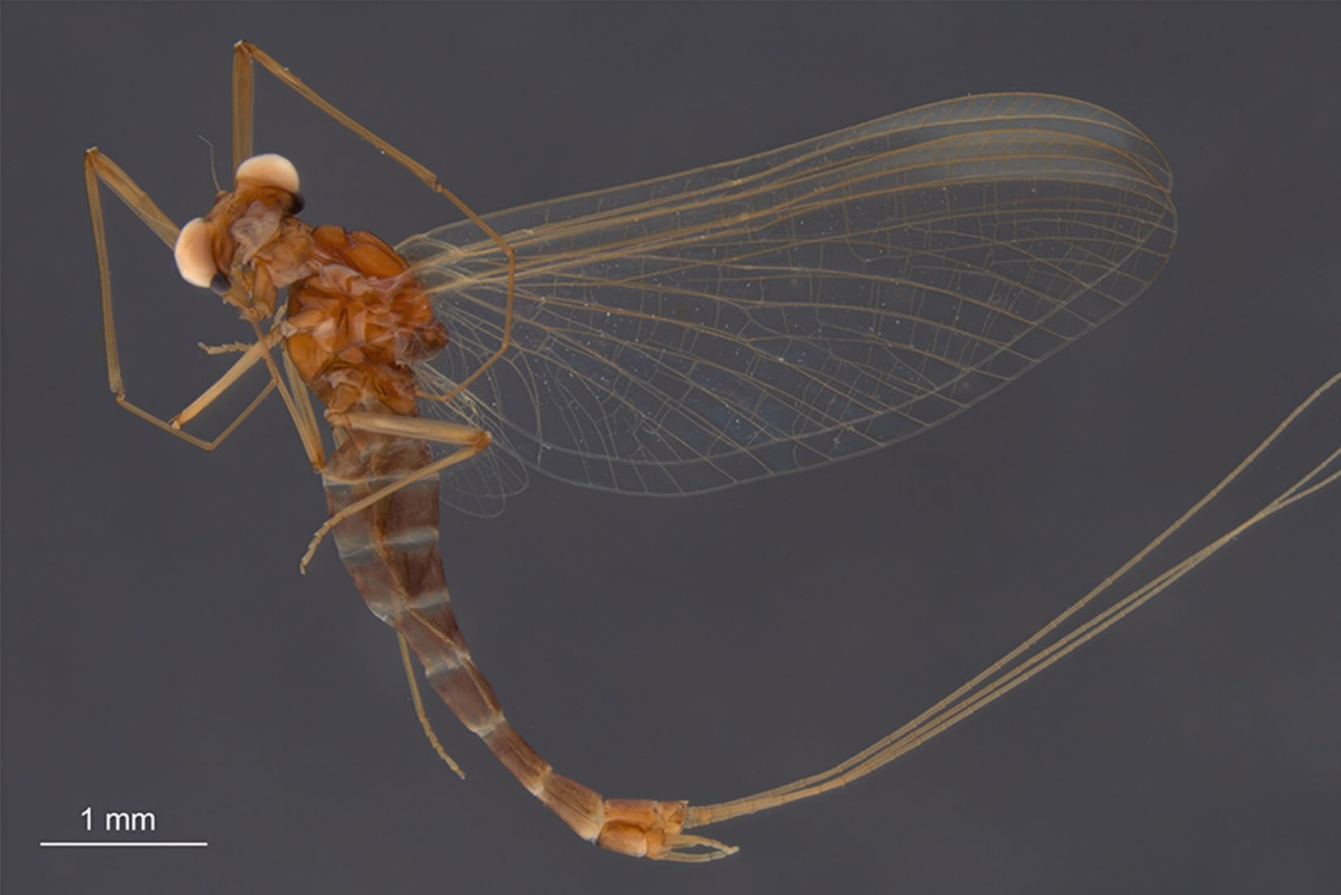
*Calliarcys humilis* Eaton, 1881, male imago [Spain; Sartori M. coll.].

The body colour is not a reliable character to separate the fossil and both extant species: Adults of *C. humilis* and *C. van* are both relatively dark to black, with a darker thorax, *C. antiquus* **sp. nov.** is generally light brown to brown, without any conspecific pattern on its abdominal terga. The colouration of the compound eyes can also not be used for species separation, but the shape of the upper eye portion differs in the males. In contrast to *C. humilis* and *C. van* with well-separated compound eyes (Figs. 6a, 7a–b, 8 and 9a,b), the upper portion in *C. antiquus* **sp. nov.** is clearly contiguous dorsally and also larger (Figs. 1e, 3a and 4b). In *C. humilis,* the upper portion is smallest, well separated, and the distance between the eyes is nearly as long as the width of the compound eye (Figs. 8 and 9a–b; for *C. van* see also Figs. 1, 4 and 5 in^2^).

**Figure 10:**
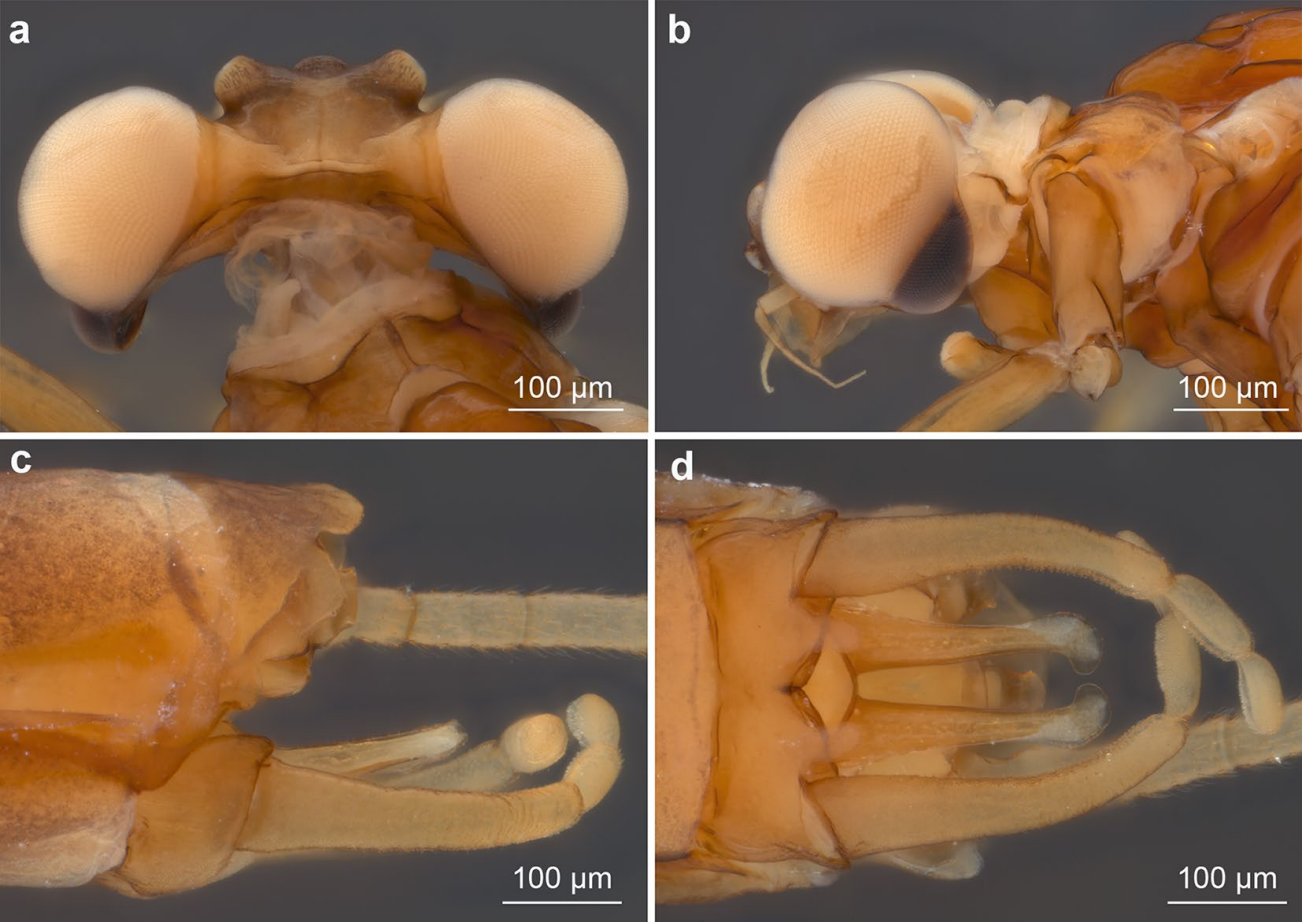
*Calliarcys humilis* Eaton, 1881, male imago [Spain; Sartori M. coll.]: (**a**) head and anterior part of thorax in dorsal view; (**b**) head and anterior part of thorax in left lateral view; (**c**) tip of abdomen with genitalia in left lateral view; (**d**) genitalia in ventral view.

**Figure 11:**
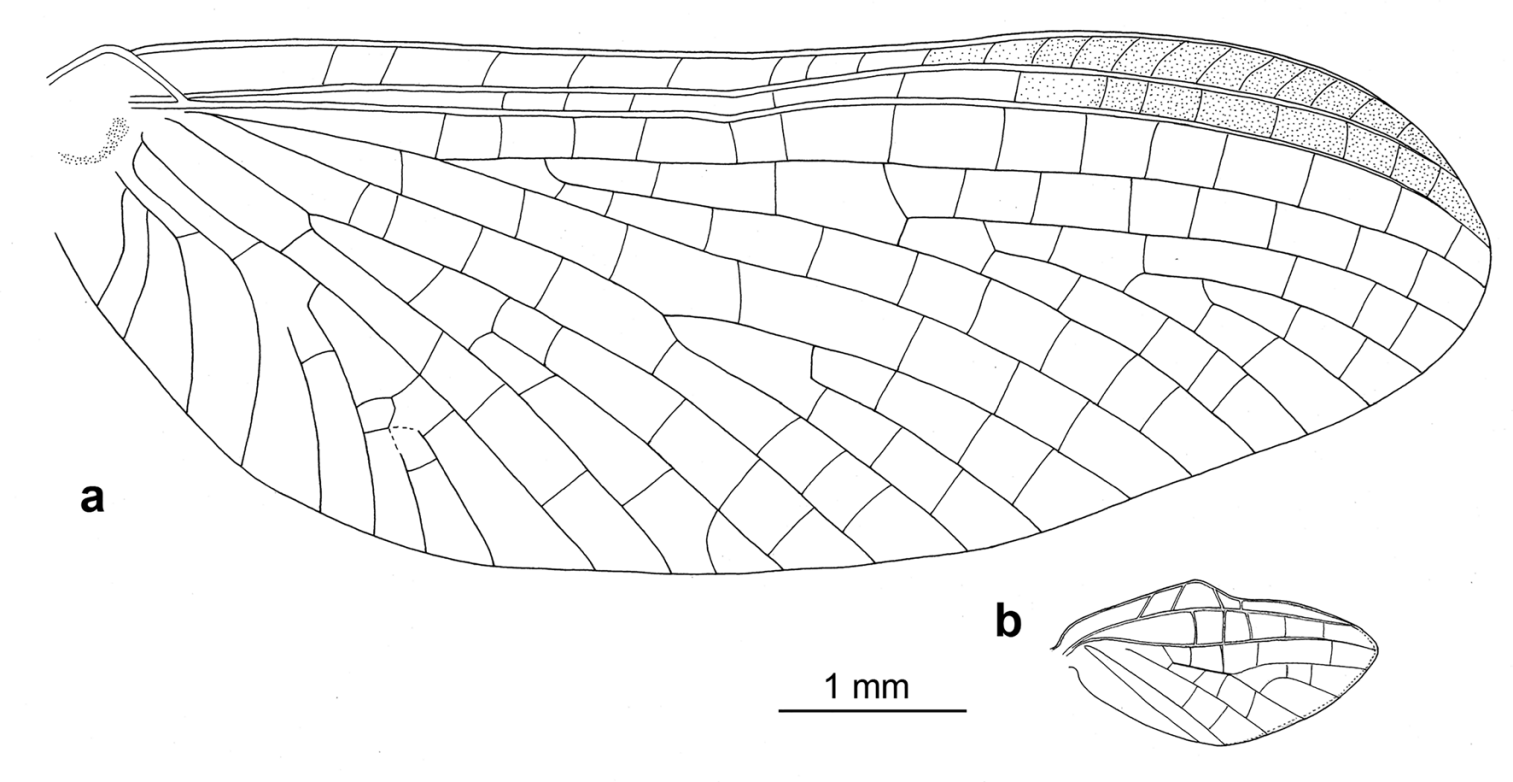
*Calliarcys humilis* Eaton, 1881, male imago [Spain; Sartori M. coll.]: (**a**) forewing; (**b**) hind wing.

The shape of the wing venation of all representatives of *Calliarcys* is rather similar, but in *C. antiquus* **sp. nov**., the cross venation of both wings is well-developed (Fig. 2a–b). Its male imago has predominantly forked veins in the pterostigmatic area of the forewing and the longest vein iCu2 (Fig. 2a), in contrast to simple pterostigmatic veins of *C. humilis* and *C. van* with the longest third vein [iCu3] in the cubital field (Figs. 10a, and 11a). While pterostigma and area between Sc and RA of the forewing are without any colouration, but translucent and hyaline in *C. antiquus* **sp. nov.** (Fig. 1b–c), this region is frosted and milky to yellowish white in both extant species (Figs. 6a and 8; see also Fig. 17 in^18^, Fig. 17 in^19^, Figs. 1 and 8 in^2^, and brief description in ^8^).

**Figure 12:**
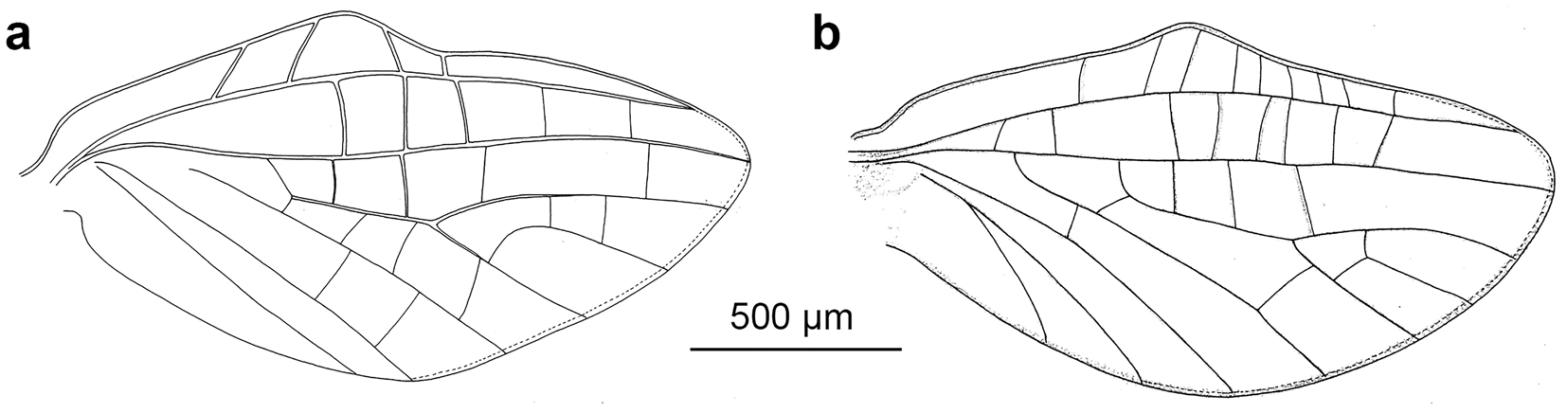
Hind wings of male imagines of (**a**) *Calliarcys humilis* Eaton, 1881 and (**b**) *C. van* Godunko & Bauernfeind, 2015.

The distal part of the hind wings in *C. humilis* and *C. van* seems to be narrower and elongated (Fig. 12), in contrast to a widely rounded distal half of the hind wings in *C. antiquus* **sp. nov.** (Fig. 2a–b). In *C. humilis*, the costal process is asymmetrical, nearly acute and step-like, situated in the proximal half of wing (Figs. 11b, Fig. 13a; for other drawings of *C. humilis* hind wings see Fig. 18 in^18^ and Fig. 18 in^19^), while it is nearly symmetric and rather at half length in *C. antiquus* **sp. nov.** and *C. van*^2^. Finally, the cubital venation in the hind wing is well-developed in *C. antiquus* **sp. nov.**, but diminished in the extant representatives.

Peters and Edmunds^19^ keyed out two generic adult characters of the genus *Calliarcys*, in particular, the presence of two hooked pretarsal claws of each pair of legs, with an ‘opposing hook’. In fact, all representatives of this genus possess dissimilar pretarsal claws, with one hooked and one triangular, somewhat rounded apically claw. Additionally, Nikita J. Kluge (see http://www.insecta.bio.spbu.ru/z/Eph-phyl/L_Calliarcys.htm) reported in both extant species the presence of a narrowed costal field in the apical half of the hind wing; we may add that in *C. antiquus* **sp. nov.** the costal field is also strongly narrowed (Figs. 2a–b, 10b, 11b and 12).

The male genitalia of the three species differ considerably from each other. While the penis lobes of *C. humilis* are elongated, relatively slender, and markedly stretched inward at their tip, the lobes of *C. van* and *C. antiquus* **sp. nov.** are relatively robust and shorter, with the tip only slightly bent inward (Figs. 2c, 5, 7c–d and 9c,d; for comparison see also Fig. 76 in^18^, Fig. 76 in^19^, Fig. 313 in^8^, and Figs. 6, 7 and 12–14 in^2^).

The submedian projections of the styliger plate are of similar shape in *C. antiquus* **sp. nov.** and *C. humilis,* namely elongated, slender, and finger-like, in contrast to broad, nearly triangular projections in *C. van*. An additional difference between the three species can be found in a distinct, rounded basal hump at the inner margin of forceps segment I in *C. antiquus* **sp. nov.**, this hump is lacking in extant species (Figs. 2c, 5, 7c–d and 9c–d; for comparison see also Fig. 23 in^20^, Fig. 42 in^21^, Fig. 76 in^19^, Fig. 313 in^8^, and Figs. 6, 7 and 12–14 in^2^).

*Calliarcys antiquus* **sp. nov.** clearly differs from all described Eocene taxa of Leptophlebiinae listed in the Supplementary material S5 (Supplementary Information 5) by the shape of penis lobes, the proportions and shape of forceps segments, the structure of cubital venation of forewings, and the presence of the more prominent costal process of the hind wings (see Figs. S1 and S2; for the list of extinct Cenozoic Leptophlebiinae see Table S4).

### DNA barcoding

DNA barcoding is a well-established and powerful tool for gaining information on the taxonomic status of various organisms and conducting assignment for problematic specimens, immature life stages, and tissue samples^22–24^. Biomonitoring involving eDNA, DNA barcoding and metabarcoding starts to be implemented as a standard procedure^25,26^. Effectiveness of such methods depends on the reliability of barcode reference libraries for particular taxa, which are still far from complete^27,28^. Among freshwater macroinvertebrates, the Ephemeroptera, Plecoptera, and Trichoptera (EPT) faunas are most important in biomonitoring^29–31^. Thus our addition of properly identified specimens of *Calliarcys humilis* as one of two living representants of this genus is valuable. Interestingly, according to COI, *C. humilis* groups among various taxa within Leptophlebiinae, providing a first hint for the taxonomic position of the genus (see Supplementary material S4 in Supplementary Information 2). Additionally, our gap analysis showed a surprisingly high underrepresentation (39 from 104 species) of barcoded species within the subfamilies Leptophlebiinae and Habrophlebiinae (Table 3). It must be stressed that those 19 species, where the maximum K-2p intraspecific distance is higher than 3%, may represent either cases of previously undetected cryptic diversity or point to the misidentification of some individuals. The latter is most probable for at least 10 species, where we noticed BINs shared between al least two species. Another, less likely option would be that one or more species within one BIN is not a real species, but e.g. just a morph or a hybrid. It is worth to stress that such misidentifications in open databases like GenBank and BOLD still persist, even if there are attempts for better data curation, and for removing obvious mistakes^32–34^. Given the number of EPT taxa and their importance for ecosystem health assessments, an inaccurate taxonomic assignment of DNA barcodes can seriously hamper the reliability of biomonitoring of water ecosystems based on molecular data. Thus pointing out such mistakes is crucial to improve the situation.

## Materials and Methods

### Material

The holotype of fossil *Calliarcys antiquus* **sp. nov.** described in this study is housed in the collection of the State Museum of Natural History, Stuttgart (SMNS) under the inventory number BB 2515 (holotype; male imago). The piece of resin originates from an unknown Eocene deposit of Baltic amber.

The investigated specimens of *Calliarcys van* belong to the type series (holotype and paratypes of male imagines) collected in Turkey in 2011 by Tomáš Soldán and Jindřiška Bojková (see also Godunko et al.^2^). This material was used for morphological comparison with extinct species.

Extant *C. humilis* from the collection of the Musée Cantonal de Zoologie, Lausanne, Switzerland, were used for morphological comparison:

2 male imagines, Spain, Galicia, Provincia de Lugo, Rio Labrada, close to the village of Insua, approximately 43°14’N/7°43’W, 13.vi.1985, Michel Sartori coll.].

Additional material of *C. humilis* (adults of both sexes and nymphs), deposited in the collections of the Biology Centre CAS, Institute of Entomology, České Budějovice, Czech Republic (IECA) and the State Museum of Natural History, Stuttgart, Germany (SMNS), was used for DNA extraction and comparative morphological studies:

6 male imagines, 2 male subimagines, 5 female imagines, 2 female subimagines, Spain, Rio Garganta de Los Caballeros, small stream in granitic rocks of the Tormellas granitic rocks, Ávila, Sistema Central located in W of Central Spain, 40°18’N 5°30’W, 1030 m asl., 23.iv.2021, José Ángel Martín del Arco coll.

16 nymphs, Spain, Salamanca, Rio Cuerpo de Hombre, locality in Candelario, little stream in granitic rocks of the Sierra de Béjar, Sistema Central located in the W of Central Spain, 40°22’N 5°45’W, 1050 m asl., 24.iv.2021, José Ángel Martín del Arco coll.

All specimens of extant *Calliarcys* are preserved in 96% ethanol.

### Morphological and morphometric studies

Some paratypes of *C. van* and some of the imaginal material of *C. humilis* were mounted on slides with Liquide de Faure (soluble in water). The material of extant specimens was observed using Olympus SZX7 stereo microscope and Olympus BX41 microscope for microslides.

Observation and drawings of *C. antiquus* **sp. nov.** were made by using Micro-CT-based reconstruction and by a camera lucida on a Leica M205 C stereo microscope. Multiple photographs with different focal depths were taken with a Leica DFC450 Digital Camera through a Leica Z16 101 APO Macroscope using Leica Application Suite v. 3.1.8. The photo stacks were processed with Helicon Focus Pro 6.4.1 to obtain combined photographs with extended depth of field and subsequently enhanced with Adobe Photoshop Classic.

The measurements of individual body parts were taken either by using an ocular grid or inferred from the photographs taken with a calibration scale. The measurements of *C. antiquus* **sp. nov.** are given in Table 1. Identical morphometric parameters were used also for other Mesozoic and Cenozoic mayfly fossils (see e.g.^35,36^).

Morphological terminology and nomenclature of wing veins used throughout the text follow Kluge^7,37^ and Bauernfeind and Soldán^8^; thoracic morphology is analysed using following contributions^37–40^.

### Micro-CT scans and image reconstruction

The piece of amber containing the fossil was fixed to the sample holder with plasticine and scanned using a Bruker SkyScan 1172 microtomograph (Bruker-micro CT, Kontich, Belgium) with a Hamamatsu 80/100 X-ray source and a VDS 1.3Mp camera. The setting parameters were as follows: voltage = 69 kV; current = 89 µA; isotropic voxel size = 4.05 µm; image rotation step = 0.2°; 360° of rotation scan without filter. This resulted in a scan duration of 5 h:04 min:04 s and 1802 2D shadow projections (X-ray images).

### Image reconstruction

Tiff X-ray projections images resulting from the scanning process were further processed with different Bruker microCT’s Skyscan software: reconstructed with NRecon v.2.0.0.5; CTAnalyser v.1.20.8.0 was used for primary ‘cleaning’ process, and resulting images were reoriented with DataViewer v.1.6.0.0; and finally, CTvox v. 3.3.1 was used to get 3D rendered images of Supplementary video S3, a detailed explanation of the procedure was previously published^41^. However, the reconstruction revealed that no internal structures were visible and only a thin cuticular layer is preserved. The latter showed an X-ray transparency similar to that of the surrounding amber matrix. This prevented an appropriate volumetric visualisation of the insect. Thus, we followed a procedure (for details see Supplementary Information S1). Amira 6.7.0 (Thermo Fisher Scientific, Waltham, MA)^42,43^ with the built-in ‘volrenWhite.am’ colour filter that aimed at visualising only the thin cuticular surface layer (or the impression left in the amber), but eliminating the rest of the amber matrix. An additional cleaning procedure was performed manually with the Amira module ‘Volume Edit’ before obtaining volume-rendered images (Figs. 4–7) and recording Supplementary Video S2.

### Molecular techniques

*DNA barcode amplification, sequencing, dataset assemblage and analysis.* Total DNA was extracted from one or two legs of each species used, depending on the size of the samples, using Genomic Mini Kit (A&A Biotechnology, Gdansk, Poland) according to the manufacturer’s protocol. COI fragments were amplified using LCO1490-JJ/HCO2198-JJ primers^44^, using reaction conditions after Hou et al.^45^. The success of the reaction was verified using a standard agarose gel. PCR products were purified following the procedure described by Rewicz et al.^46^. Bidirectional Sanger sequencing was outsourced by Macrogen Europe (Amsterdam, The Netherlands). The identity of the obtained sequences was verified with BLAST^47^. Double-stranded sequences were checked, aligned, and trimmed to the standard length 658 bp using Geneious 10.2.6 software package^48^. We also checked for the absence of frameshifts, double peaks, and stop codons using the Geneious 10.2.6 software package^48^. Sequences were deposited in GenBank (https://www.ncbi.nlm.nih.gov/genbank) under accession numbers OM158449–OM158451 and OM158454–OM158457. Relevant voucher information, photos, taxonomic classification, and DNA barcode sequences are also publicly accessible through the dataset DS-RGCAL (https://doi.org/10.5883/DS-RGCAL) in BOLD (www.boldsystems.org)^49^. Obtaining BINs (Barcode Index Numbers) for sequences deposited in BOLD provided additional verification of species identification, as BINs can be treated as tentative equivalents of species^50^. To conduct gap analysis and the general molecular diversity, we searched BOLD public repository for members of subfamilies Leptophlebiinae and Habrophlebiinae. Additionally, the first author prepared a checklist of 104 described species from those subfamilies, and we searched the BOLD repository again (https://v4.boldsystems.org under accession https://doi.org/10.5883/DS-RGCAL) (see Supplementary Information 3 and 4; Tables S1 and S2). The obtained unchecked database was curated, and we discarded all specimens either without COI sequence, with stop codons, contaminations or other flags added. We also discarded specimens with COI sequence shorter than 500 bp and without BIN assigned. After such curation, the final dataset contained 941 sequences (see details in: Tables S1 and S2; data set DS-RGCAL). The intraspecific mean and maximum genetic distances were calculated based on the Kimura 2-parameter model (K2P;^51^), using the analytical tools of the BOLD workbench (Barcode Gap Analysis, Distance Summary) and MUSCLE alignment method^52^.

The Barcode Gap analysis was performed for individuals identified to species level, while the Distance Summary Tool was applied to specimens determined only to the genus level. We did not evaluate if the identification of the specimens to the species level was correct. Only in the case of unidentified specimens with BIN, which was matching to BIN already assigned to identified species, we added them to identified species. To illustrate the molecular diversity of the analysed taxa, we prepared BOLD TaxonID Tree for COI sequences from our dataset DS-RGCAL (see Supplementary Information 2).

## Supplementary Information

Supplementary Information 1.

Supplementary Video 1.

Supplementary Video 2.

Supplementary Information 2.

Supplementary Information 3.

Supplementary Information 4.

Supplementary Information 5.

Supplementary Information 6.

## Data Availability

All sequences used in this study are publicly available in the BOLD dataset DS-RGCAL under the link http://dx.doi.org/10.5883/DS-RGCAL. All newly generated sequences of *Calliarcys humilis* were also deposited on GenBank (accession numbers: OM158449—OM158451, OM158454—OM158457).
