# Supplementary material for: Cenozoic origins of the genus Calliarcys (Insecta, Ephemeroptera) revealed by Micro-CT, with DNA barcode gap analysis of Leptophlebiinae and Habrophlebiinae
Source: Sci Rep. 2022 Sep 8;12:15228. doi: 10.1038/s41598-022-18234-4 (PMC9458648; doi:10.1038/s41598-022-18234-4)
Supplement: Supplementary file 1 — Supplementary Information 1. [file 41598_2022_18234_MOESM1_ESM.pdf]

DETAILED PROCESS TO OBTAIN THE SURFACE OF THE MAYFLY IN AMBER FOSSIL

Prof. Javier Alba-Tercedor. Univ. of Granada. Spain. jalba@ugr.es

A.- AFTER THE RECONSTRUCTION (WITH THE SOFTWARE NRecon):

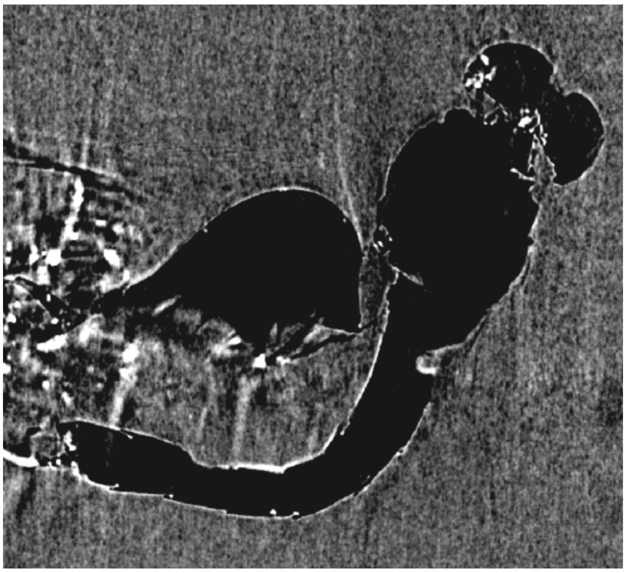

In the reconstructed images the body of the insect appears empty, and the cuticular outer layer can be observed. Left: dorsal-ventral slice. Right: dorsal-ventral slice

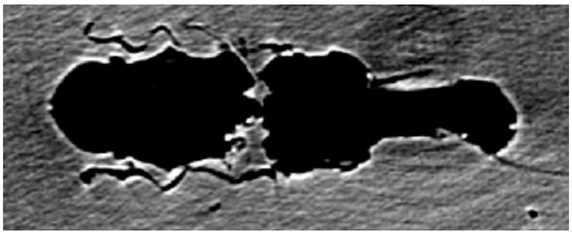

B.- BY A SEQUENTIAL USE OF DIFFERENT PLUGINS WITHIN THE SOFTWARE CTANALISER (CTAN)

IT CAN BE OBTAINED CLEAN CROSS-SECTION IMAGES SUITABLE TO PRODUCE REASONABLY GOOD VOLUME RENDERED **IMAGES:**

For each consecutive step the image on the left shows the plugin setting parameters and the image on the right shows the resulting image after running the plugin.

1.- Thresholding

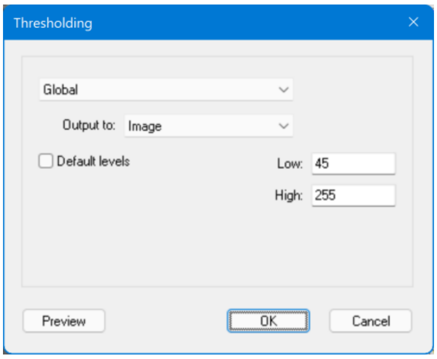

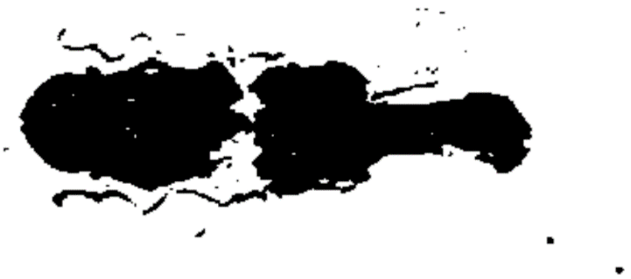

2.- Bitwise operations

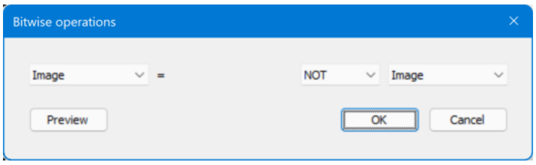

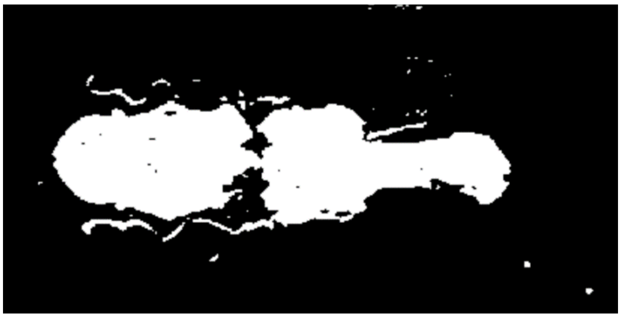

3.- **Despeckle (Sweep)**:

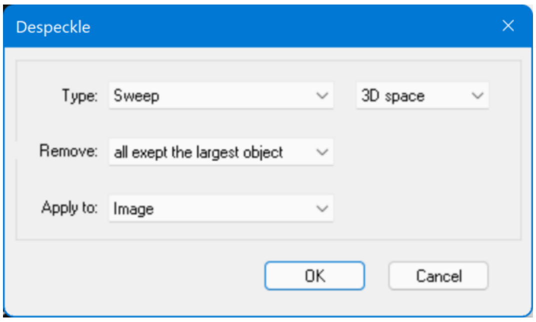

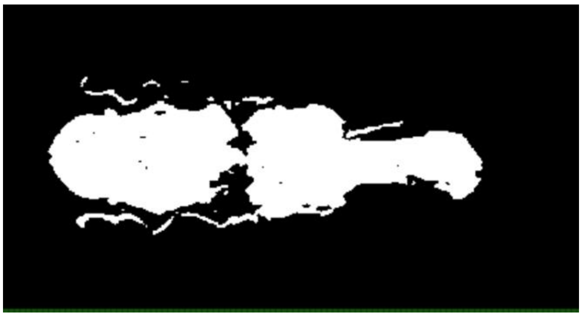

4.- **ROI shrink-wrap**

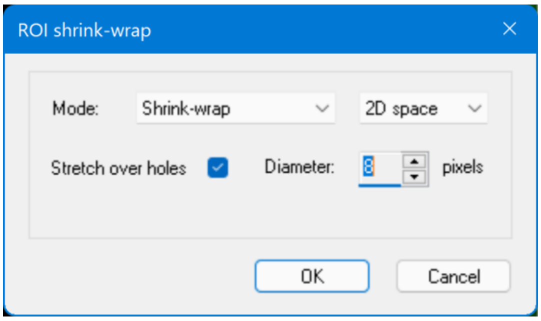

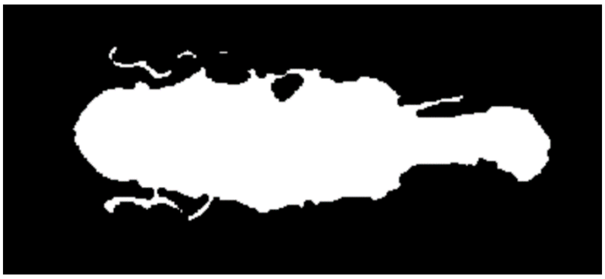

6.- **Morphological operations (Dilation 2D space)**:

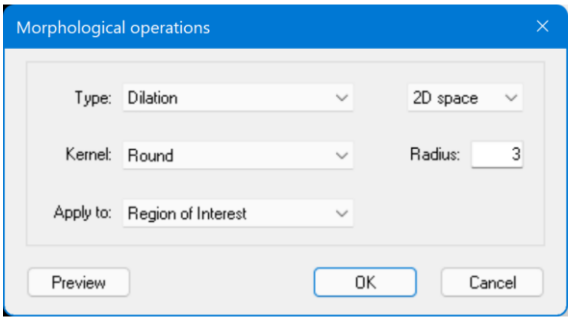

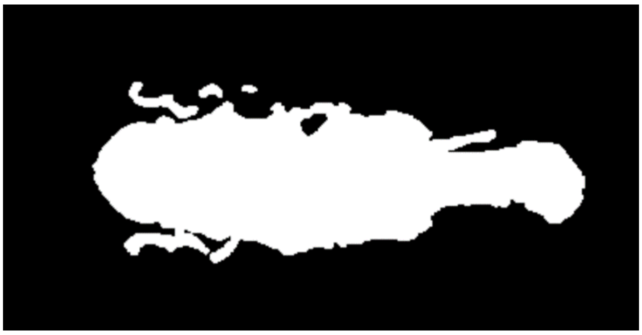

8.- **Reload image**

9.- **Save bitmaps (image inside ROI)**:

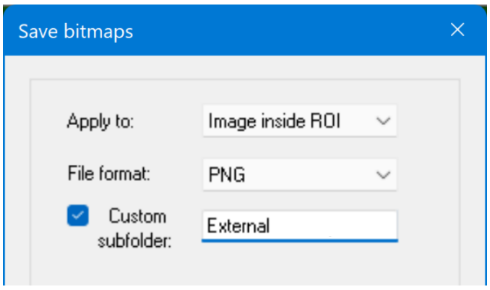

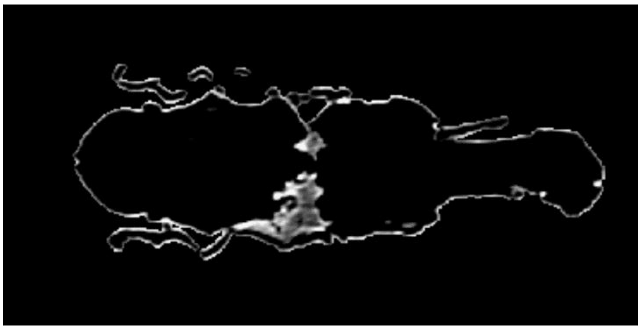

10.- **Upload images to a rendering software** (i.e.: Amira/CTVox) for visualisation.
