# Supplementary material for: Cenozoic origins of the genus Calliarcys (Insecta, Ephemeroptera) revealed by Micro-CT, with DNA barcode gap analysis of Leptophlebiinae and Habrophlebiinae
Source: Sci Rep. 2022 Sep 8;12:15228. doi: 10.1038/s41598-022-18234-4 (PMC9458648; doi:10.1038/s41598-022-18234-4)
Supplement: Supplementary file 4 — Supplementary Information 2. [file 41598_2022_18234_MOESM4_ESM.pdf]

BOLD TaxonID Tree

Supplementary material S4: BOLD TaxonID Tree of COI sequence from dataset DS-RGCAL (incl. *Calliarcys humilis*)

Title	:	Tree Result - DS-RGCAL
Date	:	20-Jun-2022
Data Type	:	Nucleotide
Distance Model	:	Kimura 2 Parameter
Marker	:	COI-5P
Colourization	:	[blue]=Stop Codons [red]=Contamination or misidentification

Label	:	Sample ID
Label	:	Process ID
Label	:	Taxon
Label	:	Country
Label	:	Barcode Cluster (BIN)
Label	:	GenBank Accession

Sequence Count	:	946
Species count	:	85
Genus count	:	8
Family count	:	1
Unidentified	:	26

BIN Count	:	101

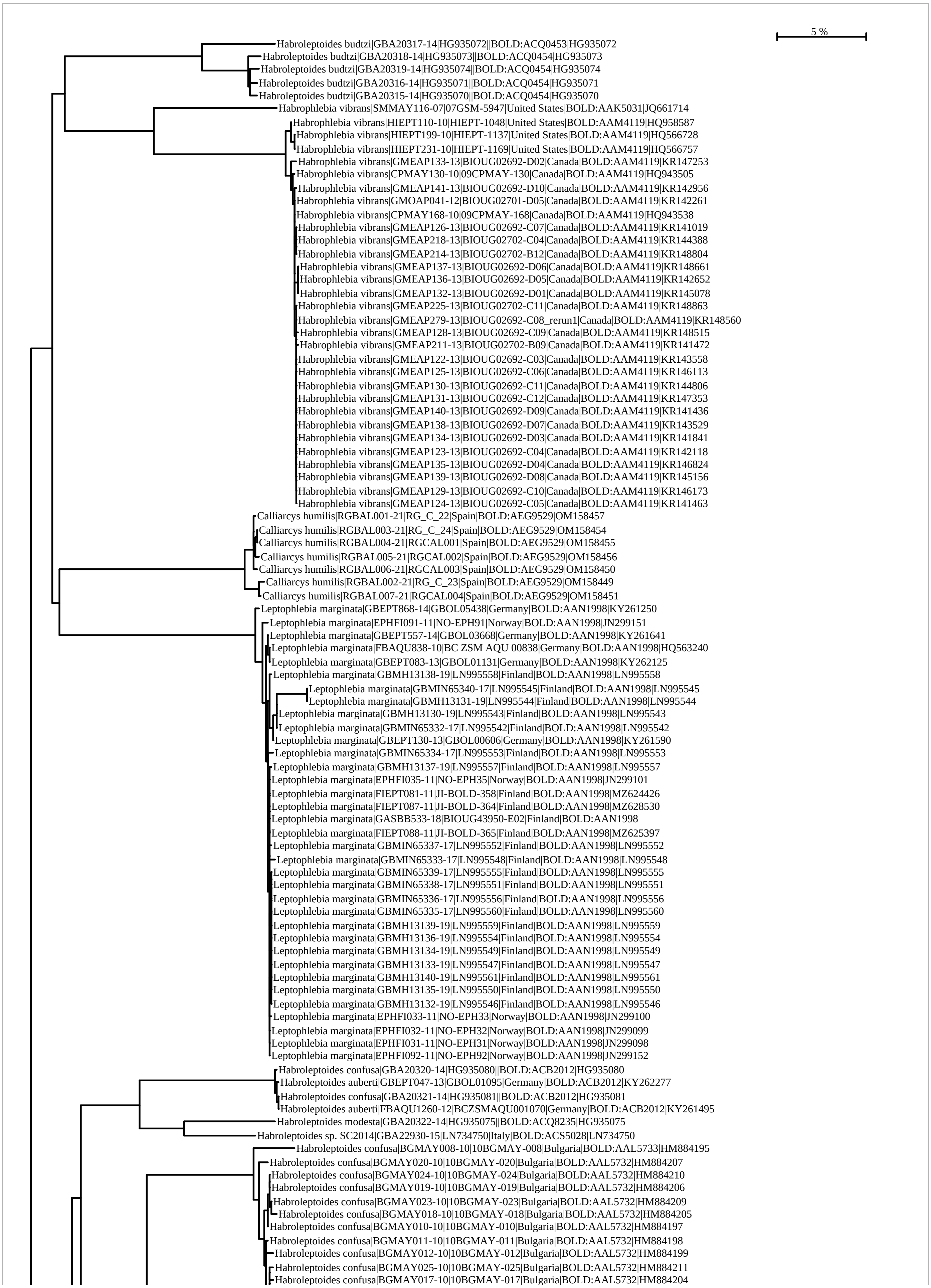
5 %
Habroleptoides budtzi|GBA20317-14|HG935072||BOLD:ACQ0453|HG935072
Habroleptoides budtzi|GBA20318-14|HG935073||BOLD:ACQ0454|HG935073
Habroleptoides budtzi|GBA20319-14|HG935074||BOLD:ACQ0454|HG935074
Habroleptoides budtzi|GBA20316-14|HG935071||BOLD:ACQ0454|HG935071
Habroleptoides budtzi|GBA20315-14|HG935070||BOLD:ACQ0454|HG935070
Habrophlebia vibrans|SMMAY116-07|07GSM-5947|United States|BOLD:AAK5031|JQ661714
Habrophlebia vibrans|HIEPT110-10|HIEPT-1048|United States|BOLD:AAM4119|HQ958587
Habrophlebia vibrans|HIEPT199-10|HIEPT-1137|United States|BOLD:AAM4119|HQ566728
Habrophlebia vibrans|HIEPT231-10|HIEPT-1169|United States|BOLD:AAM4119|HQ566757
Habrophlebia vibrans|GMEAP133-13|BIOUG02692-D02|Canada|BOLD:AAM4119|KR147253
Habrophlebia vibrans|CPMAY130-10|09CPMAY-130|Canada|BOLD:AAM4119|HQ943505
Habrophlebia vibrans|GMEAP141-13|BIOUG02692-D10|Canada|BOLD:AAM4119|KR142956
Habrophlebia vibrans|GMOAP041-12|BIOUG02701-D05|Canada|BOLD:AAM4119|KR142261
Habrophlebia vibrans|CPMAY168-10|09CPMAY-168|Canada|BOLD:AAM4119|HQ943538
Habrophlebia vibrans|GMEAP126-13|BIOUG02692-C07|Canada|BOLD:AAM4119|KR141019
Habrophlebia vibrans|GMEAP218-13|BIOUG02702-C04|Canada|BOLD:AAM4119|KR144388
Habrophlebia vibrans|GMEAP214-13|BIOUG02702-B12|Canada|BOLD:AAM4119|KR148804
Habrophlebia vibrans|GMEAP137-13|BIOUG02692-D06|Canada|BOLD:AAM4119|KR148661
Habrophlebia vibrans|GMEAP136-13|BIOUG02692-D05|Canada|BOLD:AAM4119|KR142652
Habrophlebia vibrans|GMEAP132-13|BIOUG02692-D01|Canada|BOLD:AAM4119|KR145078
Habrophlebia vibrans|GMEAP225-13|BIOUG02702-C11|Canada|BOLD:AAM4119|KR148863
Habrophlebia vibrans|GMEAP279-13|BIOUG02692-C08_rerun1|Canada|BOLD:AAM4119|KR148560
Habrophlebia vibrans|GMEAP128-13|BIOUG02692-C09|Canada|BOLD:AAM4119|KR148515
Habrophlebia vibrans|GMEAP211-13|BIOUG02702-B09|Canada|BOLD:AAM4119|KR141472
Habrophlebia vibrans|GMEAP122-13|BIOUG02692-C03|Canada|BOLD:AAM4119|KR143558
Habrophlebia vibrans|GMEAP125-13|BIOUG02692-C06|Canada|BOLD:AAM4119|KR146113
Habrophlebia vibrans|GMEAP130-13|BIOUG02692-C11|Canada|BOLD:AAM4119|KR144806
Habrophlebia vibrans|GMEAP131-13|BIOUG02692-C12|Canada|BOLD:AAM4119|KR147353
Habrophlebia vibrans|GMEAP140-13|BIOUG02692-D09|Canada|BOLD:AAM4119|KR141436
Habrophlebia vibrans|GMEAP138-13|BIOUG02692-D07|Canada|BOLD:AAM4119|KR143529
Habrophlebia vibrans|GMEAP134-13|BIOUG02692-D03|Canada|BOLD:AAM4119|KR141841
Habrophlebia vibrans|GMEAP123-13|BIOUG02692-C04|Canada|BOLD:AAM4119|KR142118
Habrophlebia vibrans|GMEAP135-13|BIOUG02692-D04|Canada|BOLD:AAM4119|KR146824
Habrophlebia vibrans|GMEAP139-13|BIOUG02692-D08|Canada|BOLD:AAM4119|KR145156
Habrophlebia vibrans|GMEAP129-13|BIOUG02692-C10|Canada|BOLD:AAM4119|KR146173
Habrophlebia vibrans|GMEAP124-13|BIOUG02692-C05|Canada|BOLD:AAM4119|KR141463
Calliarcys humilis|RGBAL001-21|RG_C_22|Spain|BOLD:AEG9529|OM158457
Calliarcys humilis|RGBAL003-21|RG_C_24|Spain|BOLD:AEG9529|OM158454
Calliarcys humilis|RGBAL004-21|RGCAL001|Spain|BOLD:AEG9529|OM158455
Calliarcys humilis|RGBAL005-21|RGCAL002|Spain|BOLD:AEG9529|OM158456
Calliarcys humilis|RGBAL006-21|RGCAL003|Spain|BOLD:AEG9529|OM158450
Calliarcys humilis|RGBAL002-21|RG_C_23|Spain|BOLD:AEG9529|OM158449
Calliarcys humilis|RGBAL007-21|RGCAL004|Spain|BOLD:AEG9529|OM158451
Leptophlebia marginata|GBEPT868-14|GBOL05438|Germany|BOLD:AAN1998|KY261250
Leptophlebia marginata|EPHFI091-11|NO-EPH91|Norway|BOLD:AAN1998|JN299151
Leptophlebia marginata|GBEPT557-14|GBOL03668|Germany|BOLD:AAN1998|KY261641
Leptophlebia marginata|FBAQU838-10|BC ZSM AQU 00838|Germany|BOLD:AAN1998|HQ563240
Leptophlebia marginata|GBEPT083-13|GBOL01131|Germany|BOLD:AAN1998|KY262125
Leptophlebia marginata|GBMH13138-19|LN995558|Finland|BOLD:AAN1998|LN995558
Leptophlebia marginata|GBMIN65340-17|LN995545|Finland|BOLD:AAN1998|LN995545
Leptophlebia marginata|GBMH13131-19|LN995544|Finland|BOLD:AAN1998|LN995544
Leptophlebia marginata|GBMH13130-19|LN995543|Finland|BOLD:AAN1998|LN995543
Leptophlebia marginata|GBMIN65332-17|LN995542|Finland|BOLD:AAN1998|LN995542
Leptophlebia marginata|GBEPT130-13|GBOL00606|Germany|BOLD:AAN1998|KY261590
Leptophlebia marginata|GBMIN65334-17|LN995553|Finland|BOLD:AAN1998|LN995553
Leptophlebia marginata|GBMH13137-19|LN995557|Finland|BOLD:AAN1998|LN995557
Leptophlebia marginata|EPHFI035-11|NO-EPH35|Norway|BOLD:AAN1998|JN299101
Leptophlebia marginata|FIEPT081-11|JI-BOLD-358|Finland|BOLD:AAN1998|MZ624426
Leptophlebia marginata|FIEPT087-11|JI-BOLD-364|Finland|BOLD:AAN1998|MZ628530
Leptophlebia marginata|GASBB533-18|BIOUG43950-E02|Finland|BOLD:AAN1998
Leptophlebia marginata|FIEPT088-11|JI-BOLD-365|Finland|BOLD:AAN1998|MZ625397
Leptophlebia marginata|GBMIN65337-17|LN995552|Finland|BOLD:AAN1998|LN995552
Leptophlebia marginata|GBMIN65333-17|LN995548|Finland|BOLD:AAN1998|LN995548
Leptophlebia marginata|GBMIN65339-17|LN995555|Finland|BOLD:AAN1998|LN995555
Leptophlebia marginata|GBMIN65338-17|LN995551|Finland|BOLD:AAN1998|LN995551
Leptophlebia marginata|GBMIN65336-17|LN995556|Finland|BOLD:AAN1998|LN995556
Leptophlebia marginata|GBMIN65335-17|LN995560|Finland|BOLD:AAN1998|LN995560
Leptophlebia marginata|GBMH13139-19|LN995559|Finland|BOLD:AAN1998|LN995559
Leptophlebia marginata|GBMH13136-19|LN995554|Finland|BOLD:AAN1998|LN995554
Leptophlebia marginata|GBMH13134-19|LN995549|Finland|BOLD:AAN1998|LN995549
Leptophlebia marginata|GBMH13133-19|LN995547|Finland|BOLD:AAN1998|LN995547
Leptophlebia marginata|GBMH13140-19|LN995561|Finland|BOLD:AAN1998|LN995561
Leptophlebia marginata|GBMH13135-19|LN995550|Finland|BOLD:AAN1998|LN995550
Leptophlebia marginata|GBMH13132-19|LN995546|Finland|BOLD:AAN1998|LN995546
Leptophlebia marginata|EPHFI033-11|NO-EPH33|Norway|BOLD:AAN1998|JN299100
Leptophlebia marginata|EPHFI032-11|NO-EPH32|Norway|BOLD:AAN1998|JN299099
Leptophlebia marginata|EPHFI031-11|NO-EPH31|Norway|BOLD:AAN1998|JN299098
Leptophlebia marginata|EPHFI092-11|NO-EPH92|Norway|BOLD:AAN1998|JN299152
Habroleptoides confusa|GBA20320-14|HG935080||BOLD:ACB2012|HG935080
Habroleptoides auberti|GBEPT047-13|GBOL01095|Germany|BOLD:ACB2012|KY262277
Habroleptoides confusa|GBA20321-14|HG935081||BOLD:ACB2012|HG935081
Habroleptoides auberti|FBAQU1260-12|BCZSMAQU001070|Germany|BOLD:ACB2012|KY261495
Habroleptoides modesta|GBA20322-14|HG935075||BOLD:ACQ8235|HG935075
Habroleptoides sp. SC2014|GBA22930-15|LN734750|Italy|BOLD:ACS5028|LN734750
Habroleptoides confusa|BGMAY008-10|10BGMAY-008|Bulgaria|BOLD:AAL5733|HM884195
Habroleptoides confusa|BGMAY020-10|10BGMAY-020|Bulgaria|BOLD:AAL5732|HM884207
Habroleptoides confusa|BGMAY024-10|10BGMAY-024|Bulgaria|BOLD:AAL5732|HM884210
Habroleptoides confusa|BGMAY019-10|10BGMAY-019|Bulgaria|BOLD:AAL5732|HM884206
Habroleptoides confusa|BGMAY023-10|10BGMAY-023|Bulgaria|BOLD:AAL5732|HM884209
Habroleptoides confusa|BGMAY018-10|10BGMAY-018|Bulgaria|BOLD:AAL5732|HM884205
Habroleptoides confusa|BGMAY010-10|10BGMAY-010|Bulgaria|BOLD:AAL5732|HM884197
Habroleptoides confusa|BGMAY011-10|10BGMAY-011|Bulgaria|BOLD:AAL5732|HM884198
Habroleptoides confusa|BGMAY012-10|10BGMAY-012|Bulgaria|BOLD:AAL5732|HM884199
Habroleptoides confusa|BGMAY025-10|10BGMAY-025|Bulgaria|BOLD:AAL5732|HM884211
Habroleptoides confusa|BGMAY017-10|10BGMAY-017|Bulgaria|BOLD:AAL5732|HM884204

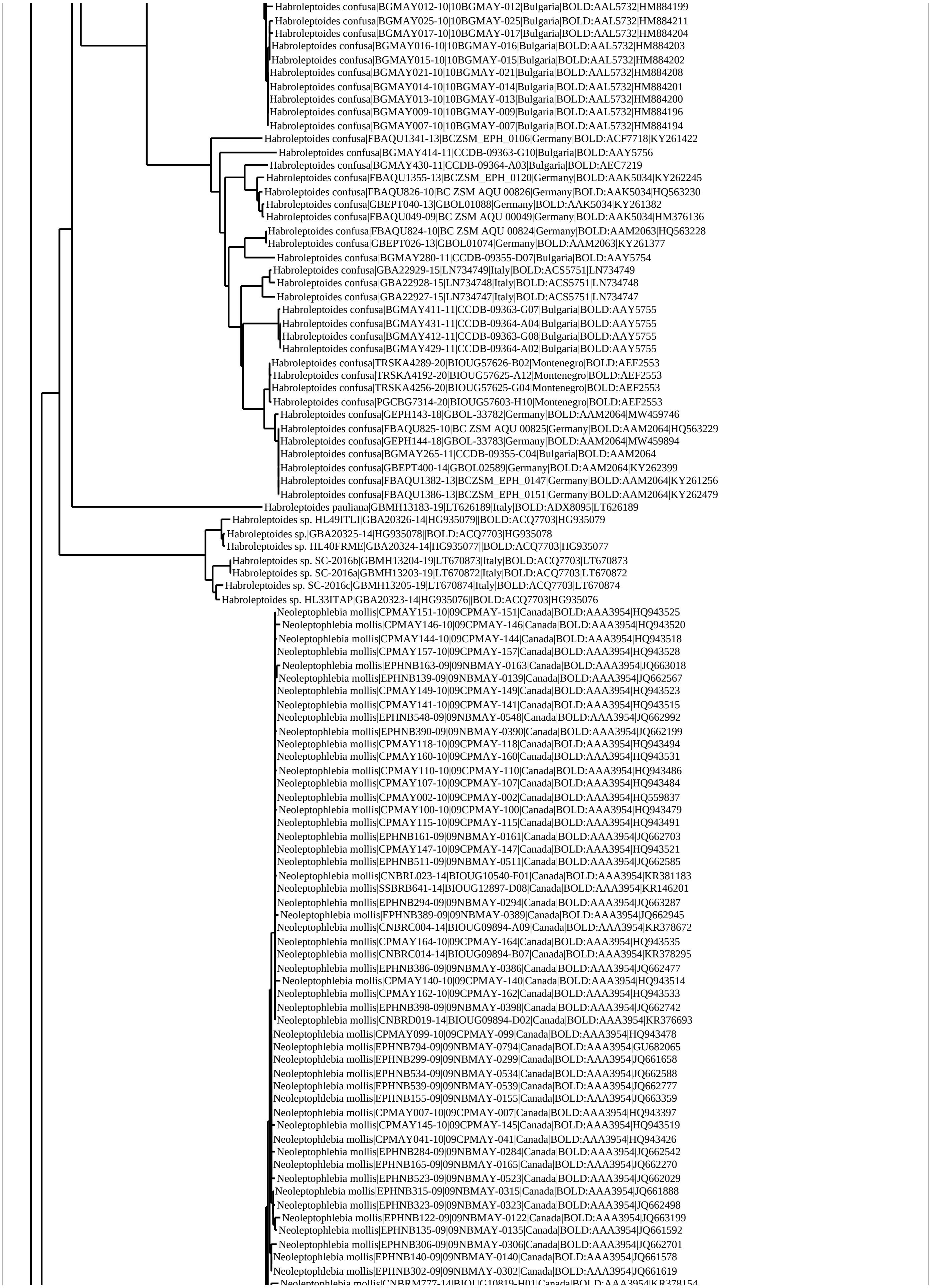
Habroleptoides confusa|BGMAY012-10|10BGMAY-012|Bulgaria|BOLD:AAL5732|HM884199
Habroleptoides confusa|BGMAY025-10|10BGMAY-025|Bulgaria|BOLD:AAL5732|HM884211
Habroleptoides confusa|BGMAY017-10|10BGMAY-017|Bulgaria|BOLD:AAL5732|HM884204
Habroleptoides confusa|BGMAY016-10|10BGMAY-016|Bulgaria|BOLD:AAL5732|HM884203
Habroleptoides confusa|BGMAY015-10|10BGMAY-015|Bulgaria|BOLD:AAL5732|HM884202
Habroleptoides confusa|BGMAY021-10|10BGMAY-021|Bulgaria|BOLD:AAL5732|HM884208
Habroleptoides confusa|BGMAY014-10|10BGMAY-014|Bulgaria|BOLD:AAL5732|HM884201
Habroleptoides confusa|BGMAY013-10|10BGMAY-013|Bulgaria|BOLD:AAL5732|HM884200
Habroleptoides confusa|BGMAY009-10|10BGMAY-009|Bulgaria|BOLD:AAL5732|HM884196
Habroleptoides confusa|BGMAY007-10|10BGMAY-007|Bulgaria|BOLD:AAL5732|HM884194
Habroleptoides confusa|FBAQU1341-13|BCZSM_EPH_0106|Germany|BOLD:ACF7718|KY261422
Habroleptoides confusa|BGMAY414-11|CCDB-09363-G10|Bulgaria|BOLD:AAY5756
Habroleptoides confusa|BGMAY430-11|CCDB-09364-A03|Bulgaria|BOLD:AEC7219
Habroleptoides confusa|FBAQU1355-13|BCZSM_EPH_0120|Germany|BOLD:AAK5034|KY262245
Habroleptoides confusa|FBAQU826-10|BC ZSM AQU 00826|Germany|BOLD:AAK5034|HQ563230
Habroleptoides confusa|GBEPT040-13|GBOL01088|Germany|BOLD:AAK5034|KY261382
Habroleptoides confusa|FBAQU049-09|BC ZSM AQU 00049|Germany|BOLD:AAK5034|HM376136
Habroleptoides confusa|FBAQU824-10|BC ZSM AQU 00824|Germany|BOLD:AAM2063|HQ563228
Habroleptoides confusa|GBEPT026-13|GBOL01074|Germany|BOLD:AAM2063|KY261377
Habroleptoides confusa|BGMAY280-11|CCDB-09355-D07|Bulgaria|BOLD:AAY5754
Habroleptoides confusa|GBA22929-15|LN734749|Italy|BOLD:ACS5751|LN734749
Habroleptoides confusa|GBA22928-15|LN734748|Italy|BOLD:ACS5751|LN734748
Habroleptoides confusa|GBA22927-15|LN734747|Italy|BOLD:ACS5751|LN734747
Habroleptoides confusa|BGMAY411-11|CCDB-09363-G07|Bulgaria|BOLD:AAY5755
Habroleptoides confusa|BGMAY431-11|CCDB-09364-A04|Bulgaria|BOLD:AAY5755
Habroleptoides confusa|BGMAY412-11|CCDB-09363-G08|Bulgaria|BOLD:AAY5755
Habroleptoides confusa|BGMAY429-11|CCDB-09364-A02|Bulgaria|BOLD:AAY5755
Habroleptoides confusa|TRSKA4289-20|BIOUG57626-B02|Montenegro|BOLD:AEF2553
Habroleptoides confusa|TRSKA4192-20|BIOUG57625-A12|Montenegro|BOLD:AEF2553
Habroleptoides confusa|TRSKA4256-20|BIOUG57625-G04|Montenegro|BOLD:AEF2553
Habroleptoides confusa|PGCBG7314-20|BIOUG57603-H10|Montenegro|BOLD:AEF2553
Habroleptoides confusa|GEPH143-18|GBOL-33782|Germany|BOLD:AAM2064|MW459746
Habroleptoides confusa|FBAQU825-10|BC ZSM AQU 00825|Germany|BOLD:AAM2064|HQ563229
Habroleptoides confusa|GEPH144-18|GBOL-33783|Germany|BOLD:AAM2064|MW459894
Habroleptoides confusa|BGMAY265-11|CCDB-09355-C04|Bulgaria|BOLD:AAM2064
Habroleptoides confusa|GBEPT400-14|GBOL02589|Germany|BOLD:AAM2064|KY262399
Habroleptoides confusa|FBAQU1382-13|BCZSM_EPH_0147|Germany|BOLD:AAM2064|KY261256
Habroleptoides confusa|FBAQU1386-13|BCZSM_EPH_0151|Germany|BOLD:AAM2064|KY262479
Habroleptoides pauliana|GBMH13183-19|LT626189|Italy|BOLD:ADX8095|LT626189
Habroleptoides sp. HL49ITLI|GBA20326-14|HG935079||BOLD:ACQ7703|HG935079
Habroleptoides sp.|GBA20325-14|HG935078||BOLD:ACQ7703|HG935078
Habroleptoides sp. HL40FRME|GBA20324-14|HG935077||BOLD:ACQ7703|HG935077
Habroleptoides sp. SC-2016b|GBMH13204-19|LT670873|Italy|BOLD:ACQ7703|LT670873
Habroleptoides sp. SC-2016a|GBMH13203-19|LT670872|Italy|BOLD:ACQ7703|LT670872
Habroleptoides sp. SC-2016c|GBMH13205-19|LT670874|Italy|BOLD:ACQ7703|LT670874
Habroleptoides sp. HL33ITAP|GBA20323-14|HG935076||BOLD:ACQ7703|HG935076
Neoleptophlebia mollis|CPMAY151-10|09CPMAY-151|Canada|BOLD:AAA3954|HQ943525
Neoleptophlebia mollis|CPMAY146-10|09CPMAY-146|Canada|BOLD:AAA3954|HQ943520
Neoleptophlebia mollis|CPMAY144-10|09CPMAY-144|Canada|BOLD:AAA3954|HQ943518
Neoleptophlebia mollis|CPMAY157-10|09CPMAY-157|Canada|BOLD:AAA3954|HQ943528
Neoleptophlebia mollis|EPHNB163-09|09NBMAY-0163|Canada|BOLD:AAA3954|JQ663018
Neoleptophlebia mollis|EPHNB139-09|09NBMAY-0139|Canada|BOLD:AAA3954|JQ662567
Neoleptophlebia mollis|CPMAY149-10|09CPMAY-149|Canada|BOLD:AAA3954|HQ943523
Neoleptophlebia mollis|CPMAY141-10|09CPMAY-141|Canada|BOLD:AAA3954|HQ943515
Neoleptophlebia mollis|EPHNB548-09|09NBMAY-0548|Canada|BOLD:AAA3954|JQ662992
Neoleptophlebia mollis|EPHNB390-09|09NBMAY-0390|Canada|BOLD:AAA3954|JQ662199
Neoleptophlebia mollis|CPMAY118-10|09CPMAY-118|Canada|BOLD:AAA3954|HQ943494
Neoleptophlebia mollis|CPMAY160-10|09CPMAY-160|Canada|BOLD:AAA3954|HQ943531
Neoleptophlebia mollis|CPMAY110-10|09CPMAY-110|Canada|BOLD:AAA3954|HQ943486
Neoleptophlebia mollis|CPMAY107-10|09CPMAY-107|Canada|BOLD:AAA3954|HQ943484
Neoleptophlebia mollis|CPMAY002-10|09CPMAY-002|Canada|BOLD:AAA3954|HQ559837
Neoleptophlebia mollis|CPMAY100-10|09CPMAY-100|Canada|BOLD:AAA3954|HQ943479
Neoleptophlebia mollis|CPMAY115-10|09CPMAY-115|Canada|BOLD:AAA3954|HQ943491
Neoleptophlebia mollis|EPHNB161-09|09NBMAY-0161|Canada|BOLD:AAA3954|JQ662703
Neoleptophlebia mollis|CPMAY147-10|09CPMAY-147|Canada|BOLD:AAA3954|HQ943521
Neoleptophlebia mollis|EPHNB511-09|09NBMAY-0511|Canada|BOLD:AAA3954|JQ662585
Neoleptophlebia mollis|CNBRL023-14|BIOUG10540-F01|Canada|BOLD:AAA3954|KR381183
Neoleptophlebia mollis|SSBRB641-14|BIOUG12897-D08|Canada|BOLD:AAA3954|KR146201
Neoleptophlebia mollis|EPHNB294-09|09NBMAY-0294|Canada|BOLD:AAA3954|JQ663287
Neoleptophlebia mollis|EPHNB389-09|09NBMAY-0389|Canada|BOLD:AAA3954|JQ662945
Neoleptophlebia mollis|CNBRC004-14|BIOUG09894-A09|Canada|BOLD:AAA3954|KR378672
Neoleptophlebia mollis|CPMAY164-10|09CPMAY-164|Canada|BOLD:AAA3954|HQ943535
Neoleptophlebia mollis|CNBRC014-14|BIOUG09894-B07|Canada|BOLD:AAA3954|KR378295
Neoleptophlebia mollis|EPHNB386-09|09NBMAY-0386|Canada|BOLD:AAA3954|JQ662477
Neoleptophlebia mollis|CPMAY140-10|09CPMAY-140|Canada|BOLD:AAA3954|HQ943514
Neoleptophlebia mollis|CPMAY162-10|09CPMAY-162|Canada|BOLD:AAA3954|HQ943533
Neoleptophlebia mollis|EPHNB398-09|09NBMAY-0398|Canada|BOLD:AAA3954|JQ662742
Neoleptophlebia mollis|CNBRD019-14|BIOUG09894-D02|Canada|BOLD:AAA3954|KR376693
Neoleptophlebia mollis|CPMAY099-10|09CPMAY-099|Canada|BOLD:AAA3954|HQ943478
Neoleptophlebia mollis|EPHNB794-09|09NBMAY-0794|Canada|BOLD:AAA3954|GU682065
Neoleptophlebia mollis|EPHNB299-09|09NBMAY-0299|Canada|BOLD:AAA3954|JQ661658
Neoleptophlebia mollis|EPHNB534-09|09NBMAY-0534|Canada|BOLD:AAA3954|JQ662588
Neoleptophlebia mollis|EPHNB539-09|09NBMAY-0539|Canada|BOLD:AAA3954|JQ662777
Neoleptophlebia mollis|EPHNB155-09|09NBMAY-0155|Canada|BOLD:AAA3954|JQ663359
Neoleptophlebia mollis|CPMAY007-10|09CPMAY-007|Canada|BOLD:AAA3954|HQ943397
Neoleptophlebia mollis|CPMAY145-10|09CPMAY-145|Canada|BOLD:AAA3954|HQ943519
Neoleptophlebia mollis|CPMAY041-10|09CPMAY-041|Canada|BOLD:AAA3954|HQ943426
Neoleptophlebia mollis|EPHNB284-09|09NBMAY-0284|Canada|BOLD:AAA3954|JQ662542
Neoleptophlebia mollis|EPHNB165-09|09NBMAY-0165|Canada|BOLD:AAA3954|JQ662270
Neoleptophlebia mollis|EPHNB523-09|09NBMAY-0523|Canada|BOLD:AAA3954|JQ662029
Neoleptophlebia mollis|EPHNB315-09|09NBMAY-0315|Canada|BOLD:AAA3954|JQ661888
Neoleptophlebia mollis|EPHNB323-09|09NBMAY-0323|Canada|BOLD:AAA3954|JQ662498
Neoleptophlebia mollis|EPHNB122-09|09NBMAY-0122|Canada|BOLD:AAA3954|JQ663199
Neoleptophlebia mollis|EPHNB135-09|09NBMAY-0135|Canada|BOLD:AAA3954|JQ661592
Neoleptophlebia mollis|EPHNB306-09|09NBMAY-0306|Canada|BOLD:AAA3954|JQ662701
Neoleptophlebia mollis|EPHNB140-09|09NBMAY-0140|Canada|BOLD:AAA3954|JQ661578
Neoleptophlebia mollis|EPHNB302-09|09NBMAY-0302|Canada|BOLD:AAA3954|JQ661619
Neoleptophlebia mollis|CNBRM777-14|BIOUG10819-H01|Canada|BOLD:AAA3954|KR378154

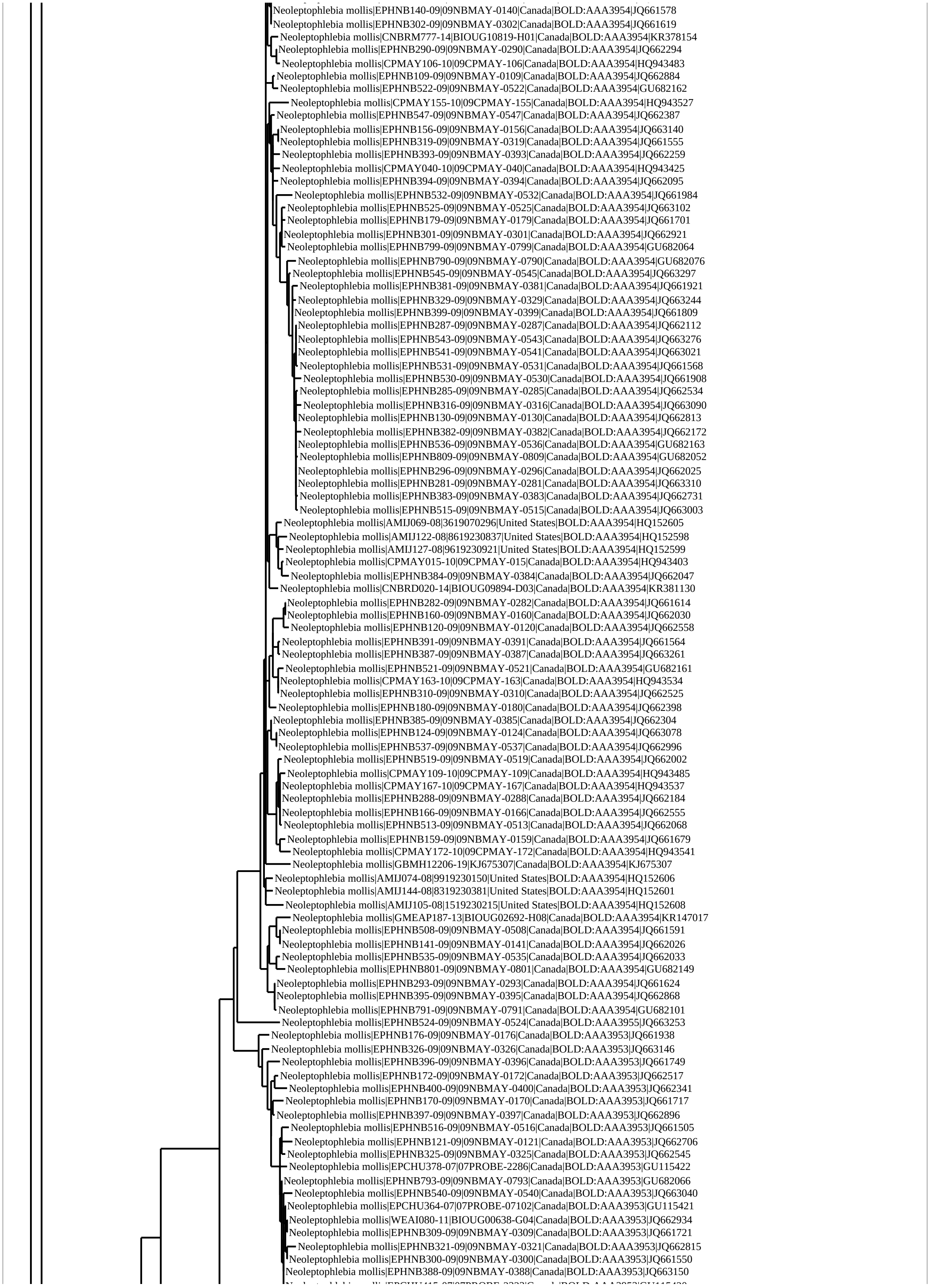
Neoleptophlebia mollis|EPHNB140-09|09NBMAY-0140|Canada|BOLD:AAA3954|JQ661578
Neoleptophlebia mollis|EPHNB302-09|09NBMAY-0302|Canada|BOLD:AAA3954|JQ661619
Neoleptophlebia mollis|CNBRM777-14|BIOUG10819-H01|Canada|BOLD:AAA3954|KR378154
Neoleptophlebia mollis|EPHNB290-09|09NBMAY-0290|Canada|BOLD:AAA3954|JQ662294
Neoleptophlebia mollis|CPMAY106-10|09CPMAY-106|Canada|BOLD:AAA3954|HQ943483
Neoleptophlebia mollis|EPHNB109-09|09NBMAY-0109|Canada|BOLD:AAA3954|JQ662884
Neoleptophlebia mollis|EPHNB522-09|09NBMAY-0522|Canada|BOLD:AAA3954|GU682162
Neoleptophlebia mollis|CPMAY155-10|09CPMAY-155|Canada|BOLD:AAA3954|HQ943527
Neoleptophlebia mollis|EPHNB547-09|09NBMAY-0547|Canada|BOLD:AAA3954|JQ662387
Neoleptophlebia mollis|EPHNB156-09|09NBMAY-0156|Canada|BOLD:AAA3954|JQ663140
Neoleptophlebia mollis|EPHNB319-09|09NBMAY-0319|Canada|BOLD:AAA3954|JQ661555
Neoleptophlebia mollis|EPHNB393-09|09NBMAY-0393|Canada|BOLD:AAA3954|JQ662259
Neoleptophlebia mollis|CPMAY040-10|09CPMAY-040|Canada|BOLD:AAA3954|HQ943425
Neoleptophlebia mollis|EPHNB394-09|09NBMAY-0394|Canada|BOLD:AAA3954|JQ662095
Neoleptophlebia mollis|EPHNB532-09|09NBMAY-0532|Canada|BOLD:AAA3954|JQ661984
Neoleptophlebia mollis|EPHNB525-09|09NBMAY-0525|Canada|BOLD:AAA3954|JQ663102
Neoleptophlebia mollis|EPHNB179-09|09NBMAY-0179|Canada|BOLD:AAA3954|JQ661701
Neoleptophlebia mollis|EPHNB301-09|09NBMAY-0301|Canada|BOLD:AAA3954|JQ662921
Neoleptophlebia mollis|EPHNB799-09|09NBMAY-0799|Canada|BOLD:AAA3954|GU682064
Neoleptophlebia mollis|EPHNB790-09|09NBMAY-0790|Canada|BOLD:AAA3954|GU682076
Neoleptophlebia mollis|EPHNB545-09|09NBMAY-0545|Canada|BOLD:AAA3954|JQ663297
Neoleptophlebia mollis|EPHNB381-09|09NBMAY-0381|Canada|BOLD:AAA3954|JQ661921
Neoleptophlebia mollis|EPHNB329-09|09NBMAY-0329|Canada|BOLD:AAA3954|JQ663244
Neoleptophlebia mollis|EPHNB399-09|09NBMAY-0399|Canada|BOLD:AAA3954|JQ661809
Neoleptophlebia mollis|EPHNB287-09|09NBMAY-0287|Canada|BOLD:AAA3954|JQ662112
Neoleptophlebia mollis|EPHNB543-09|09NBMAY-0543|Canada|BOLD:AAA3954|JQ663276
Neoleptophlebia mollis|EPHNB541-09|09NBMAY-0541|Canada|BOLD:AAA3954|JQ663021
Neoleptophlebia mollis|EPHNB531-09|09NBMAY-0531|Canada|BOLD:AAA3954|JQ661568
Neoleptophlebia mollis|EPHNB530-09|09NBMAY-0530|Canada|BOLD:AAA3954|JQ661908
Neoleptophlebia mollis|EPHNB285-09|09NBMAY-0285|Canada|BOLD:AAA3954|JQ662534
Neoleptophlebia mollis|EPHNB316-09|09NBMAY-0316|Canada|BOLD:AAA3954|JQ663090
Neoleptophlebia mollis|EPHNB130-09|09NBMAY-0130|Canada|BOLD:AAA3954|JQ662813
Neoleptophlebia mollis|EPHNB382-09|09NBMAY-0382|Canada|BOLD:AAA3954|JQ662172
Neoleptophlebia mollis|EPHNB536-09|09NBMAY-0536|Canada|BOLD:AAA3954|GU682163
Neoleptophlebia mollis|EPHNB809-09|09NBMAY-0809|Canada|BOLD:AAA3954|GU682052
Neoleptophlebia mollis|EPHNB296-09|09NBMAY-0296|Canada|BOLD:AAA3954|JQ662025
Neoleptophlebia mollis|EPHNB281-09|09NBMAY-0281|Canada|BOLD:AAA3954|JQ663310
Neoleptophlebia mollis|EPHNB383-09|09NBMAY-0383|Canada|BOLD:AAA3954|JQ662731
Neoleptophlebia mollis|EPHNB515-09|09NBMAY-0515|Canada|BOLD:AAA3954|JQ663003
Neoleptophlebia mollis|AMIJ069-08|3619070296|United States|BOLD:AAA3954|HQ152605
Neoleptophlebia mollis|AMIJ122-08|8619230837|United States|BOLD:AAA3954|HQ152598
Neoleptophlebia mollis|AMIJ127-08|9619230921|United States|BOLD:AAA3954|HQ152599
Neoleptophlebia mollis|CPMAY015-10|09CPMAY-015|Canada|BOLD:AAA3954|HQ943403
Neoleptophlebia mollis|EPHNB384-09|09NBMAY-0384|Canada|BOLD:AAA3954|JQ662047
Neoleptophlebia mollis|CNBRD020-14|BIOUG09894-D03|Canada|BOLD:AAA3954|KR381130
Neoleptophlebia mollis|EPHNB282-09|09NBMAY-0282|Canada|BOLD:AAA3954|JQ661614
Neoleptophlebia mollis|EPHNB160-09|09NBMAY-0160|Canada|BOLD:AAA3954|JQ662030
Neoleptophlebia mollis|EPHNB120-09|09NBMAY-0120|Canada|BOLD:AAA3954|JQ662558
Neoleptophlebia mollis|EPHNB391-09|09NBMAY-0391|Canada|BOLD:AAA3954|JQ661564
Neoleptophlebia mollis|EPHNB387-09|09NBMAY-0387|Canada|BOLD:AAA3954|JQ663261
Neoleptophlebia mollis|EPHNB521-09|09NBMAY-0521|Canada|BOLD:AAA3954|GU682161
Neoleptophlebia mollis|CPMAY163-10|09CPMAY-163|Canada|BOLD:AAA3954|HQ943534
Neoleptophlebia mollis|EPHNB310-09|09NBMAY-0310|Canada|BOLD:AAA3954|JQ662525
Neoleptophlebia mollis|EPHNB180-09|09NBMAY-0180|Canada|BOLD:AAA3954|JQ662398
Neoleptophlebia mollis|EPHNB385-09|09NBMAY-0385|Canada|BOLD:AAA3954|JQ662304
Neoleptophlebia mollis|EPHNB124-09|09NBMAY-0124|Canada|BOLD:AAA3954|JQ663078
Neoleptophlebia mollis|EPHNB537-09|09NBMAY-0537|Canada|BOLD:AAA3954|JQ662996
Neoleptophlebia mollis|EPHNB519-09|09NBMAY-0519|Canada|BOLD:AAA3954|JQ662002
Neoleptophlebia mollis|CPMAY109-10|09CPMAY-109|Canada|BOLD:AAA3954|HQ943485
Neoleptophlebia mollis|CPMAY167-10|09CPMAY-167|Canada|BOLD:AAA3954|HQ943537
Neoleptophlebia mollis|EPHNB288-09|09NBMAY-0288|Canada|BOLD:AAA3954|JQ662184
Neoleptophlebia mollis|EPHNB166-09|09NBMAY-0166|Canada|BOLD:AAA3954|JQ662555
Neoleptophlebia mollis|EPHNB513-09|09NBMAY-0513|Canada|BOLD:AAA3954|JQ662068
Neoleptophlebia mollis|EPHNB159-09|09NBMAY-0159|Canada|BOLD:AAA3954|JQ661679
Neoleptophlebia mollis|CPMAY172-10|09CPMAY-172|Canada|BOLD:AAA3954|HQ943541
Neoleptophlebia mollis|GBMH12206-19|KJ675307|Canada|BOLD:AAA3954|KJ675307
Neoleptophlebia mollis|AMIJ074-08|9919230150|United States|BOLD:AAA3954|HQ152606
Neoleptophlebia mollis|AMIJ144-08|8319230381|United States|BOLD:AAA3954|HQ152601
Neoleptophlebia mollis|AMIJ105-08|1519230215|United States|BOLD:AAA3954|HQ152608
Neoleptophlebia mollis|GMEAP187-13|BIOUG02692-H08|Canada|BOLD:AAA3954|KR147017
Neoleptophlebia mollis|EPHNB508-09|09NBMAY-0508|Canada|BOLD:AAA3954|JQ661591
Neoleptophlebia mollis|EPHNB141-09|09NBMAY-0141|Canada|BOLD:AAA3954|JQ662026
Neoleptophlebia mollis|EPHNB535-09|09NBMAY-0535|Canada|BOLD:AAA3954|JQ662033
Neoleptophlebia mollis|EPHNB801-09|09NBMAY-0801|Canada|BOLD:AAA3954|GU682149
Neoleptophlebia mollis|EPHNB293-09|09NBMAY-0293|Canada|BOLD:AAA3954|JQ661624
Neoleptophlebia mollis|EPHNB395-09|09NBMAY-0395|Canada|BOLD:AAA3954|JQ662868
Neoleptophlebia mollis|EPHNB791-09|09NBMAY-0791|Canada|BOLD:AAA3954|GU682101
Neoleptophlebia mollis|EPHNB524-09|09NBMAY-0524|Canada|BOLD:AAA3955|JQ663253
Neoleptophlebia mollis|EPHNB176-09|09NBMAY-0176|Canada|BOLD:AAA3953|JQ661938
Neoleptophlebia mollis|EPHNB326-09|09NBMAY-0326|Canada|BOLD:AAA3953|JQ663146
Neoleptophlebia mollis|EPHNB396-09|09NBMAY-0396|Canada|BOLD:AAA3953|JQ661749
Neoleptophlebia mollis|EPHNB172-09|09NBMAY-0172|Canada|BOLD:AAA3953|JQ662517
Neoleptophlebia mollis|EPHNB400-09|09NBMAY-0400|Canada|BOLD:AAA3953|JQ662341
Neoleptophlebia mollis|EPHNB170-09|09NBMAY-0170|Canada|BOLD:AAA3953|JQ661717
Neoleptophlebia mollis|EPHNB397-09|09NBMAY-0397|Canada|BOLD:AAA3953|JQ662896
Neoleptophlebia mollis|EPHNB516-09|09NBMAY-0516|Canada|BOLD:AAA3953|JQ661505
Neoleptophlebia mollis|EPHNB121-09|09NBMAY-0121|Canada|BOLD:AAA3953|JQ662706
Neoleptophlebia mollis|EPHNB325-09|09NBMAY-0325|Canada|BOLD:AAA3953|JQ662545
Neoleptophlebia mollis|EPCHU378-07|07PROBE-2286|Canada|BOLD:AAA3953|GU115422
Neoleptophlebia mollis|EPHNB793-09|09NBMAY-0793|Canada|BOLD:AAA3953|GU682066
Neoleptophlebia mollis|EPHNB540-09|09NBMAY-0540|Canada|BOLD:AAA3953|JQ663040
Neoleptophlebia mollis|EPCHU364-07|07PROBE-07102|Canada|BOLD:AAA3953|GU115421
Neoleptophlebia mollis|WEAI080-11|BIOUG00638-G04|Canada|BOLD:AAA3953|JQ662934
Neoleptophlebia mollis|EPHNB309-09|09NBMAY-0309|Canada|BOLD:AAA3953|JQ661721
Neoleptophlebia mollis|EPHNB321-09|09NBMAY-0321|Canada|BOLD:AAA3953|JQ662815
Neoleptophlebia mollis|EPHNB300-09|09NBMAY-0300|Canada|BOLD:AAA3953|JQ661550
Neoleptophlebia mollis|EPHNB388-09|09NBMAY-0388|Canada|BOLD:AAA3953|JQ663150

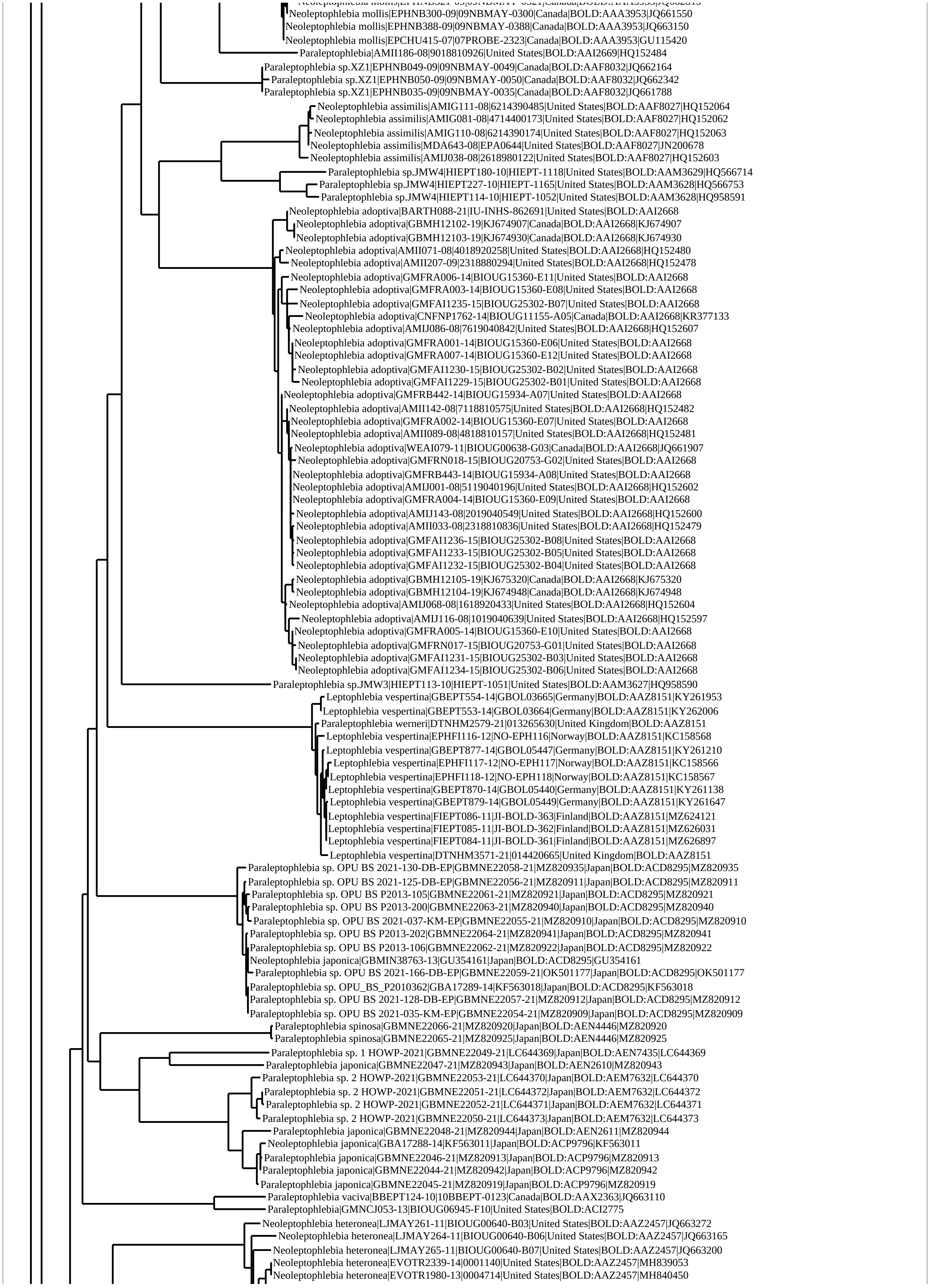
Neoleptophlebia mollis|EPHNB300-09|09NBMAY-0300|Canada|BOLD:AAA3953|JQ661550
Neoleptophlebia mollis|EPHNB388-09|09NBMAY-0388|Canada|BOLD:AAA3953|JQ663150
Neoleptophlebia mollis|EPCHU415-07|07PROBE-2323|Canada|BOLD:AAA3953|GU115420
Paraleptophlebia|AMII186-08|9018810926|United States|BOLD:AAI2669|HQ152484
Paraleptophlebia sp.XZ1|EPHNB049-09|09NBMAY-0049|Canada|BOLD:AAF8032|JQ662164
Paraleptophlebia sp.XZ1|EPHNB050-09|09NBMAY-0050|Canada|BOLD:AAF8032|JQ662342
Paraleptophlebia sp.XZ1|EPHNB035-09|09NBMAY-0035|Canada|BOLD:AAF8032|JQ661788
Neoleptophlebia assimilis|AMIG111-08|6214390485|United States|BOLD:AAF8027|HQ152064
Neoleptophlebia assimilis|AMIG081-08|4714400173|United States|BOLD:AAF8027|HQ152062
Neoleptophlebia assimilis|AMIG110-08|6214390174|United States|BOLD:AAF8027|HQ152063
Neoleptophlebia assimilis|MDA643-08|EPA0644|United States|BOLD:AAF8027|JN200678
Neoleptophlebia assimilis|AMIJ038-08|2618980122|United States|BOLD:AAF8027|HQ152603
Paraleptophlebia sp.JMW4|HIEPT180-10|HIEPT-1118|United States|BOLD:AAM3629|HQ566714
Paraleptophlebia sp.JMW4|HIEPT227-10|HIEPT-1165|United States|BOLD:AAM3628|HQ566753
Paraleptophlebia sp.JMW4|HIEPT114-10|HIEPT-1052|United States|BOLD:AAM3628|HQ958591
Neoleptophlebia adoptiva|BARTH088-21|IU-INHS-862691|United States|BOLD:AAI2668
Neoleptophlebia adoptiva|GBMH12102-19|KJ674907|Canada|BOLD:AAI2668|KJ674907
Neoleptophlebia adoptiva|GBMH12103-19|KJ674930|Canada|BOLD:AAI2668|KJ674930
Neoleptophlebia adoptiva|AMII071-08|4018920258|United States|BOLD:AAI2668|HQ152480
Neoleptophlebia adoptiva|AMII207-09|2318880294|United States|BOLD:AAI2668|HQ152478
Neoleptophlebia adoptiva|GMFRA006-14|BIOUG15360-E11|United States|BOLD:AAI2668
Neoleptophlebia adoptiva|GMFRA003-14|BIOUG15360-E08|United States|BOLD:AAI2668
Neoleptophlebia adoptiva|GMFAI1235-15|BIOUG25302-B07|United States|BOLD:AAI2668
Neoleptophlebia adoptiva|CNFNP1762-14|BIOUG11155-A05|Canada|BOLD:AAI2668|KR377133
Neoleptophlebia adoptiva|AMIJ086-08|7619040842|United States|BOLD:AAI2668|HQ152607
Neoleptophlebia adoptiva|GMFRA001-14|BIOUG15360-E06|United States|BOLD:AAI2668
Neoleptophlebia adoptiva|GMFRA007-14|BIOUG15360-E12|United States|BOLD:AAI2668
Neoleptophlebia adoptiva|GMFAI1230-15|BIOUG25302-B02|United States|BOLD:AAI2668
Neoleptophlebia adoptiva|GMFAI1229-15|BIOUG25302-B01|United States|BOLD:AAI2668
Neoleptophlebia adoptiva|GMFRB442-14|BIOUG15934-A07|United States|BOLD:AAI2668
Neoleptophlebia adoptiva|AMII142-08|7118810575|United States|BOLD:AAI2668|HQ152482
Neoleptophlebia adoptiva|GMFRA002-14|BIOUG15360-E07|United States|BOLD:AAI2668
Neoleptophlebia adoptiva|AMII089-08|4818810157|United States|BOLD:AAI2668|HQ152481
Neoleptophlebia adoptiva|WEAI079-11|BIOUG00638-G03|Canada|BOLD:AAI2668|JQ661907
Neoleptophlebia adoptiva|GMFRN018-15|BIOUG20753-G02|United States|BOLD:AAI2668
Neoleptophlebia adoptiva|GMFRB443-14|BIOUG15934-A08|United States|BOLD:AAI2668
Neoleptophlebia adoptiva|AMIJ001-08|5119040196|United States|BOLD:AAI2668|HQ152602
Neoleptophlebia adoptiva|GMFRA004-14|BIOUG15360-E09|United States|BOLD:AAI2668
Neoleptophlebia adoptiva|AMIJ143-08|2019040549|United States|BOLD:AAI2668|HQ152600
Neoleptophlebia adoptiva|AMII033-08|2318810836|United States|BOLD:AAI2668|HQ152479
Neoleptophlebia adoptiva|GMFAI1236-15|BIOUG25302-B08|United States|BOLD:AAI2668
Neoleptophlebia adoptiva|GMFAI1233-15|BIOUG25302-B05|United States|BOLD:AAI2668
Neoleptophlebia adoptiva|GMFAI1232-15|BIOUG25302-B04|United States|BOLD:AAI2668
Neoleptophlebia adoptiva|GBMH12105-19|KJ675320|Canada|BOLD:AAI2668|KJ675320
Neoleptophlebia adoptiva|GBMH12104-19|KJ674948|Canada|BOLD:AAI2668|KJ674948
Neoleptophlebia adoptiva|AMIJ068-08|1618920433|United States|BOLD:AAI2668|HQ152604
Neoleptophlebia adoptiva|AMIJ116-08|1019040639|United States|BOLD:AAI2668|HQ152597
Neoleptophlebia adoptiva|GMFRA005-14|BIOUG15360-E10|United States|BOLD:AAI2668
Neoleptophlebia adoptiva|GMFRN017-15|BIOUG20753-G01|United States|BOLD:AAI2668
Neoleptophlebia adoptiva|GMFAI1231-15|BIOUG25302-B03|United States|BOLD:AAI2668
Neoleptophlebia adoptiva|GMFAI1234-15|BIOUG25302-B06|United States|BOLD:AAI2668
Paraleptophlebia sp.JMW3|HIEPT113-10|HIEPT-1051|United States|BOLD:AAM3627|HQ958590
Leptophlebia vespertina|GBEPT554-14|GBOL03665|Germany|BOLD:AAZ8151|KY261953
Leptophlebia vespertina|GBEPT553-14|GBOL03664|Germany|BOLD:AAZ8151|KY262006
Paraleptophlebia werneri|DTNHM2579-21|013265630|United Kingdom|BOLD:AAZ8151
Leptophlebia vespertina|EPHFI116-12|NO-EPH116|Norway|BOLD:AAZ8151|KC158568
Leptophlebia vespertina|GBEPT877-14|GBOL05447|Germany|BOLD:AAZ8151|KY261210
Leptophlebia vespertina|EPHFI117-12|NO-EPH117|Norway|BOLD:AAZ8151|KC158566
Leptophlebia vespertina|EPHFI118-12|NO-EPH118|Norway|BOLD:AAZ8151|KC158567
Leptophlebia vespertina|GBEPT870-14|GBOL05440|Germany|BOLD:AAZ8151|KY261138
Leptophlebia vespertina|GBEPT879-14|GBOL05449|Germany|BOLD:AAZ8151|KY261647
Leptophlebia vespertina|FIEPT086-11|JI-BOLD-363|Finland|BOLD:AAZ8151|MZ624121
Leptophlebia vespertina|FIEPT085-11|JI-BOLD-362|Finland|BOLD:AAZ8151|MZ626031
Leptophlebia vespertina|FIEPT084-11|JI-BOLD-361|Finland|BOLD:AAZ8151|MZ626897
Leptophlebia vespertina|DTNHM3571-21|014420665|United Kingdom|BOLD:AAZ8151
Paraleptophlebia sp. OPU BS 2021-130-DB-EP|GBMNE22058-21|MZ820935|Japan|BOLD:ACD8295|MZ820935
Paraleptophlebia sp. OPU BS 2021-125-DB-EP|GBMNE22056-21|MZ820911|Japan|BOLD:ACD8295|MZ820911
Paraleptophlebia sp. OPU BS P2013-105|GBMNE22061-21|MZ820921|Japan|BOLD:ACD8295|MZ820921
Paraleptophlebia sp. OPU BS P2013-200|GBMNE22063-21|MZ820940|Japan|BOLD:ACD8295|MZ820940
Paraleptophlebia sp. OPU BS 2021-037-KM-EP|GBMNE22055-21|MZ820910|Japan|BOLD:ACD8295|MZ820910
Paraleptophlebia sp. OPU BS P2013-202|GBMNE22064-21|MZ820941|Japan|BOLD:ACD8295|MZ820941
Paraleptophlebia sp. OPU BS P2013-106|GBMNE22062-21|MZ820922|Japan|BOLD:ACD8295|MZ820922
Neoleptophlebia japonica|GBMIN38763-13|GU354161|Japan|BOLD:ACD8295|GU354161
Paraleptophlebia sp. OPU BS 2021-166-DB-EP|GBMNE22059-21|OK501177|Japan|BOLD:ACD8295|OK501177
Paraleptophlebia sp. OPU_BS_P2010362|GBA17289-14|KF563018|Japan|BOLD:ACD8295|KF563018
Paraleptophlebia sp. OPU BS 2021-128-DB-EP|GBMNE22057-21|MZ820912|Japan|BOLD:ACD8295|MZ820912
Paraleptophlebia sp. OPU BS 2021-035-KM-EP|GBMNE22054-21|MZ820909|Japan|BOLD:ACD8295|MZ820909
Paraleptophlebia spinosa|GBMNE22066-21|MZ820920|Japan|BOLD:AEN4446|MZ820920
Paraleptophlebia spinosa|GBMNE22065-21|MZ820925|Japan|BOLD:AEN4446|MZ820925
Paraleptophlebia sp. 1 HOWP-2021|GBMNE22049-21|LC644369|Japan|BOLD:AEN7435|LC644369
Paraleptophlebia japonica|GBMNE22047-21|MZ820943|Japan|BOLD:AEN2610|MZ820943
Paraleptophlebia sp. 2 HOWP-2021|GBMNE22053-21|LC644370|Japan|BOLD:AEM7632|LC644370
Paraleptophlebia sp. 2 HOWP-2021|GBMNE22051-21|LC644372|Japan|BOLD:AEM7632|LC644372
Paraleptophlebia sp. 2 HOWP-2021|GBMNE22052-21|LC644371|Japan|BOLD:AEM7632|LC644371
Paraleptophlebia sp. 2 HOWP-2021|GBMNE22050-21|LC644373|Japan|BOLD:AEM7632|LC644373
Paraleptophlebia japonica|GBMNE22048-21|MZ820944|Japan|BOLD:AEN2611|MZ820944
Neoleptophlebia japonica|GBA17288-14|KF563011|Japan|BOLD:ACP9796|KF563011
Paraleptophlebia japonica|GBMNE22046-21|MZ820913|Japan|BOLD:ACP9796|MZ820913
Paraleptophlebia japonica|GBMNE22044-21|MZ820942|Japan|BOLD:ACP9796|MZ820942
Paraleptophlebia japonica|GBMNE22045-21|MZ820919|Japan|BOLD:ACP9796|MZ820919
Paraleptophlebia vaciva|BBEPT124-10|10BBEPT-0123|Canada|BOLD:AAX2363|JQ663110
Paraleptophlebia|GMNCJ053-13|BIOUG06945-F10|United States|BOLD:ACI2775
Neoleptophlebia heteronea|LJMAY261-11|BIOUG00640-B03|United States|BOLD:AAZ2457|JQ663272
Neoleptophlebia heteronea|LJMAY264-11|BIOUG00640-B06|United States|BOLD:AAZ2457|JQ663165
Neoleptophlebia heteronea|LJMAY265-11|BIOUG00640-B07|United States|BOLD:AAZ2457|JQ663200
Neoleptophlebia heteronea|EVOTR2339-14|0001140|United States|BOLD:AAZ2457|MH839053
Neoleptophlebia heteronea|EVOTR1980-13|0004714|United States|BOLD:AAZ2457|MH840450

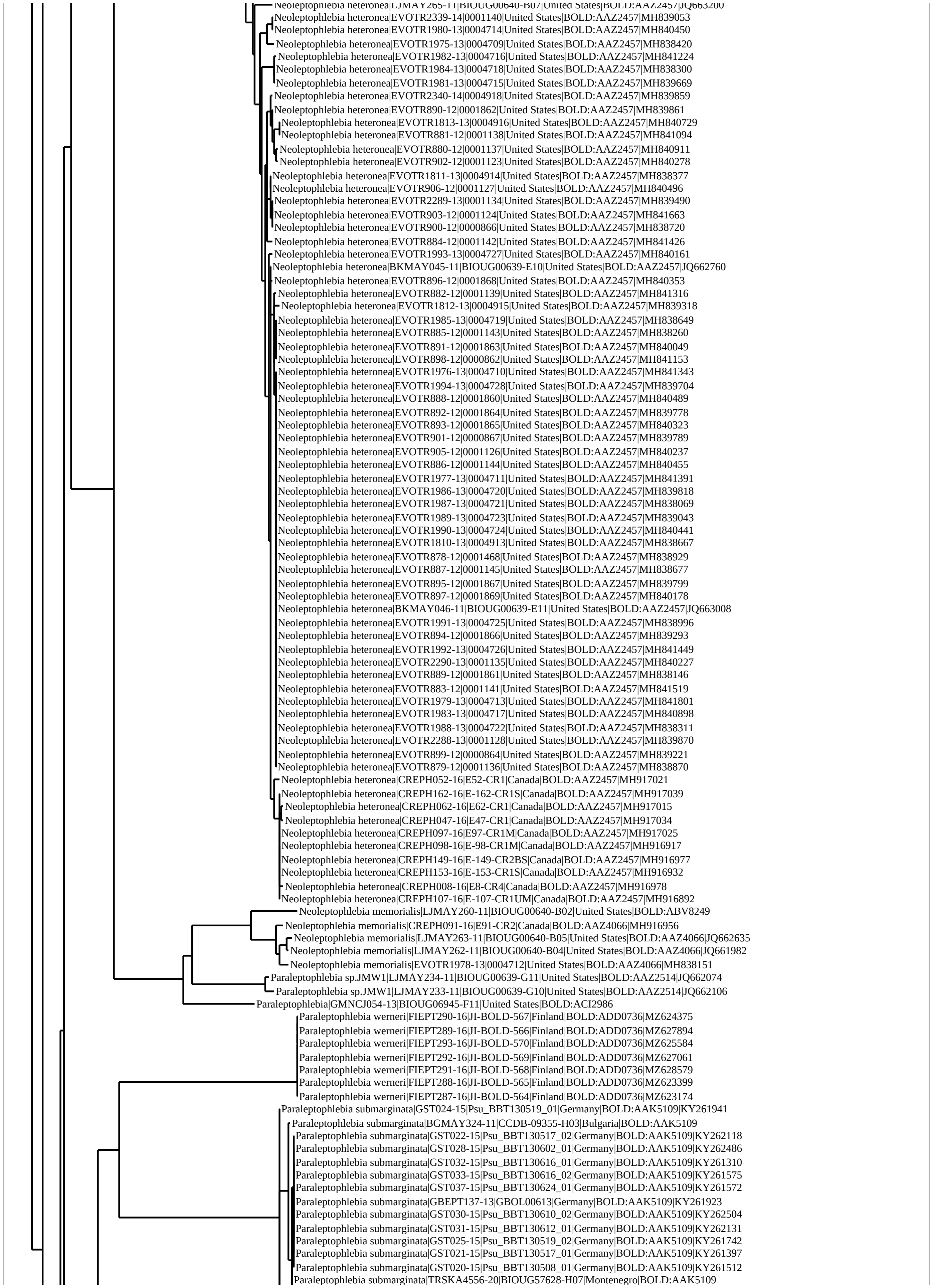
Neoleptophlebia heteronea|LJMAY265-11|BIOUG00640-B07|United States|BOLD:AAZ2457|JQ663200
Neoleptophlebia heteronea|EVOTR2339-14|0001140|United States|BOLD:AAZ2457|MH839053
Neoleptophlebia heteronea|EVOTR1980-13|0004714|United States|BOLD:AAZ2457|MH840450
Neoleptophlebia heteronea|EVOTR1975-13|0004709|United States|BOLD:AAZ2457|MH838420
Neoleptophlebia heteronea|EVOTR1982-13|0004716|United States|BOLD:AAZ2457|MH841224
Neoleptophlebia heteronea|EVOTR1984-13|0004718|United States|BOLD:AAZ2457|MH838300
Neoleptophlebia heteronea|EVOTR1981-13|0004715|United States|BOLD:AAZ2457|MH839669
Neoleptophlebia heteronea|EVOTR2340-14|0004918|United States|BOLD:AAZ2457|MH839859
Neoleptophlebia heteronea|EVOTR890-12|0001862|United States|BOLD:AAZ2457|MH839861
Neoleptophlebia heteronea|EVOTR1813-13|0004916|United States|BOLD:AAZ2457|MH840729
Neoleptophlebia heteronea|EVOTR881-12|0001138|United States|BOLD:AAZ2457|MH841094
Neoleptophlebia heteronea|EVOTR880-12|0001137|United States|BOLD:AAZ2457|MH840911
Neoleptophlebia heteronea|EVOTR902-12|0001123|United States|BOLD:AAZ2457|MH840278
Neoleptophlebia heteronea|EVOTR1811-13|0004914|United States|BOLD:AAZ2457|MH838377
Neoleptophlebia heteronea|EVOTR906-12|0001127|United States|BOLD:AAZ2457|MH840496
Neoleptophlebia heteronea|EVOTR2289-13|0001134|United States|BOLD:AAZ2457|MH839490
Neoleptophlebia heteronea|EVOTR903-12|0001124|United States|BOLD:AAZ2457|MH841663
Neoleptophlebia heteronea|EVOTR900-12|0000866|United States|BOLD:AAZ2457|MH838720
Neoleptophlebia heteronea|EVOTR884-12|0001142|United States|BOLD:AAZ2457|MH841426
Neoleptophlebia heteronea|EVOTR1993-13|0004727|United States|BOLD:AAZ2457|MH840161
Neoleptophlebia heteronea|BKMAY045-11|BIOUG00639-E10|United States|BOLD:AAZ2457|JQ662760
Neoleptophlebia heteronea|EVOTR896-12|0001868|United States|BOLD:AAZ2457|MH840353
Neoleptophlebia heteronea|EVOTR882-12|0001139|United States|BOLD:AAZ2457|MH841316
Neoleptophlebia heteronea|EVOTR1812-13|0004915|United States|BOLD:AAZ2457|MH839318
Neoleptophlebia heteronea|EVOTR1985-13|0004719|United States|BOLD:AAZ2457|MH838649
Neoleptophlebia heteronea|EVOTR885-12|0001143|United States|BOLD:AAZ2457|MH838260
Neoleptophlebia heteronea|EVOTR891-12|0001863|United States|BOLD:AAZ2457|MH840049
Neoleptophlebia heteronea|EVOTR898-12|0000862|United States|BOLD:AAZ2457|MH841153
Neoleptophlebia heteronea|EVOTR1976-13|0004710|United States|BOLD:AAZ2457|MH841343
Neoleptophlebia heteronea|EVOTR1994-13|0004728|United States|BOLD:AAZ2457|MH839704
Neoleptophlebia heteronea|EVOTR888-12|0001860|United States|BOLD:AAZ2457|MH840489
Neoleptophlebia heteronea|EVOTR892-12|0001864|United States|BOLD:AAZ2457|MH839778
Neoleptophlebia heteronea|EVOTR893-12|0001865|United States|BOLD:AAZ2457|MH840323
Neoleptophlebia heteronea|EVOTR901-12|0000867|United States|BOLD:AAZ2457|MH839789
Neoleptophlebia heteronea|EVOTR905-12|0001126|United States|BOLD:AAZ2457|MH840237
Neoleptophlebia heteronea|EVOTR886-12|0001144|United States|BOLD:AAZ2457|MH840455
Neoleptophlebia heteronea|EVOTR1977-13|0004711|United States|BOLD:AAZ2457|MH841391
Neoleptophlebia heteronea|EVOTR1986-13|0004720|United States|BOLD:AAZ2457|MH839818
Neoleptophlebia heteronea|EVOTR1987-13|0004721|United States|BOLD:AAZ2457|MH838069
Neoleptophlebia heteronea|EVOTR1989-13|0004723|United States|BOLD:AAZ2457|MH839043
Neoleptophlebia heteronea|EVOTR1990-13|0004724|United States|BOLD:AAZ2457|MH840441
Neoleptophlebia heteronea|EVOTR1810-13|0004913|United States|BOLD:AAZ2457|MH838667
Neoleptophlebia heteronea|EVOTR878-12|0001468|United States|BOLD:AAZ2457|MH838929
Neoleptophlebia heteronea|EVOTR887-12|0001145|United States|BOLD:AAZ2457|MH838677
Neoleptophlebia heteronea|EVOTR895-12|0001867|United States|BOLD:AAZ2457|MH839799
Neoleptophlebia heteronea|EVOTR897-12|0001869|United States|BOLD:AAZ2457|MH840178
Neoleptophlebia heteronea|BKMAY046-11|BIOUG00639-E11|United States|BOLD:AAZ2457|JQ663008
Neoleptophlebia heteronea|EVOTR1991-13|0004725|United States|BOLD:AAZ2457|MH838996
Neoleptophlebia heteronea|EVOTR894-12|0001866|United States|BOLD:AAZ2457|MH839293
Neoleptophlebia heteronea|EVOTR1992-13|0004726|United States|BOLD:AAZ2457|MH841449
Neoleptophlebia heteronea|EVOTR2290-13|0001135|United States|BOLD:AAZ2457|MH840227
Neoleptophlebia heteronea|EVOTR889-12|0001861|United States|BOLD:AAZ2457|MH838146
Neoleptophlebia heteronea|EVOTR883-12|0001141|United States|BOLD:AAZ2457|MH841519
Neoleptophlebia heteronea|EVOTR1979-13|0004713|United States|BOLD:AAZ2457|MH841801
Neoleptophlebia heteronea|EVOTR1983-13|0004717|United States|BOLD:AAZ2457|MH840898
Neoleptophlebia heteronea|EVOTR1988-13|0004722|United States|BOLD:AAZ2457|MH838311
Neoleptophlebia heteronea|EVOTR2288-13|0001128|United States|BOLD:AAZ2457|MH839870
Neoleptophlebia heteronea|EVOTR899-12|0000864|United States|BOLD:AAZ2457|MH839221
Neoleptophlebia heteronea|EVOTR879-12|0001136|United States|BOLD:AAZ2457|MH838870
Neoleptophlebia heteronea|CREPH052-16|E52-CR1|Canada|BOLD:AAZ2457|MH917021
Neoleptophlebia heteronea|CREPH162-16|E-162-CR1S|Canada|BOLD:AAZ2457|MH917039
Neoleptophlebia heteronea|CREPH062-16|E62-CR1|Canada|BOLD:AAZ2457|MH917015
Neoleptophlebia heteronea|CREPH047-16|E47-CR1|Canada|BOLD:AAZ2457|MH917034
Neoleptophlebia heteronea|CREPH097-16|E97-CR1M|Canada|BOLD:AAZ2457|MH917025
Neoleptophlebia heteronea|CREPH098-16|E-98-CR1M|Canada|BOLD:AAZ2457|MH916917
Neoleptophlebia heteronea|CREPH149-16|E-149-CR2BS|Canada|BOLD:AAZ2457|MH916977
Neoleptophlebia heteronea|CREPH153-16|E-153-CR1S|Canada|BOLD:AAZ2457|MH916932
Neoleptophlebia heteronea|CREPH008-16|E8-CR4|Canada|BOLD:AAZ2457|MH916978
Neoleptophlebia heteronea|CREPH107-16|E-107-CR1UM|Canada|BOLD:AAZ2457|MH916892
Neoleptophlebia memorialis|LJMAY260-11|BIOUG00640-B02|United States|BOLD:ABV8249
Neoleptophlebia memorialis|CREPH091-16|E91-CR2|Canada|BOLD:AAZ4066|MH916956
Neoleptophlebia memorialis|LJMAY263-11|BIOUG00640-B05|United States|BOLD:AAZ4066|JQ662635
Neoleptophlebia memorialis|LJMAY262-11|BIOUG00640-B04|United States|BOLD:AAZ4066|JQ661982
Neoleptophlebia memorialis|EVOTR1978-13|0004712|United States|BOLD:AAZ4066|MH838151
Paraleptophlebia sp.JMW1|LJMAY234-11|BIOUG00639-G11|United States|BOLD:AAZ2514|JQ662074
Paraleptophlebia sp.JMW1|LJMAY233-11|BIOUG00639-G10|United States|BOLD:AAZ2514|JQ662106
Paraleptophlebia|GMNCJ054-13|BIOUG06945-F11|United States|BOLD:ACI2986
Paraleptophlebia werneri|FIEPT290-16|JI-BOLD-567|Finland|BOLD:ADD0736|MZ624375
Paraleptophlebia werneri|FIEPT289-16|JI-BOLD-566|Finland|BOLD:ADD0736|MZ627894
Paraleptophlebia werneri|FIEPT293-16|JI-BOLD-570|Finland|BOLD:ADD0736|MZ625584
Paraleptophlebia werneri|FIEPT292-16|JI-BOLD-569|Finland|BOLD:ADD0736|MZ627061
Paraleptophlebia werneri|FIEPT291-16|JI-BOLD-568|Finland|BOLD:ADD0736|MZ628579
Paraleptophlebia werneri|FIEPT288-16|JI-BOLD-565|Finland|BOLD:ADD0736|MZ623399
Paraleptophlebia werneri|FIEPT287-16|JI-BOLD-564|Finland|BOLD:ADD0736|MZ623174
Paraleptophlebia submarginata|GST024-15|Psu_BBT130519_01|Germany|BOLD:AAK5109|KY261941
Paraleptophlebia submarginata|BGMAY324-11|CCDB-09355-H03|Bulgaria|BOLD:AAK5109
Paraleptophlebia submarginata|GST022-15|Psu_BBT130517_02|Germany|BOLD:AAK5109|KY262118
Paraleptophlebia submarginata|GST028-15|Psu_BBT130602_01|Germany|BOLD:AAK5109|KY262486
Paraleptophlebia submarginata|GST032-15|Psu_BBT130616_01|Germany|BOLD:AAK5109|KY261310
Paraleptophlebia submarginata|GST033-15|Psu_BBT130616_02|Germany|BOLD:AAK5109|KY261575
Paraleptophlebia submarginata|GST037-15|Psu_BBT130624_01|Germany|BOLD:AAK5109|KY261572
Paraleptophlebia submarginata|GBEPT137-13|GBOL00613|Germany|BOLD:AAK5109|KY261923
Paraleptophlebia submarginata|GST030-15|Psu_BBT130610_02|Germany|BOLD:AAK5109|KY262504
Paraleptophlebia submarginata|GST031-15|Psu_BBT130612_01|Germany|BOLD:AAK5109|KY262131
Paraleptophlebia submarginata|GST025-15|Psu_BBT130519_02|Germany|BOLD:AAK5109|KY261742
Paraleptophlebia submarginata|GST021-15|Psu_BBT130517_01|Germany|BOLD:AAK5109|KY261397
Paraleptophlebia submarginata|GST020-15|Psu_BBT130508_01|Germany|BOLD:AAK5109|KY261512
Paraleptophlebia submarginata|TRSKA4556-20|BIOUG57628-H07|Montenegro|BOLD:AAK5109

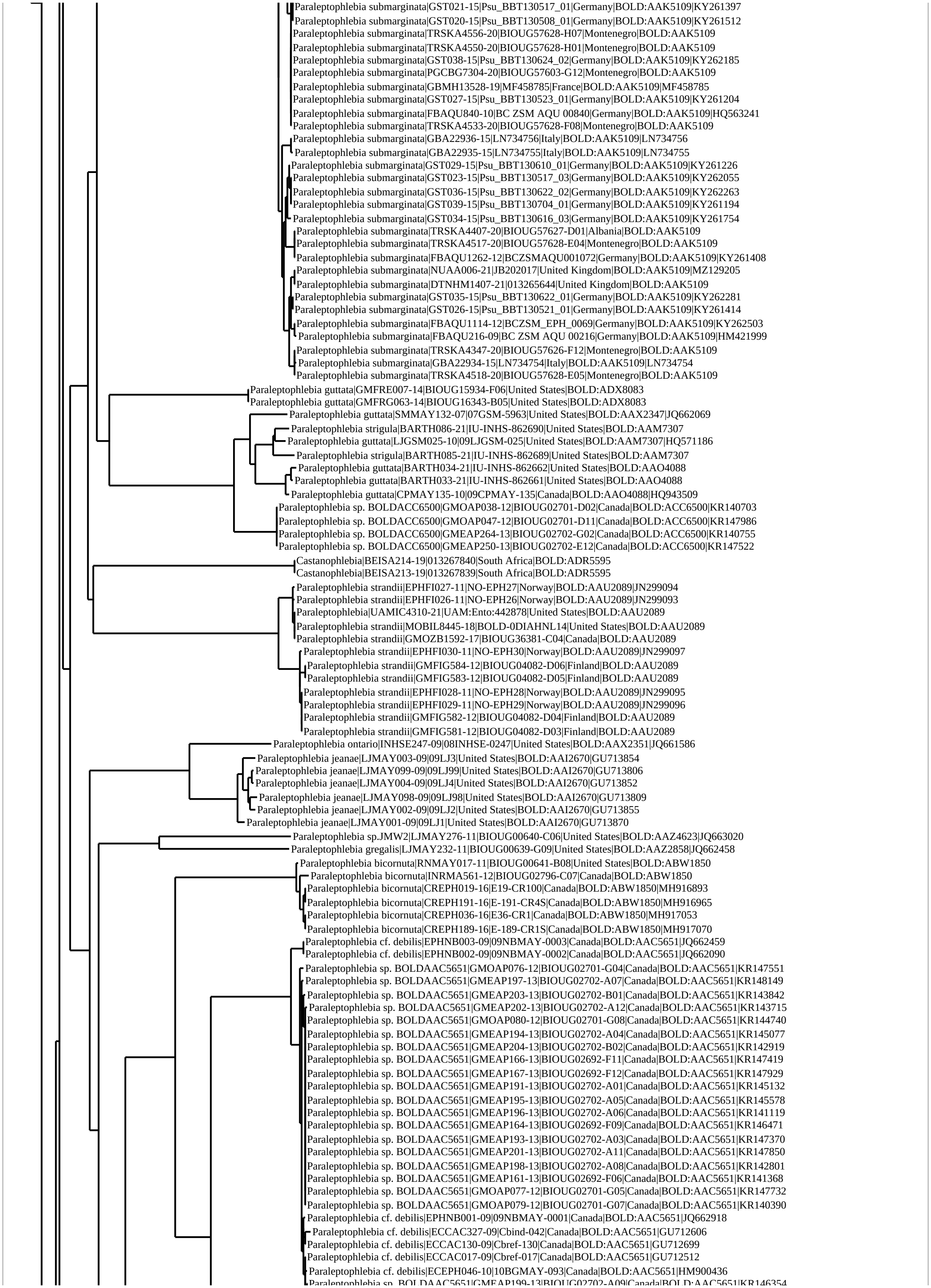
Paraleptophlebia submarginata|GST021-15|Psu_BBT130517_01|Germany|BOLD:AAK5109|KY261397
Paraleptophlebia submarginata|GST020-15|Psu_BBT130508_01|Germany|BOLD:AAK5109|KY261512
Paraleptophlebia submarginata|TRSKA4556-20|BIOUG57628-H07|Montenegro|BOLD:AAK5109
Paraleptophlebia submarginata|TRSKA4550-20|BIOUG57628-H01|Montenegro|BOLD:AAK5109
Paraleptophlebia submarginata|GST038-15|Psu_BBT130624_02|Germany|BOLD:AAK5109|KY262185
Paraleptophlebia submarginata|PGCBG7304-20|BIOUG57603-G12|Montenegro|BOLD:AAK5109
Paraleptophlebia submarginata|GBMH13528-19|MF458785|France|BOLD:AAK5109|MF458785
Paraleptophlebia submarginata|GST027-15|Psu_BBT130523_01|Germany|BOLD:AAK5109|KY261204
Paraleptophlebia submarginata|FBAQU840-10|BC ZSM AQU 00840|Germany|BOLD:AAK5109|HQ563241
Paraleptophlebia submarginata|TRSKA4533-20|BIOUG57628-F08|Montenegro|BOLD:AAK5109
Paraleptophlebia submarginata|GBA22936-15|LN734756|Italy|BOLD:AAK5109|LN734756
Paraleptophlebia submarginata|GBA22935-15|LN734755|Italy|BOLD:AAK5109|LN734755
Paraleptophlebia submarginata|GST029-15|Psu_BBT130610_01|Germany|BOLD:AAK5109|KY261226
Paraleptophlebia submarginata|GST023-15|Psu_BBT130517_03|Germany|BOLD:AAK5109|KY262055
Paraleptophlebia submarginata|GST036-15|Psu_BBT130622_02|Germany|BOLD:AAK5109|KY262263
Paraleptophlebia submarginata|GST039-15|Psu_BBT130704_01|Germany|BOLD:AAK5109|KY261194
Paraleptophlebia submarginata|GST034-15|Psu_BBT130616_03|Germany|BOLD:AAK5109|KY261754
Paraleptophlebia submarginata|TRSKA4407-20|BIOUG57627-D01|Albania|BOLD:AAK5109
Paraleptophlebia submarginata|TRSKA4517-20|BIOUG57628-E04|Montenegro|BOLD:AAK5109
Paraleptophlebia submarginata|FBAQU1262-12|BCZSMAQU001072|Germany|BOLD:AAK5109|KY261408
Paraleptophlebia submarginata|NUAA006-21|JB202017|United Kingdom|BOLD:AAK5109|MZ129205
Paraleptophlebia submarginata|DTNHM1407-21|013265644|United Kingdom|BOLD:AAK5109
Paraleptophlebia submarginata|GST035-15|Psu_BBT130622_01|Germany|BOLD:AAK5109|KY262281
Paraleptophlebia submarginata|GST026-15|Psu_BBT130521_01|Germany|BOLD:AAK5109|KY261414
Paraleptophlebia submarginata|FBAQU1114-12|BCZSM_EPH_0069|Germany|BOLD:AAK5109|KY262503
Paraleptophlebia submarginata|FBAQU216-09|BC ZSM AQU 00216|Germany|BOLD:AAK5109|HM421999
Paraleptophlebia submarginata|TRSKA4347-20|BIOUG57626-F12|Montenegro|BOLD:AAK5109
Paraleptophlebia submarginata|GBA22934-15|LN734754|Italy|BOLD:AAK5109|LN734754
Paraleptophlebia submarginata|TRSKA4518-20|BIOUG57628-E05|Montenegro|BOLD:AAK5109
Paraleptophlebia guttata|GMFRE007-14|BIOUG15934-F06|United States|BOLD:ADX8083
Paraleptophlebia guttata|GMFRG063-14|BIOUG16343-B05|United States|BOLD:ADX8083
Paraleptophlebia guttata|SMMAY132-07|07GSM-5963|United States|BOLD:AAX2347|JQ662069
Paraleptophlebia strigula|BARTH086-21|IU-INHS-862690|United States|BOLD:AAM7307
Paraleptophlebia guttata|LJGSM025-10|09LJGSM-025|United States|BOLD:AAM7307|HQ571186
Paraleptophlebia strigula|BARTH085-21|IU-INHS-862689|United States|BOLD:AAM7307
Paraleptophlebia guttata|BARTH034-21|IU-INHS-862662|United States|BOLD:AAO4088
Paraleptophlebia guttata|BARTH033-21|IU-INHS-862661|United States|BOLD:AAO4088
Paraleptophlebia guttata|CPMAY135-10|09CPMAY-135|Canada|BOLD:AAO4088|HQ943509
Paraleptophlebia sp. BOLDACC6500|GMOAP038-12|BIOUG02701-D02|Canada|BOLD:ACC6500|KR140703
Paraleptophlebia sp. BOLDACC6500|GMOAP047-12|BIOUG02701-D11|Canada|BOLD:ACC6500|KR147986
Paraleptophlebia sp. BOLDACC6500|GMEAP264-13|BIOUG02702-G02|Canada|BOLD:ACC6500|KR140755
Paraleptophlebia sp. BOLDACC6500|GMEAP250-13|BIOUG02702-E12|Canada|BOLD:ACC6500|KR147522
Castanophlebia|BEISA214-19|013267840|South Africa|BOLD:ADR5595
Castanophlebia|BEISA213-19|013267839|South Africa|BOLD:ADR5595
Paraleptophlebia strandii|EPHFI027-11|NO-EPH27|Norway|BOLD:AAU2089|JN299094
Paraleptophlebia strandii|EPHFI026-11|NO-EPH26|Norway|BOLD:AAU2089|JN299093
Paraleptophlebia|UAMIC4310-21|UAM:Ento:442878|United States|BOLD:AAU2089
Paraleptophlebia strandii|MOBIL8445-18|BOLD-0DIAHNL14|United States|BOLD:AAU2089
Paraleptophlebia strandii|GMOZB1592-17|BIOUG36381-C04|Canada|BOLD:AAU2089
Paraleptophlebia strandii|EPHFI030-11|NO-EPH30|Norway|BOLD:AAU2089|JN299097
Paraleptophlebia strandii|GMFIG584-12|BIOUG04082-D06|Finland|BOLD:AAU2089
Paraleptophlebia strandii|GMFIG583-12|BIOUG04082-D05|Finland|BOLD:AAU2089
Paraleptophlebia strandii|EPHFI028-11|NO-EPH28|Norway|BOLD:AAU2089|JN299095
Paraleptophlebia strandii|EPHFI029-11|NO-EPH29|Norway|BOLD:AAU2089|JN299096
Paraleptophlebia strandii|GMFIG582-12|BIOUG04082-D04|Finland|BOLD:AAU2089
Paraleptophlebia strandii|GMFIG581-12|BIOUG04082-D03|Finland|BOLD:AAU2089
Paraleptophlebia ontario|INHSE247-09|08INHSE-0247|United States|BOLD:AAX2351|JQ661586
Paraleptophlebia jeanae|LJMAY003-09|09LJ3|United States|BOLD:AAI2670|GU713854
Paraleptophlebia jeanae|LJMAY099-09|09LJ99|United States|BOLD:AAI2670|GU713806
Paraleptophlebia jeanae|LJMAY004-09|09LJ4|United States|BOLD:AAI2670|GU713852
Paraleptophlebia jeanae|LJMAY098-09|09LJ98|United States|BOLD:AAI2670|GU713809
Paraleptophlebia jeanae|LJMAY002-09|09LJ2|United States|BOLD:AAI2670|GU713855
Paraleptophlebia jeanae|LJMAY001-09|09LJ1|United States|BOLD:AAI2670|GU713870
Paraleptophlebia sp.JMW2|LJMAY276-11|BIOUG00640-C06|United States|BOLD:AAZ4623|JQ663020
Paraleptophlebia gregalis|LJMAY232-11|BIOUG00639-G09|United States|BOLD:AAZ2858|JQ662458
Paraleptophlebia bicornuta|RNMAY017-11|BIOUG00641-B08|United States|BOLD:ABW1850
Paraleptophlebia bicornuta|INRMA561-12|BIOUG02796-C07|Canada|BOLD:ABW1850
Paraleptophlebia bicornuta|CREPH019-16|E19-CR100|Canada|BOLD:ABW1850|MH916893
Paraleptophlebia bicornuta|CREPH191-16|E-191-CR4S|Canada|BOLD:ABW1850|MH916965
Paraleptophlebia bicornuta|CREPH036-16|E36-CR1|Canada|BOLD:ABW1850|MH917053
Paraleptophlebia bicornuta|CREPH189-16|E-189-CR1S|Canada|BOLD:ABW1850|MH917070
Paraleptophlebia cf. debilis|EPHNB003-09|09NBMAY-0003|Canada|BOLD:AAC5651|JQ662459
Paraleptophlebia cf. debilis|EPHNB002-09|09NBMAY-0002|Canada|BOLD:AAC5651|JQ662090
Paraleptophlebia sp. BOLDAAC5651|GMOAP076-12|BIOUG02701-G04|Canada|BOLD:AAC5651|KR147551
Paraleptophlebia sp. BOLDAAC5651|GMEAP197-13|BIOUG02702-A07|Canada|BOLD:AAC5651|KR148149
Paraleptophlebia sp. BOLDAAC5651|GMEAP203-13|BIOUG02702-B01|Canada|BOLD:AAC5651|KR143842
Paraleptophlebia sp. BOLDAAC5651|GMEAP202-13|BIOUG02702-A12|Canada|BOLD:AAC5651|KR143715
Paraleptophlebia sp. BOLDAAC5651|GMOAP080-12|BIOUG02701-G08|Canada|BOLD:AAC5651|KR144740
Paraleptophlebia sp. BOLDAAC5651|GMEAP194-13|BIOUG02702-A04|Canada|BOLD:AAC5651|KR145077
Paraleptophlebia sp. BOLDAAC5651|GMEAP204-13|BIOUG02702-B02|Canada|BOLD:AAC5651|KR142919
Paraleptophlebia sp. BOLDAAC5651|GMEAP166-13|BIOUG02692-F11|Canada|BOLD:AAC5651|KR147419
Paraleptophlebia sp. BOLDAAC5651|GMEAP167-13|BIOUG02692-F12|Canada|BOLD:AAC5651|KR147929
Paraleptophlebia sp. BOLDAAC5651|GMEAP191-13|BIOUG02702-A01|Canada|BOLD:AAC5651|KR145132
Paraleptophlebia sp. BOLDAAC5651|GMEAP195-13|BIOUG02702-A05|Canada|BOLD:AAC5651|KR145578
Paraleptophlebia sp. BOLDAAC5651|GMEAP196-13|BIOUG02702-A06|Canada|BOLD:AAC5651|KR141119
Paraleptophlebia sp. BOLDAAC5651|GMEAP164-13|BIOUG02692-F09|Canada|BOLD:AAC5651|KR146471
Paraleptophlebia sp. BOLDAAC5651|GMEAP193-13|BIOUG02702-A03|Canada|BOLD:AAC5651|KR147370
Paraleptophlebia sp. BOLDAAC5651|GMEAP201-13|BIOUG02702-A11|Canada|BOLD:AAC5651|KR147850
Paraleptophlebia sp. BOLDAAC5651|GMEAP198-13|BIOUG02702-A08|Canada|BOLD:AAC5651|KR142801
Paraleptophlebia sp. BOLDAAC5651|GMEAP161-13|BIOUG02692-F06|Canada|BOLD:AAC5651|KR141368
Paraleptophlebia sp. BOLDAAC5651|GMOAP077-12|BIOUG02701-G05|Canada|BOLD:AAC5651|KR147732
Paraleptophlebia sp. BOLDAAC5651|GMOAP079-12|BIOUG02701-G07|Canada|BOLD:AAC5651|KR140390
Paraleptophlebia cf. debilis|EPHNB001-09|09NBMAY-0001|Canada|BOLD:AAC5651|JQ662918
Paraleptophlebia cf. debilis|ECCAC327-09|Cbind-042|Canada|BOLD:AAC5651|GU712606
Paraleptophlebia cf. debilis|ECCAC130-09|Cbref-130|Canada|BOLD:AAC5651|GU712699
Paraleptophlebia cf. debilis|ECCAC017-09|Cbref-017|Canada|BOLD:AAC5651|GU712512
Paraleptophlebia cf. debilis|ECEPH046-10|10BGMAY-093|Canada|BOLD:AAC5651|HM900436
Paraleptophlebia sp. BOLDAAC5651|GMEAP199-13|BIOUG02702-A09|Canada|BOLD:AAC5651|KR146354

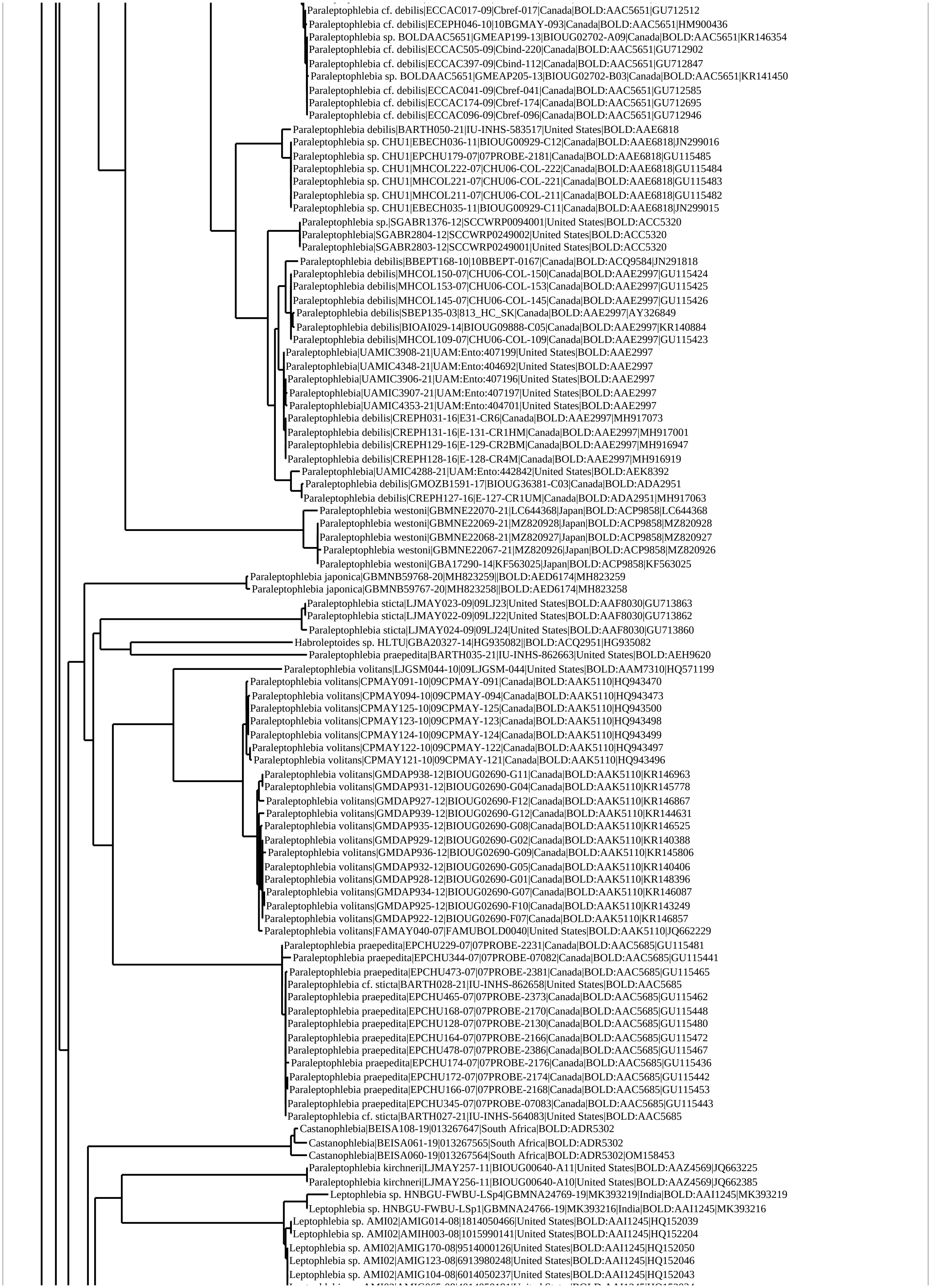
Paraleptophlebia cf. debilis|ECCAC017-09|Cbref-017|Canada|BOLD:AAC5651|GU712512
Paraleptophlebia cf. debilis|ECEPH046-10|10BGMAY-093|Canada|BOLD:AAC5651|HM900436
Paraleptophlebia sp. BOLDAAC5651|GMEAP199-13|BIOUG02702-A09|Canada|BOLD:AAC5651|KR146354
Paraleptophlebia cf. debilis|ECCAC505-09|Cbind-220|Canada|BOLD:AAC5651|GU712902
Paraleptophlebia cf. debilis|ECCAC397-09|Cbind-112|Canada|BOLD:AAC5651|GU712847
Paraleptophlebia sp. BOLDAAC5651|GMEAP205-13|BIOUG02702-B03|Canada|BOLD:AAC5651|KR141450
Paraleptophlebia cf. debilis|ECCAC041-09|Cbref-041|Canada|BOLD:AAC5651|GU712585
Paraleptophlebia cf. debilis|ECCAC174-09|Cbref-174|Canada|BOLD:AAC5651|GU712695
Paraleptophlebia cf. debilis|ECCAC096-09|Cbref-096|Canada|BOLD:AAC5651|GU712946
Paraleptophlebia debilis|BARTH050-21|IU-INHS-583517|United States|BOLD:AAE6818
Paraleptophlebia sp. CHU1|EBECH036-11|BIOUG00929-C12|Canada|BOLD:AAE6818|JN299016
Paraleptophlebia sp. CHU1|EPCHU179-07|07PROBE-2181|Canada|BOLD:AAE6818|GU115485
Paraleptophlebia sp. CHU1|MHCOL222-07|CHU06-COL-222|Canada|BOLD:AAE6818|GU115484
Paraleptophlebia sp. CHU1|MHCOL221-07|CHU06-COL-221|Canada|BOLD:AAE6818|GU115483
Paraleptophlebia sp. CHU1|MHCOL211-07|CHU06-COL-211|Canada|BOLD:AAE6818|GU115482
Paraleptophlebia sp. CHU1|EBECH035-11|BIOUG00929-C11|Canada|BOLD:AAE6818|JN299015
Paraleptophlebia sp.|SGABR1376-12|SCCWRP0094001|United States|BOLD:ACC5320
Paraleptophlebia|SGABR2804-12|SCCWRP0249002|United States|BOLD:ACC5320
Paraleptophlebia|SGABR2803-12|SCCWRP0249001|United States|BOLD:ACC5320
Paraleptophlebia debilis|BBEPT168-10|10BBEPT-0167|Canada|BOLD:ACQ9584|JN291818
Paraleptophlebia debilis|MHCOL150-07|CHU06-COL-150|Canada|BOLD:AAE2997|GU115424
Paraleptophlebia debilis|MHCOL153-07|CHU06-COL-153|Canada|BOLD:AAE2997|GU115425
Paraleptophlebia debilis|MHCOL145-07|CHU06-COL-145|Canada|BOLD:AAE2997|GU115426
Paraleptophlebia debilis|SBEP135-03|813_HC_SK|Canada|BOLD:AAE2997|AY326849
Paraleptophlebia debilis|BIOAI029-14|BIOUG09888-C05|Canada|BOLD:AAE2997|KR140884
Paraleptophlebia debilis|MHCOL109-07|CHU06-COL-109|Canada|BOLD:AAE2997|GU115423
Paraleptophlebia|UAMIC3908-21|UAM:Ento:407199|United States|BOLD:AAE2997
Paraleptophlebia|UAMIC4348-21|UAM:Ento:404692|United States|BOLD:AAE2997
Paraleptophlebia|UAMIC3906-21|UAM:Ento:407196|United States|BOLD:AAE2997
Paraleptophlebia|UAMIC3907-21|UAM:Ento:407197|United States|BOLD:AAE2997
Paraleptophlebia|UAMIC4353-21|UAM:Ento:404701|United States|BOLD:AAE2997
Paraleptophlebia debilis|CREPH031-16|E31-CR6|Canada|BOLD:AAE2997|MH917073
Paraleptophlebia debilis|CREPH131-16|E-131-CR1HM|Canada|BOLD:AAE2997|MH917001
Paraleptophlebia debilis|CREPH129-16|E-129-CR2BM|Canada|BOLD:AAE2997|MH916947
Paraleptophlebia debilis|CREPH128-16|E-128-CR4M|Canada|BOLD:AAE2997|MH916919
Paraleptophlebia|UAMIC4288-21|UAM:Ento:442842|United States|BOLD:AEK8392
Paraleptophlebia debilis|GMOZB1591-17|BIOUG36381-C03|Canada|BOLD:ADA2951
Paraleptophlebia debilis|CREPH127-16|E-127-CR1UM|Canada|BOLD:ADA2951|MH917063
Paraleptophlebia westoni|GBMNE22070-21|LC644368|Japan|BOLD:ACP9858|LC644368
Paraleptophlebia westoni|GBMNE22069-21|MZ820928|Japan|BOLD:ACP9858|MZ820928
Paraleptophlebia westoni|GBMNE22068-21|MZ820927|Japan|BOLD:ACP9858|MZ820927
Paraleptophlebia westoni|GBMNE22067-21|MZ820926|Japan|BOLD:ACP9858|MZ820926
Paraleptophlebia westoni|GBA17290-14|KF563025|Japan|BOLD:ACP9858|KF563025
Paraleptophlebia japonica|GBMNB59768-20|MH823259||BOLD:AED6174|MH823259
Paraleptophlebia japonica|GBMNB59767-20|MH823258||BOLD:AED6174|MH823258
Paraleptophlebia sticta|LJMAY023-09|09LJ23|United States|BOLD:AAF8030|GU713863
Paraleptophlebia sticta|LJMAY022-09|09LJ22|United States|BOLD:AAF8030|GU713862
Paraleptophlebia sticta|LJMAY024-09|09LJ24|United States|BOLD:AAF8030|GU713860
Habroleptoides sp. HLTU|GBA20327-14|HG935082||BOLD:ACQ2951|HG935082
Paraleptophlebia praepedita|BARTH035-21|IU-INHS-862663|United States|BOLD:AEH9620
Paraleptophlebia volitans|LJGSM044-10|09LJGSM-044|United States|BOLD:AAM7310|HQ571199
Paraleptophlebia volitans|CPMAY091-10|09CPMAY-091|Canada|BOLD:AAK5110|HQ943470
Paraleptophlebia volitans|CPMAY094-10|09CPMAY-094|Canada|BOLD:AAK5110|HQ943473
Paraleptophlebia volitans|CPMAY125-10|09CPMAY-125|Canada|BOLD:AAK5110|HQ943500
Paraleptophlebia volitans|CPMAY123-10|09CPMAY-123|Canada|BOLD:AAK5110|HQ943498
Paraleptophlebia volitans|CPMAY124-10|09CPMAY-124|Canada|BOLD:AAK5110|HQ943499
Paraleptophlebia volitans|CPMAY122-10|09CPMAY-122|Canada|BOLD:AAK5110|HQ943497
Paraleptophlebia volitans|CPMAY121-10|09CPMAY-121|Canada|BOLD:AAK5110|HQ943496
Paraleptophlebia volitans|GMDAP938-12|BIOUG02690-G11|Canada|BOLD:AAK5110|KR146963
Paraleptophlebia volitans|GMDAP931-12|BIOUG02690-G04|Canada|BOLD:AAK5110|KR145778
Paraleptophlebia volitans|GMDAP927-12|BIOUG02690-F12|Canada|BOLD:AAK5110|KR146867
Paraleptophlebia volitans|GMDAP939-12|BIOUG02690-G12|Canada|BOLD:AAK5110|KR144631
Paraleptophlebia volitans|GMDAP935-12|BIOUG02690-G08|Canada|BOLD:AAK5110|KR146525
Paraleptophlebia volitans|GMDAP929-12|BIOUG02690-G02|Canada|BOLD:AAK5110|KR140388
Paraleptophlebia volitans|GMDAP936-12|BIOUG02690-G09|Canada|BOLD:AAK5110|KR145806
Paraleptophlebia volitans|GMDAP932-12|BIOUG02690-G05|Canada|BOLD:AAK5110|KR140406
Paraleptophlebia volitans|GMDAP928-12|BIOUG02690-G01|Canada|BOLD:AAK5110|KR148396
Paraleptophlebia volitans|GMDAP934-12|BIOUG02690-G07|Canada|BOLD:AAK5110|KR146087
Paraleptophlebia volitans|GMDAP925-12|BIOUG02690-F10|Canada|BOLD:AAK5110|KR143249
Paraleptophlebia volitans|GMDAP922-12|BIOUG02690-F07|Canada|BOLD:AAK5110|KR146857
Paraleptophlebia volitans|FAMAY040-07|FAMUBOLD0040|United States|BOLD:AAK5110|JQ662229
Paraleptophlebia praepedita|EPCHU229-07|07PROBE-2231|Canada|BOLD:AAC5685|GU115481
Paraleptophlebia praepedita|EPCHU344-07|07PROBE-07082|Canada|BOLD:AAC5685|GU115441
Paraleptophlebia praepedita|EPCHU473-07|07PROBE-2381|Canada|BOLD:AAC5685|GU115465
Paraleptophlebia cf. sticta|BARTH028-21|IU-INHS-862658|United States|BOLD:AAC5685
Paraleptophlebia praepedita|EPCHU465-07|07PROBE-2373|Canada|BOLD:AAC5685|GU115462
Paraleptophlebia praepedita|EPCHU168-07|07PROBE-2170|Canada|BOLD:AAC5685|GU115448
Paraleptophlebia praepedita|EPCHU128-07|07PROBE-2130|Canada|BOLD:AAC5685|GU115480
Paraleptophlebia praepedita|EPCHU164-07|07PROBE-2166|Canada|BOLD:AAC5685|GU115472
Paraleptophlebia praepedita|EPCHU478-07|07PROBE-2386|Canada|BOLD:AAC5685|GU115467
Paraleptophlebia praepedita|EPCHU174-07|07PROBE-2176|Canada|BOLD:AAC5685|GU115436
Paraleptophlebia praepedita|EPCHU172-07|07PROBE-2174|Canada|BOLD:AAC5685|GU115442
Paraleptophlebia praepedita|EPCHU166-07|07PROBE-2168|Canada|BOLD:AAC5685|GU115453
Paraleptophlebia praepedita|EPCHU345-07|07PROBE-07083|Canada|BOLD:AAC5685|GU115443
Paraleptophlebia cf. sticta|BARTH027-21|IU-INHS-564083|United States|BOLD:AAC5685
Castanophlebia|BEISA108-19|013267647|South Africa|BOLD:ADR5302
Castanophlebia|BEISA061-19|013267565|South Africa|BOLD:ADR5302
Castanophlebia|BEISA060-19|013267564|South Africa|BOLD:ADR5302|OM158453
Paraleptophlebia kirchneri|LJMAY257-11|BIOUG00640-A11|United States|BOLD:AAZ4569|JQ663225
Paraleptophlebia kirchneri|LJMAY256-11|BIOUG00640-A10|United States|BOLD:AAZ4569|JQ662385
Leptophlebia sp. HNBGU-FWBU-LSp4|GBMNA24769-19|MK393219|India|BOLD:AAI1245|MK393219
Leptophlebia sp. HNBGU-FWBU-LSp1|GBMNA24766-19|MK393216|India|BOLD:AAI1245|MK393216
Leptophlebia sp. AMI02|AMIG014-08|1814050466|United States|BOLD:AAI1245|HQ152039
Leptophlebia sp. AMI02|AMIH003-08|1015990141|United States|BOLD:AAI1245|HQ152204
Leptophlebia sp. AMI02|AMIG170-08|9514000126|United States|BOLD:AAI1245|HQ152050
Leptophlebia sp. AMI02|AMIG123-08|6913980248|United States|BOLD:AAI1245|HQ152046
Leptophlebia sp. AMI02|AMIG104-08|6014050237|United States|BOLD:AAI1245|HQ152043

[illegible]
Leptophlebia sp. AMI02|AMIG123-08|6913980248|United States|BOLD:AAI1245|HQ152046
Leptophlebia sp. AMI02|AMIG104-08|6014050237|United States|BOLD:AAI1245|HQ152043
Leptophlebia sp. AMI02|AMIG065-08|4014050191|United States|BOLD:AAI1245|HQ152034
Leptophlebia intermedia|INHSE240-09|08INHSE-0240|United States|BOLD:AAC8966|HQ568791
Leptophlebia intermedia|AMI066-07|420033554|United States|BOLD:AAA7019|HQ150292
Leptophlebia intermedia|AMIE039-07|600033753|United States|BOLD:AAA7019|HQ151461
Leptophlebia intermedia|AMI015-07|160034272|United States|BOLD:AAA7019|HQ150290
Leptophlebia intermedia|AMI132-07|790034495|United States|BOLD:AAA7019|HQ150294
Leptophlebia intermedia|GMOAP074-12|BIOUG02701-G02|Canada|BOLD:AAA7019|KR145029
Leptophlebia intermedia|AMI038-07|280033938|United States|BOLD:AAA7019|HQ150291
Leptophlebia intermedia|AMI109-07|680034055|United States|BOLD:AAA7019|HQ150293
Leptophlebia intermedia|SBEP149-03|99_SL_ON|Canada|BOLD:AAA7019|AY326898
Leptophlebia intermedia|GMEAP120-13|BIOUG02692-C01|Canada|BOLD:AAA7019|KR145209
Leptophlebia intermedia|AMIG069-08|4114130170|United States|BOLD:AAA7019|HQ152035
Leptophlebia intermedia|AMIG218-08|4314210246|United States|BOLD:AAA7019|HQ152054
Leptophlebia intermedia|AMIG088-08|5014210106|United States|BOLD:AAA7019|HQ152020
Leptophlebia intermedia|AMI006-07|120034335|United States|BOLD:AAA7019|HQ150289
Leptophlebia intermedia|AMI004-07|110033822|United States|BOLD:AAA7019|HQ150288
Leptophlebia intermedia|SBEP148-03|98_SL_ON|United States|BOLD:AAA7019|AY326892
Leptophlebia intermedia|AMIG285-09|4414060124|United States|BOLD:AAA7019|HQ152030
Leptophlebia intermedia|AMIG059-08|3814150595|United States|BOLD:AAA7019|HQ152033
Leptophlebia intermedia|SBGB058-03|AY165686||BOLD:AAA7019|AY165686
Leptophlebia intermedia|CNLMK057-14|BIOUG13733-A01|Canada|BOLD:AAA7019|KR379231
Leptophlebia intermedia|SBEP008-03|109B_PP_ME|United States|BOLD:AAA7019|AY326893
Leptophlebia intermedia|SBEP017-03|15_MP_ME|United States|BOLD:AAA7019|AY326856
Leptophlebia intermedia|SBEP010-03|110A_PP_ME|United States|BOLD:AAA7019|AY326943
Leptophlebia intermedia|AMIG190-08|6114010159|United States|BOLD:AAA7019|HQ152022
Leptophlebia intermedia|AMIG178-08|2114040136|United States|BOLD:AAA7019|HQ152052
Leptophlebia intermedia|AMIG212-08|3014030235|United States|BOLD:AAA7019|HQ152026
Leptophlebia intermedia|AMIG201-08|5914020168|United States|BOLD:AAA7019|HQ152025
Leptophlebia sp. BOLDAAX5673|GMEAP227-13|BIOUG02702-D01|Canada|BOLD:ACL7357|KR146184
Leptophlebia sp.XZ1|ONMAY141-08|08ONMAY-0141|Canada|BOLD:ACL7357|JQ662984
Leptophlebia sp. BOLDAAX5673|GMEAP080-13|BIOUG02691-G08|Canada|BOLD:ACL7357|KR147439
Leptophlebia|SWBRP391-17|BIOUG36686-E10|Canada|BOLD:ACL7357
Leptophlebia sp. XZ1|AMIG277-08|8014090153|United States|BOLD:AAX5673|HQ152057
Leptophlebia sp. BOLDAAX5673|GMEAP242-13|BIOUG02702-E04|Canada|BOLD:ACL7120|KR147212
Leptophlebia sp. BOLDAAX5673|GMEAP226-13|BIOUG02702-C12|Canada|BOLD:ACL7120|KR144084
Leptophlebia sp. BOLDAAX5673|GMEAP229-13|BIOUG02702-D03|Canada|BOLD:ACL7120|KR146698
Leptophlebia sp. BOLDAAX5673|GMEAP228-13|BIOUG02702-D02|Canada|BOLD:ACL7120|KR145148
Leptophlebia sp. BOLDAAX5673|GMEAP239-13|BIOUG02702-E01|Canada|BOLD:ACL7120|KR141389
Leptophlebia sp. ROM 2010628 EGB005|GBMH12108-19|KJ674893|Canada|BOLD:ACL7120|KJ674893
Leptophlebia sp. BOLDAAX5673|GMEAP245-13|BIOUG02702-E07|Canada|BOLD:ACL7120|KR142894
Leptophlebia sp. BOLDAAX5673|GMEAP065-13|BIOUG02691-F05|Canada|BOLD:ACL7120|KR143229
Leptophlebia sp. BOLDAAX5673|GMEAP121-13|BIOUG02692-C02|Canada|BOLD:ACL7120|KR144461
Leptophlebia sp. BOLDAAX5673|GMEAP238-13|BIOUG02702-D12|Canada|BOLD:ACL7120|KR143507
Leptophlebia|POROM065-19|CCDB-33288-F05|Canada|BOLD:ACL7120
Leptophlebia|POROM064-19|CCDB-33288-F04|Canada|BOLD:ACL7120
Leptophlebia sp. BOLDAAX5673|GMEAP042-13|BIOUG02691-D06|Canada|BOLD:ACL7120|KR142148
Leptophlebia sp. BOLDAAX5673|GMEAP240-13|BIOUG02702-E02|Canada|BOLD:ACL7120|KR141874
Leptophlebia sp. BOLDAAX5673|GMEAP235-13|BIOUG02702-D09|Canada|BOLD:ACL6896|KR143347
Leptophlebia sp. BOLDAAX5673|GMEAP237-13|BIOUG02702-D11|Canada|BOLD:ACL6896|KR142978
Leptophlebia|CNBRK077-14|BIOUG10540-C06|Canada|BOLD:ACL6896|KR381134
Leptophlebia cupida|AMIH166-08|7215980846|United States|BOLD:AAA7018|HQ152203
Leptophlebia cupida|AMIG016-08|1914040712|United States|BOLD:AAA7018|HQ152040
Leptophlebia cupida|AMIH104-08|5115980950|United States|BOLD:AAA7018|HQ152200
Leptophlebia cupida|AMIG055-08|3613990332|United States|BOLD:AAA7018|HQ152028
Leptophlebia cupida|AMIH021-08|1715980709|United States|BOLD:AAA7018|HQ152195
Leptophlebia cupida|AMIG001-08|1013990278|United States|BOLD:AAA7018|HQ152029
Leptophlebia cupida|AMIG031-08|2413990140|United States|BOLD:AAA7018|HQ152027
Leptophlebia cupida|AMIH023-08|1715990382|United States|BOLD:AAA7018|HQ152205
Leptophlebia cupida|AMIH077-08|4215980445|United States|BOLD:AAA7018|HQ152199
Leptophlebia cupida|AMIH205-08|8715990216|United States|BOLD:AAA7018|HQ152207
Leptophlebia cupida|AMIH027-08|1915980668|United States|BOLD:AAA7018|HQ152197
Leptophlebia cupida|AMIG036-08|2714180149|United States|BOLD:AAA7018|HQ152031
Leptophlebia cupida|AMIG007-08|1414180498|United States|BOLD:AAA7018|HQ152038
Leptophlebia cupida|AMIH022-08|1715981049|United States|BOLD:AAA7018|HQ152196
Leptophlebia cupida|AMIH114-08|5415980214|United States|BOLD:AAA7018|HQ152201
Leptophlebia cupida|AMIH157-08|7015980125|United States|BOLD:AAA7018|HQ152202
Leptophlebia cupida|AMIG142-08|8114010295|United States|BOLD:AAA7018|HQ152021
Leptophlebia cupida|AMIJ099-08|6919140176|United States|BOLD:AAA7018|HQ152593
Leptophlebia cupida|AMIG207-08|8814070163|United States|BOLD:AAA7018|HQ152023
Leptophlebia cupida|AMIG193-08|3314180958|United States|BOLD:AAA7018|HQ152053
Leptophlebia cupida|AMIG096-08|5614180748|United States|BOLD:AAA7018|HQ152042
Leptophlebia cupida|AMIG113-08|6314180580|United States|BOLD:AAA7018|HQ152045
Leptophlebia cupida|AMIH068-08|3915980399|United States|BOLD:AAA7018|HQ152198
Leptophlebia cupida|AMIG070-08|4214180672|United States|BOLD:AAA7018|HQ152036
Leptophlebia cupida|AMIH195-08|8316000156|United States|BOLD:AAA7018|HQ152206
Leptophlebia cupida|AMIG148-08|8514120129|United States|BOLD:AAA7018|HQ152048
Leptophlebia cupida|AMIG263-08|2214181028|United States|BOLD:AAA7018|HQ152055
Leptophlebia cupida|AMIG024-08|2214110171|United States|BOLD:AAA7018|HQ152019
Leptophlebia cupida|AMIG149-08|8514230118|United States|BOLD:AAA7018|HQ152061
Leptophlebia cupida|AMIG043-08|3114180350|United States|BOLD:AAA7018|HQ152032
Leptophlebia cupida|AMIG056-08|3614170139|United States|BOLD:AAA7018|HQ152018
Leptophlebia cupida|AMIG126-08|7214040261|United States|BOLD:AAA7018|HQ152047
Leptophlebia cupida|SWAMI166-11|SWA_iBOL_UofS_83|Canada|BOLD:AAA7018|JQ662679
Leptophlebia cupida|EPHNB063-09|09NBMAY-0063|Canada|BOLD:AAC0324|JQ663100
Leptophlebia cupida|EPHNB072-09|09NBMAY-0072|Canada|BOLD:AAC0324|JQ663356
Leptophlebia cupida|SWAMI164-11|SWA_iBOL_UofS_81|Canada|BOLD:AAC0324|JQ661857
Leptophlebia cupida|EPHNB077-09|09NBMAY-0077|Canada|BOLD:AAC0324|JQ661867
Leptophlebia cupida|EPHNB058-09|09NBMAY-0058|Canada|BOLD:AAC0324|JQ661818
Leptophlebia cupida|EPHNB061-09|09NBMAY-0061|Canada|BOLD:AAC0324|JQ662541
Leptophlebia cupida|EPHNB082-09|09NBMAY-0082|Canada|BOLD:AAC0324|JQ662658
Leptophlebia cupida|EPHNB080-09|09NBMAY-0080|Canada|BOLD:AAC0324|JQ662955
Leptophlebia cupida|CUMAY025-09|09CUMAY-025|Canada|BOLD:AAC0324|HM431527
Leptophlebia cupida|SWAMI163-11|SWA_iBOL_UofS_80|Canada|BOLD:AAC0324|JQ662743
Leptophlebia cupida|EPHNB081-09|09NBMAY-0081|Canada|BOLD:AAC0324|JQ661929
Leptophlebia cupida|EPHNB083-09|09NBMAY-0083|Canada|BOLD:AAC0324|JQ662446

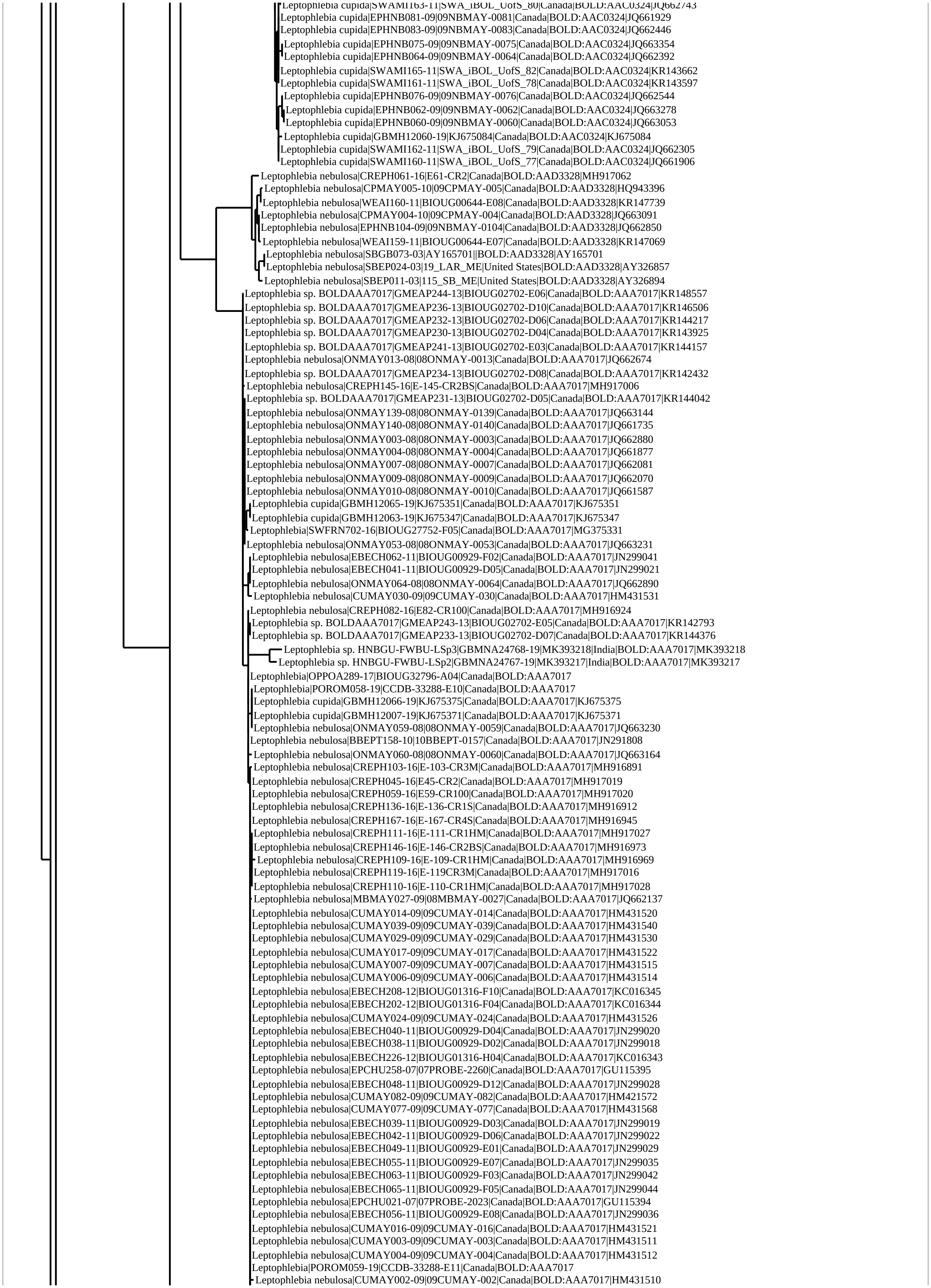
Leptophlebia cupida|SWAMI163-11|SWA_iBOL_UofS_80|Canada|BOLD:AAC0324|JQ662743
Leptophlebia cupida|EPHNB081-09|09NBMAY-0081|Canada|BOLD:AAC0324|JQ661929
Leptophlebia cupida|EPHNB083-09|09NBMAY-0083|Canada|BOLD:AAC0324|JQ662446
Leptophlebia cupida|EPHNB075-09|09NBMAY-0075|Canada|BOLD:AAC0324|JQ663354
Leptophlebia cupida|EPHNB064-09|09NBMAY-0064|Canada|BOLD:AAC0324|JQ662392
Leptophlebia cupida|SWAMI165-11|SWA_iBOL_UofS_82|Canada|BOLD:AAC0324|KR143662
Leptophlebia cupida|SWAMI161-11|SWA_iBOL_UofS_78|Canada|BOLD:AAC0324|KR143597
Leptophlebia cupida|EPHNB076-09|09NBMAY-0076|Canada|BOLD:AAC0324|JQ662544
Leptophlebia cupida|EPHNB062-09|09NBMAY-0062|Canada|BOLD:AAC0324|JQ663278
Leptophlebia cupida|EPHNB060-09|09NBMAY-0060|Canada|BOLD:AAC0324|JQ663053
Leptophlebia cupida|GBMH12060-19|KJ675084|Canada|BOLD:AAC0324|KJ675084
Leptophlebia cupida|SWAMI162-11|SWA_iBOL_UofS_79|Canada|BOLD:AAC0324|JQ662305
Leptophlebia cupida|SWAMI160-11|SWA_iBOL_UofS_77|Canada|BOLD:AAC0324|JQ661906
Leptophlebia nebulosa|CREPH061-16|E61-CR2|Canada|BOLD:AAD3328|MH917062
Leptophlebia nebulosa|CPMAY005-10|09CPMAY-005|Canada|BOLD:AAD3328|HQ943396
Leptophlebia nebulosa|WEAI160-11|BIOUG00644-E08|Canada|BOLD:AAD3328|KR147739
Leptophlebia nebulosa|CPMAY004-10|09CPMAY-004|Canada|BOLD:AAD3328|JQ663091
Leptophlebia nebulosa|EPHNB104-09|09NBMAY-0104|Canada|BOLD:AAD3328|JQ662850
Leptophlebia nebulosa|WEAI159-11|BIOUG00644-E07|Canada|BOLD:AAD3328|KR147069
Leptophlebia nebulosa|SBGB073-03|AY165701||BOLD:AAD3328|AY165701
Leptophlebia nebulosa|SBEP024-03|19_LAR_ME|United States|BOLD:AAD3328|AY326857
Leptophlebia nebulosa|SBEP011-03|115_SB_ME|United States|BOLD:AAD3328|AY326894
Leptophlebia sp. BOLDAAA7017|GMEAP244-13|BIOUG02702-E06|Canada|BOLD:AAA7017|KR148557
Leptophlebia sp. BOLDAAA7017|GMEAP236-13|BIOUG02702-D10|Canada|BOLD:AAA7017|KR146506
Leptophlebia sp. BOLDAAA7017|GMEAP232-13|BIOUG02702-D06|Canada|BOLD:AAA7017|KR144217
Leptophlebia sp. BOLDAAA7017|GMEAP230-13|BIOUG02702-D04|Canada|BOLD:AAA7017|KR143925
Leptophlebia sp. BOLDAAA7017|GMEAP241-13|BIOUG02702-E03|Canada|BOLD:AAA7017|KR144157
Leptophlebia nebulosa|ONMAY013-08|08ONMAY-0013|Canada|BOLD:AAA7017|JQ662674
Leptophlebia sp. BOLDAAA7017|GMEAP234-13|BIOUG02702-D08|Canada|BOLD:AAA7017|KR142432
Leptophlebia nebulosa|CREPH145-16|E-145-CR2BS|Canada|BOLD:AAA7017|MH917006
Leptophlebia sp. BOLDAAA7017|GMEAP231-13|BIOUG02702-D05|Canada|BOLD:AAA7017|KR144042
Leptophlebia nebulosa|ONMAY139-08|08ONMAY-0139|Canada|BOLD:AAA7017|JQ663144
Leptophlebia nebulosa|ONMAY140-08|08ONMAY-0140|Canada|BOLD:AAA7017|JQ661735
Leptophlebia nebulosa|ONMAY003-08|08ONMAY-0003|Canada|BOLD:AAA7017|JQ662880
Leptophlebia nebulosa|ONMAY004-08|08ONMAY-0004|Canada|BOLD:AAA7017|JQ661877
Leptophlebia nebulosa|ONMAY007-08|08ONMAY-0007|Canada|BOLD:AAA7017|JQ662081
Leptophlebia nebulosa|ONMAY009-08|08ONMAY-0009|Canada|BOLD:AAA7017|JQ662070
Leptophlebia nebulosa|ONMAY010-08|08ONMAY-0010|Canada|BOLD:AAA7017|JQ661587
Leptophlebia cupida|GBMH12065-19|KJ675351|Canada|BOLD:AAA7017|KJ675351
Leptophlebia cupida|GBMH12063-19|KJ675347|Canada|BOLD:AAA7017|KJ675347
Leptophlebia|SWFRN702-16|BIOUG27752-F05|Canada|BOLD:AAA7017|MG375331
Leptophlebia nebulosa|ONMAY053-08|08ONMAY-0053|Canada|BOLD:AAA7017|JQ663231
Leptophlebia nebulosa|EBECH062-11|BIOUG00929-F02|Canada|BOLD:AAA7017|JN299041
Leptophlebia nebulosa|EBECH041-11|BIOUG00929-D05|Canada|BOLD:AAA7017|JN299021
Leptophlebia nebulosa|ONMAY064-08|08ONMAY-0064|Canada|BOLD:AAA7017|JQ662890
Leptophlebia nebulosa|CUMAY030-09|09CUMAY-030|Canada|BOLD:AAA7017|HM431531
Leptophlebia nebulosa|CREPH082-16|E82-CR100|Canada|BOLD:AAA7017|MH916924
Leptophlebia sp. BOLDAAA7017|GMEAP243-13|BIOUG02702-E05|Canada|BOLD:AAA7017|KR142793
Leptophlebia sp. BOLDAAA7017|GMEAP233-13|BIOUG02702-D07|Canada|BOLD:AAA7017|KR144376
Leptophlebia sp. HNBGU-FWBU-LSp3|GBMNA24768-19|MK393218|India|BOLD:AAA7017|MK393218
Leptophlebia sp. HNBGU-FWBU-LSp2|GBMNA24767-19|MK393217|India|BOLD:AAA7017|MK393217
Leptophlebia|OPPOA289-17|BIOUG32796-A04|Canada|BOLD:AAA7017
Leptophlebia|POROM058-19|CCDB-33288-E10|Canada|BOLD:AAA7017
Leptophlebia cupida|GBMH12066-19|KJ675375|Canada|BOLD:AAA7017|KJ675375
Leptophlebia cupida|GBMH12007-19|KJ675371|Canada|BOLD:AAA7017|KJ675371
Leptophlebia nebulosa|ONMAY059-08|08ONMAY-0059|Canada|BOLD:AAA7017|JQ663230
Leptophlebia nebulosa|BBEPT158-10|10BBEPT-0157|Canada|BOLD:AAA7017|JN291808
Leptophlebia nebulosa|ONMAY060-08|08ONMAY-0060|Canada|BOLD:AAA7017|JQ663164
Leptophlebia nebulosa|CREPH103-16|E-103-CR3M|Canada|BOLD:AAA7017|MH916891
Leptophlebia nebulosa|CREPH045-16|E45-CR2|Canada|BOLD:AAA7017|MH917019
Leptophlebia nebulosa|CREPH059-16|E59-CR100|Canada|BOLD:AAA7017|MH917020
Leptophlebia nebulosa|CREPH136-16|E-136-CR1S|Canada|BOLD:AAA7017|MH916912
Leptophlebia nebulosa|CREPH167-16|E-167-CR4S|Canada|BOLD:AAA7017|MH916945
Leptophlebia nebulosa|CREPH111-16|E-111-CR1HM|Canada|BOLD:AAA7017|MH917027
Leptophlebia nebulosa|CREPH146-16|E-146-CR2BS|Canada|BOLD:AAA7017|MH916973
Leptophlebia nebulosa|CREPH109-16|E-109-CR1HM|Canada|BOLD:AAA7017|MH916969
Leptophlebia nebulosa|CREPH119-16|E-119CR3M|Canada|BOLD:AAA7017|MH917016
Leptophlebia nebulosa|CREPH110-16|E-110-CR1HM|Canada|BOLD:AAA7017|MH917028
Leptophlebia nebulosa|MBMAY027-09|08MBMAY-0027|Canada|BOLD:AAA7017|JQ662137
Leptophlebia nebulosa|CUMAY014-09|09CUMAY-014|Canada|BOLD:AAA7017|HM431520
Leptophlebia nebulosa|CUMAY039-09|09CUMAY-039|Canada|BOLD:AAA7017|HM431540
Leptophlebia nebulosa|CUMAY029-09|09CUMAY-029|Canada|BOLD:AAA7017|HM431530
Leptophlebia nebulosa|CUMAY017-09|09CUMAY-017|Canada|BOLD:AAA7017|HM431522
Leptophlebia nebulosa|CUMAY007-09|09CUMAY-007|Canada|BOLD:AAA7017|HM431515
Leptophlebia nebulosa|CUMAY006-09|09CUMAY-006|Canada|BOLD:AAA7017|HM431514
Leptophlebia nebulosa|EBECH208-12|BIOUG01316-F10|Canada|BOLD:AAA7017|KC016345
Leptophlebia nebulosa|EBECH202-12|BIOUG01316-F04|Canada|BOLD:AAA7017|KC016344
Leptophlebia nebulosa|CUMAY024-09|09CUMAY-024|Canada|BOLD:AAA7017|HM431526
Leptophlebia nebulosa|EBECH040-11|BIOUG00929-D04|Canada|BOLD:AAA7017|JN299020
Leptophlebia nebulosa|EBECH038-11|BIOUG00929-D02|Canada|BOLD:AAA7017|JN299018
Leptophlebia nebulosa|EBECH226-12|BIOUG01316-H04|Canada|BOLD:AAA7017|KC016343
Leptophlebia nebulosa|EPCHU258-07|07PROBE-2260|Canada|BOLD:AAA7017|GU115395
Leptophlebia nebulosa|EBECH048-11|BIOUG00929-D12|Canada|BOLD:AAA7017|JN299028
Leptophlebia nebulosa|CUMAY082-09|09CUMAY-082|Canada|BOLD:AAA7017|HM421572
Leptophlebia nebulosa|CUMAY077-09|09CUMAY-077|Canada|BOLD:AAA7017|HM431568
Leptophlebia nebulosa|EBECH039-11|BIOUG00929-D03|Canada|BOLD:AAA7017|JN299019
Leptophlebia nebulosa|EBECH042-11|BIOUG00929-D06|Canada|BOLD:AAA7017|JN299022
Leptophlebia nebulosa|EBECH049-11|BIOUG00929-E01|Canada|BOLD:AAA7017|JN299029
Leptophlebia nebulosa|EBECH055-11|BIOUG00929-E07|Canada|BOLD:AAA7017|JN299035
Leptophlebia nebulosa|EBECH063-11|BIOUG00929-F03|Canada|BOLD:AAA7017|JN299042
Leptophlebia nebulosa|EBECH065-11|BIOUG00929-F05|Canada|BOLD:AAA7017|JN299044
Leptophlebia nebulosa|EPCHU021-07|07PROBE-2023|Canada|BOLD:AAA7017|GU115394
Leptophlebia nebulosa|EBECH056-11|BIOUG00929-E08|Canada|BOLD:AAA7017|JN299036
Leptophlebia nebulosa|CUMAY016-09|09CUMAY-016|Canada|BOLD:AAA7017|HM431521
Leptophlebia nebulosa|CUMAY003-09|09CUMAY-003|Canada|BOLD:AAA7017|HM431511
Leptophlebia nebulosa|CUMAY004-09|09CUMAY-004|Canada|BOLD:AAA7017|HM431512
Leptophlebia|POROM059-19|CCDB-33288-E11|Canada|BOLD:AAA7017
Leptophlebia nebulosa|CUMAY002-09|09CUMAY-002|Canada|BOLD:AAA7017|HM431510

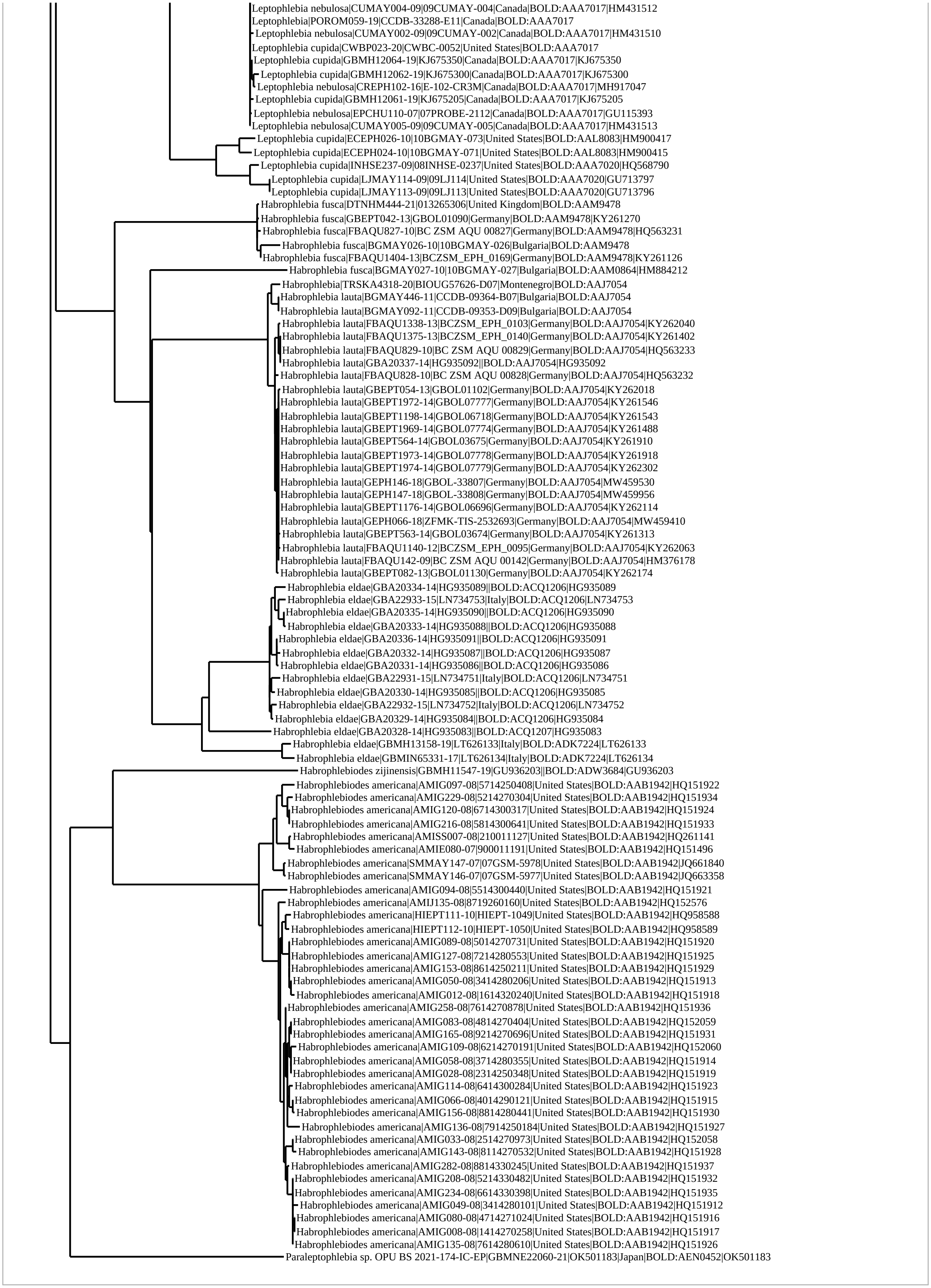
Leptophlebia nebulosa|CUMAY004-09|09CUMAY-004|Canada|BOLD:AAA7017|HM431512
Leptophlebia|POROM059-19|CCDB-33288-E11|Canada|BOLD:AAA7017
Leptophlebia nebulosa|CUMAY002-09|09CUMAY-002|Canada|BOLD:AAA7017|HM431510
Leptophlebia cupida|CWBP023-20|CWBC-0052|United States|BOLD:AAA7017
Leptophlebia cupida|GBMH12064-19|KJ675350|Canada|BOLD:AAA7017|KJ675350
Leptophlebia cupida|GBMH12062-19|KJ675300|Canada|BOLD:AAA7017|KJ675300
Leptophlebia nebulosa|CREPH102-16|E-102-CR3M|Canada|BOLD:AAA7017|MH917047
Leptophlebia cupida|GBMH12061-19|KJ675205|Canada|BOLD:AAA7017|KJ675205
Leptophlebia nebulosa|EPCHU110-07|07PROBE-2112|Canada|BOLD:AAA7017|GU115393
Leptophlebia nebulosa|CUMAY005-09|09CUMAY-005|Canada|BOLD:AAA7017|HM431513
Leptophlebia cupida|ECEPH026-10|10BGMAY-073|United States|BOLD:AAL8083|HM900417
Leptophlebia cupida|ECEPH024-10|10BGMAY-071|United States|BOLD:AAL8083|HM900415
Leptophlebia cupida|INHSE237-09|08INHSE-0237|United States|BOLD:AAA7020|HQ568790
Leptophlebia cupida|LJMAY114-09|09LJ114|United States|BOLD:AAA7020|GU713797
Leptophlebia cupida|LJMAY113-09|09LJ113|United States|BOLD:AAA7020|GU713796
Habrophlebia fusca|DTNHM444-21|013265306|United Kingdom|BOLD:AAM9478
Habrophlebia fusca|GBEPT042-13|GBOL01090|Germany|BOLD:AAM9478|KY261270
Habrophlebia fusca|FBAQU827-10|BC ZSM AQU 00827|Germany|BOLD:AAM9478|HQ563231
Habrophlebia fusca|BGMAY026-10|10BGMAY-026|Bulgaria|BOLD:AAM9478
Habrophlebia fusca|FBAQU1404-13|BCZSM_EPH_0169|Germany|BOLD:AAM9478|KY261126
Habrophlebia fusca|BGMAY027-10|10BGMAY-027|Bulgaria|BOLD:AAM0864|HM884212
Habrophlebia|TRSKA4318-20|BIOUG57626-D07|Montenegro|BOLD:AAJ7054
Habrophlebia lauta|BGMAY446-11|CCDB-09364-B07|Bulgaria|BOLD:AAJ7054
Habrophlebia lauta|BGMAY092-11|CCDB-09353-D09|Bulgaria|BOLD:AAJ7054
Habrophlebia lauta|FBAQU1338-13|BCZSM_EPH_0103|Germany|BOLD:AAJ7054|KY262040
Habrophlebia lauta|FBAQU1375-13|BCZSM_EPH_0140|Germany|BOLD:AAJ7054|KY261402
Habrophlebia lauta|FBAQU829-10|BC ZSM AQU 00829|Germany|BOLD:AAJ7054|HQ563233
Habrophlebia lauta|GBA20337-14|HG935092||BOLD:AAJ7054|HG935092
Habrophlebia lauta|FBAQU828-10|BC ZSM AQU 00828|Germany|BOLD:AAJ7054|HQ563232
Habrophlebia lauta|GBEPT054-13|GBOL01102|Germany|BOLD:AAJ7054|KY262018
Habrophlebia lauta|GBEPT1972-14|GBOL07777|Germany|BOLD:AAJ7054|KY261546
Habrophlebia lauta|GBEPT1198-14|GBOL06718|Germany|BOLD:AAJ7054|KY261543
Habrophlebia lauta|GBEPT1969-14|GBOL07774|Germany|BOLD:AAJ7054|KY261488
Habrophlebia lauta|GBEPT564-14|GBOL03675|Germany|BOLD:AAJ7054|KY261910
Habrophlebia lauta|GBEPT1973-14|GBOL07778|Germany|BOLD:AAJ7054|KY261918
Habrophlebia lauta|GBEPT1974-14|GBOL07779|Germany|BOLD:AAJ7054|KY262302
Habrophlebia lauta|GEPH146-18|GBOL-33807|Germany|BOLD:AAJ7054|MW459530
Habrophlebia lauta|GEPH147-18|GBOL-33808|Germany|BOLD:AAJ7054|MW459956
Habrophlebia lauta|GBEPT1176-14|GBOL06696|Germany|BOLD:AAJ7054|KY262114
Habrophlebia lauta|GEPH066-18|ZFMK-TIS-2532693|Germany|BOLD:AAJ7054|MW459410
Habrophlebia lauta|GBEPT563-14|GBOL03674|Germany|BOLD:AAJ7054|KY261313
Habrophlebia lauta|FBAQU1140-12|BCZSM_EPH_0095|Germany|BOLD:AAJ7054|KY262063
Habrophlebia lauta|FBAQU142-09|BC ZSM AQU 00142|Germany|BOLD:AAJ7054|HM376178
Habrophlebia lauta|GBEPT082-13|GBOL01130|Germany|BOLD:AAJ7054|KY262174
Habrophlebia eldae|GBA20334-14|HG935089||BOLD:ACQ1206|HG935089
Habrophlebia eldae|GBA22933-15|LN734753|Italy|BOLD:ACQ1206|LN734753
Habrophlebia eldae|GBA20335-14|HG935090||BOLD:ACQ1206|HG935090
Habrophlebia eldae|GBA20333-14|HG935088||BOLD:ACQ1206|HG935088
Habrophlebia eldae|GBA20336-14|HG935091||BOLD:ACQ1206|HG935091
Habrophlebia eldae|GBA20332-14|HG935087||BOLD:ACQ1206|HG935087
Habrophlebia eldae|GBA20331-14|HG935086||BOLD:ACQ1206|HG935086
Habrophlebia eldae|GBA22931-15|LN734751|Italy|BOLD:ACQ1206|LN734751
Habrophlebia eldae|GBA20330-14|HG935085||BOLD:ACQ1206|HG935085
Habrophlebia eldae|GBA22932-15|LN734752|Italy|BOLD:ACQ1206|LN734752
Habrophlebia eldae|GBA20329-14|HG935084||BOLD:ACQ1206|HG935084
Habrophlebia eldae|GBA20328-14|HG935083||BOLD:ACQ1207|HG935083
Habrophlebia eldae|GBMH13158-19|LT626133|Italy|BOLD:ADK7224|LT626133
Habrophlebia eldae|GBMIN65331-17|LT626134|Italy|BOLD:ADK7224|LT626134
Habrophlebiodes zijinensis|GBMH11547-19|GU936203||BOLD:ADW3684|GU936203
Habrophlebiodes americana|AMIG097-08|5714250408|United States|BOLD:AAB1942|HQ151922
Habrophlebiodes americana|AMIG229-08|5214270304|United States|BOLD:AAB1942|HQ151934
Habrophlebiodes americana|AMIG120-08|6714300317|United States|BOLD:AAB1942|HQ151924
Habrophlebiodes americana|AMIG216-08|5814300641|United States|BOLD:AAB1942|HQ151933
Habrophlebiodes americana|AMISS007-08|210011127|United States|BOLD:AAB1942|HQ261141
Habrophlebiodes americana|AMIE080-07|900011191|United States|BOLD:AAB1942|HQ151496
Habrophlebiodes americana|SMMAY147-07|07GSM-5978|United States|BOLD:AAB1942|JQ661840
Habrophlebiodes americana|SMMAY146-07|07GSM-5977|United States|BOLD:AAB1942|JQ663358
Habrophlebiodes americana|AMIG094-08|5514300440|United States|BOLD:AAB1942|HQ151921
Habrophlebiodes americana|AMIJ135-08|8719260160|United States|BOLD:AAB1942|HQ152576
Habrophlebiodes americana|HIEPT111-10|HIEPT-1049|United States|BOLD:AAB1942|HQ958588
Habrophlebiodes americana|HIEPT112-10|HIEPT-1050|United States|BOLD:AAB1942|HQ958589
Habrophlebiodes americana|AMIG089-08|5014270731|United States|BOLD:AAB1942|HQ151920
Habrophlebiodes americana|AMIG127-08|7214280553|United States|BOLD:AAB1942|HQ151925
Habrophlebiodes americana|AMIG153-08|8614250211|United States|BOLD:AAB1942|HQ151929
Habrophlebiodes americana|AMIG050-08|3414280206|United States|BOLD:AAB1942|HQ151913
Habrophlebiodes americana|AMIG012-08|1614320240|United States|BOLD:AAB1942|HQ151918
Habrophlebiodes americana|AMIG258-08|7614270878|United States|BOLD:AAB1942|HQ151936
Habrophlebiodes americana|AMIG083-08|4814270404|United States|BOLD:AAB1942|HQ152059
Habrophlebiodes americana|AMIG165-08|9214270696|United States|BOLD:AAB1942|HQ151931
Habrophlebiodes americana|AMIG109-08|6214270191|United States|BOLD:AAB1942|HQ152060
Habrophlebiodes americana|AMIG058-08|3714280355|United States|BOLD:AAB1942|HQ151914
Habrophlebiodes americana|AMIG028-08|2314250348|United States|BOLD:AAB1942|HQ151919
Habrophlebiodes americana|AMIG114-08|6414300284|United States|BOLD:AAB1942|HQ151923
Habrophlebiodes americana|AMIG066-08|4014290121|United States|BOLD:AAB1942|HQ151915
Habrophlebiodes americana|AMIG156-08|8814280441|United States|BOLD:AAB1942|HQ151930
Habrophlebiodes americana|AMIG136-08|7914250184|United States|BOLD:AAB1942|HQ151927
Habrophlebiodes americana|AMIG033-08|2514270973|United States|BOLD:AAB1942|HQ152058
Habrophlebiodes americana|AMIG143-08|8114270532|United States|BOLD:AAB1942|HQ151928
Habrophlebiodes americana|AMIG282-08|8814330245|United States|BOLD:AAB1942|HQ151937
Habrophlebiodes americana|AMIG208-08|5214330482|United States|BOLD:AAB1942|HQ151932
Habrophlebiodes americana|AMIG234-08|6614330398|United States|BOLD:AAB1942|HQ151935
Habrophlebiodes americana|AMIG049-08|3414280101|United States|BOLD:AAB1942|HQ151912
Habrophlebiodes americana|AMIG080-08|4714271024|United States|BOLD:AAB1942|HQ151916
Habrophlebiodes americana|AMIG008-08|1414270258|United States|BOLD:AAB1942|HQ151917
Habrophlebiodes americana|AMIG135-08|7614280610|United States|BOLD:AAB1942|HQ151926
Paraleptophlebia sp. OPU BS 2021-174-IC-EP|GBMNE22060-21|OK501183|Japan|BOLD:AEN0452|OK501183
