# Supplementary material for: Cenozoic origins of the genus Calliarcys (Insecta, Ephemeroptera) revealed by Micro-CT, with DNA barcode gap analysis of Leptophlebiinae and Habrophlebiinae
Source: Sci Rep. 2022 Sep 8;12:15228. doi: 10.1038/s41598-022-18234-4 (PMC9458648; doi:10.1038/s41598-022-18234-4)
Supplement: Supplementary file 7 — Supplementary Information 5. [file 41598_2022_18234_MOESM7_ESM.docx]

**Supplementary material S5**

Short overview of Eocene Leptophlebiidae,

with remarks on the earliest and latest fossil records of this family

*Supplementary information to:* Cenozoic origins of the genus Calliarcys (Insecta, Ephemeroptera) revealed by Micro-CT, and DNA barcode gap analysis of Leptophlebiinae and Habrophlebiinae

*Roman J. Godunko, Javier Alba-Tercedor, Michal Grabowski, Tomasz Rewicz, Arnold H. Staniczek*

The oldest confirmed record of Eocene Leptophlebiidae is the genus *Paraleptophlebia* recorded from Eocene Baltic amber. Together with representatives of the genus *Siphloplecton* (Metretopodidae), *Paraleptophlebia prisca* (Pictet & Hagen, 1856) was one of the first fossil mayfly species described in the middle of XIX century from the amber collection housed at the Museum für Naturkunde Berlin [1; 2; 3]. Demoulin [2, 4] for the last time investigated the type specimen, but today the male imago of the “hololectotype” has to be regarded as lost, despite of thorough search in the MNB amber collection (for more details see [3], so a current analysis of its systematic position is impossible. The available lectotype of *P. prisca* is represented by a male imago without tip of abdomen and without preserved genitalia [2; 5]. As a consequence, the comparison with other Eocene material is rather difficult.

Kluge [5] established a second extinct species of *Paraleptophlebia*, i.e., *Paraleptophlebia electra* (Kluge, 1993) for a male imago in Baltic amber, initially placed in the genus *Leptophlebia* Westwood, 1840. The discussion on its taxonomic placement and its affinities was grounded on comparisons with specimens of *Paraleptophlebia* outside of the type series of *P. prisca* [4; 6], e.g. the types of two species of *Oligophlebia* Demoulin, 1965, established for adults from Baltic amber and later synonymized with *Paraleptophlebia,* as well as several alate individuals of imago and subimago of both sexes marked as *P. prisca* by Demoulin [4].

These shortcomings demonstrate the necessity to search for the lectotype of *P. prisca* and the reinvestigated type series of *Oligophlebia calliarcys* Demoulin, 1965 and *O. longiceps* Demoulin, 1965, in order to assess the diagnostic characters of all recorded Eocene Leptophlebiidae (see Table S3).

It is also essential in the case of the new material, which has been discovered, for instance specimens recently recorded from Baltic amber, which can be associated with *P. electra*, and most probably with *P. prisca* (Fig. S1). Numerous specimens of the genus *Paraleptophlebia* were also found in Late Eocene Rovno amber (Fig. S2). Additionally, a few specimens of *Paraleptophlebia* in Baltic amber may also represent new species (Fig. S2).

According to Hubbard & Savage [7], also two genera of Atalophlebiinae were described based on the Eocene fossil remnants:

**(I)** The genus *Xenophlebia* Demoulin, 1968 was established for a single species *X. aenigmatica* Demoulin, 1968 based on male imago from Eocene Baltic amber [2]. Based on characters of the wings venation and genitalia, this taxon was initially placed within Atalophlebiinae. However, the presence of hexagonal facets in the compound eyes of the holotype excludes such a placement and would only permit a relationship of *Xenophlebia* within Leptophlebiinae–Habrophlebiinae. However, the presence of **(a)** compound eyes with hexagonal facets, **(b)** the forewing cubital field with free elongated intercalaries, **(c)** the well-developed costal process of hind wings, **(d)** the mesosternum with contiguous furcasternal protuberances and **(e)** 5-segmented tarsi of the middle and hind legs, with the segment I fused to tibia (tarsal claws are dissimilar on all legs), rather indicate a systematic position of *Xenophlebia* in Siphlonuroidea, or even a closer relationship to Metretopodidae, which are rather diverse in Baltic amber [3; 8; 9] (Fig. S3). Thus, we exclude *Xenophlebia* from Leptophlebiidae and provisionally transfer it to Siphlonuroidea. The question of relationships between *Xenophlebia* and fossil Metretopodidae (*Metretopus* and *Siphloplecton*) should be postponed until the detailed reinvestigation of the holotype of *X. aenigmatica*.

**(II)** The monotypic genus *Blasturophlebia* Demoulin, 1968 was established for a single male subimaginal exuvia described under the name *B. hirsuta* Demoulin, 1968 from the Eocene Baltic amber, and placed originally in Leptophlebiidae. Hubbard & Savage [7], based on original description and drawings published by Demoulin [2], assumed that *Blasturophlebia* belongs to Atalophlebiinae, if they should have four-segmented foreleg tarsi. In fact, foretarsi of *Blasturophlebia* are five-segmented, with tarsomere I distinctly shortened and fused with tibia, and dissimilar tarsal claws [with those of Atalophlebiinae] (Fig. S4). In combination with inaccessibility of the information on wings venation, we are convinced that the disputed taxon should be transferred with reasonable care to Furcatergaliae *incertae sedis*, as also defined by Kluge [10].

We avoid here the discussion about the fossil female subimaginal exuvia determined as *Choroterpes* sp. by Demoulin [2]. Following Hubbard & Savage [7], due to scarce of characters useful to determine the systematic position, we see no reason in assigning this specimen to neither *Choroterpes,* nor Leptophlebiidae in general. Thus, we consider it Ephemeroptera *incertae sedis*.

The earliest reliable record of the subfamily Leptophlebiinae is a female subimago of the monotypic genus *Aureophlebia sinitshenkovae* Peters & Peters, 2000, reported from the Upper Cretaceous New Jersey amber, with an approximate age of 100 mya [11]. The placement of *A. sinitshenkovae* in Leptophlebiinae was established based on the MA furcation, with straight MA1 and strongly symmetrical MA2 (the same character was also reported for *Habrophlebiodes* and closely related genera). On the other hand, a similar MA fork is found in some Neotropical members of Atalophlebiinae. Because the shape of the facets in the compound eye have not been reported in the original description of *A. sinitshenkovae*, another character fitting the extinct species closer to Leptophlebiinae is the presence of an inconspicuous, apically broadly rounded costal process of hind wings, depicted by Peters & Peters [11].

The youngest fossil record of Leptophlebiinae is dated from the Middle Miocene of China. Zhang [12] reported a nymph of *Paraleptophlebia* in the [Miocene](https://en.wikipedia.org/wiki/Miocene) Shanwang Formation. While the discussed specimen is quite similar to extant Leptophlebiinae, other nymphal specimens described and attributed to the genus *Heptagenia* Walsh, 1853 (e.g., *H. shanwangensis* (Hong, 1983), and *H. fluminea* Zhang, 1989; both currently in Heptageniidae Needcham, 1901), most probably belong to Choroterpini within Leptophlebiidae. Our assumption is based on several characters, namely the structure of head (prognathous and flattened dorsoventrally, with presumably flattened and widened mandibles; putative remnants of mandibles with a convex outer margin), and the shape of gills. The preserved remnants of putative gills are leaf-shaped, convergent apically, with the lamella terminated by several poorly distinguishable processes (see in Zhang [12] the plates 1.1 and 1.2 and compare with figures 4, 6 in the same contribution). Interestingly, the species *H. shanwangensis* was initially described within the genus *Ephemerella* Walsh, 1862 (see Hong [13]), and only later transferred to *Heptagenia* by Zhang [12].

The discussion about the trustworthy earliest record of the subfamily Atalophlebiinae is open. McCafferty [14] described the Lower Cretaceous *Conovirilus poinari* from an alate specimen in Lebanese amber , dated 120–135 mya, as a member of Atalophlebiinae. However, the systematic position of this species should be clarified based on a reinvestigation of the holotype, because no information is available on the shape of compound eyes facets and features of wing venation in the original description. Nevertheless, Peters & Peters [11] assumed close relations of *C. poinari* with the *Terpides* lineage sensu Savage [15] and Peters [16] (Terpidinae sensu Monjardim et al. [17]), provided it is a true Atalophlebiinae, based on the shape of penis and similar claws, in contrast to McCafferty’s [14] conclusion about its similarities to *Atalophlebioides* lineage.

The designation of nymphal records of Atalophlebiinae, as well as Leptophlebiidae, is even more problematic due to poor preservation of fossil remnants associated with this family. The oldest nymphal findings of fossil representatives listed as Leptophlebiidae are reported from the Middle and Upper Jurassic of the Russian Federation and China with the genus *Mesoneta* Brauer et al., 1889. Altogether fourteen species were described (see N.J. Kluge web-page: <http://insecta.bio.spbu.ru/z/Eph-spp/))Mesoneta.htm>; June, 2022). The genus *Mesoneta*, as well as two other Mesozoic genera, *Ninadsa* Özdikmen, 2008 (originally described as *Leptoneta* Sinitshenkova, 1989 *nom. preocc.*; Lower Cretaceous of Mongolia) and *Cretoneta* Tshernova, 1971 (Upper Cretaceous, Taimyr amber), were attributed to the subfamily Mesonetinae. Nevertheless, Kluge [5] transferred *Cretoneta* to Siphlonuridae. Finally, Zhang & Kluge [18] regards Mesonetinae as Euplectoptera *incertae sedis* due to insufficient data on distinguishing characters of the fossil adults and larvae.

Three relatively well-preserved nymphs from the Crato Formation of Brazil (Aptian, Lower Cretaceous) were reported by Staniczek [19] under “Familia *incertae sedis*, gen. et sp. nov.” (see Fig. S5). Staniczek [19] commented this material as a “…strange mayfly larvae (body length about 23 mm), which have an absolutely unique habitus with broadened and flattened fore- and hind femora but slender mid femora”. A reinvestigation revealed that all three discovered larvae are characterized by (**a**) a flattened body; (**b**) a prognathous head flattened dorsoventrally; (**c**) remnants of tracheal gills on segments I–VII, which are bilamellate, leaf-shaped, pointed apically, with non-diminished pair VII (Fig. S5). Based on additional analysis, we provisionally place these nymphs to Leptophlebiidae. The presence of markedly flattened and broad fore- and hind femora, with the remnants of strong spines along of the forefemora inner margin in combination of the characters listed above, indicate an undoubtedly new mayfly genus (Staniczek et al., *in preparation*).

The youngest fossil Atalophlebiinae with clearly confirmed generic attribution are recorded from Miocene Dominican amber. Several species attributed to two genera were described: *Borinquena* Traver, 1938 initially established for extant mayfly species from the Caribbean Region, also contains four extinct species (*B. maculata* Staniczek, 2003*, B. parva* Staniczek, 2003*, B. caeciliana* Staniczek, 2003, and *B. schawallfussi* Staniczek et. al., 2017); and the monotypic genus *Hagenulites* Staniczek, 2003 with a single Miocene species *H. hitchingsi* Staniczek, 2003 [20; 21; 22].

Information about Leptophlebiidae in Mexican amber is markedly scarce. Solórzano Kraemer [23] shortly described and figured forewing and genitalia of a male imago and referred to it as “Leptophlebiidae (?)” from the collection of the Museum of Palaeontology in Tuxtla Gutiérrez Chiapas. Based on this information, we suppose the existence of a new undescribed species of Atalophlebiinae or Choroterpini sensu Monjardim et al. [17].

Finally, the latest fossil record of Atalophlebiinae is *Ephemera culleni* Etheridge & Olliff, 1890. It was described from nymphal material from the Pliocene of New South Wales (Australia), and later transferred to the genus *Atalophlebia* Eaton, 1881 by Riek [24].

**References**

1. Pictet-Baraban, F. J. & Hagen, H. Die im Bernstein befindlichen Neuropteren der Vorwelt. In *Die im Bernstein befindlichen organischen Reste der Vorwelt* (ed. Berendt, G. C.) Zweiter Band, II. Abtheilung, 41–126 (Nicolaische Buchhandlung, Berlin, 1856).

2. Demoulin, G. Deuxieme contibution à la connaissance des Ephéméroptères de l'ambre oligocene de la Baltique. *Deutsche Entomologische Zeitschrift* **15** (1–3), 233–276 (1968).

3. Staniczek, A. H. & Godunko, R. J. Revision of fossil Metretopodidae (Insecta: Ephemeroptera) in Baltic amber – Part 1: Type specimens of hitherto described fossil species of *Siphloplecton* Clemens, 1915 and *Metretopus* Eaton, 1901, with description of four new fossil species of *Siphloplecton*. *Palaeodiversity* **5**, 57–87 (2012).

4. Demoulin, G. Troisième contribution à la connaissance des Ephéméroptères de l'ambre oligocène de la Baltique. *Bulletin de l'Institut Royal des Sciences Naturelles de Belgique* **46** (2), 1–11 (1970).

5. Kluge, N. J. New data on mayflies (Ephemeroptera) from fossil Mesozoic and Cenozoic resins. *Palaeontological Journal* **27** (1A), 35–49 (1993).

6. Demoulin, G. Contribution à la connaissance des Ephéméroptères de l'ambre oligocene de la Baltique. *Entomologiske Meddelelser* **34**, 143–153 (1965).

7. Hubbard, M. D. & Savage H. M. The fossil Leptophlebiidae (Ephemeroptera): A systematic and phylogenetic review. *Journal of Paleontology* **55**, 810–813 (1981).

8. Staniczek, A. H. & Godunko, R. J. Revision of fossil Metretopodidae (Insecta: Ephemeroptera) in Baltic amber— Part 3: Description of two new species of *Siphloplecton* Clemens, 1915, with notes on the re-discovered lectotype of *Siphloplecton macrops* (Pictet-Baraban & Hagen, 1856) *Zootaxa* **4103**, 1–24 (2016).

9. Godunko, R. J., Neumann, C. & Staniczek, A. Revision of fossil Metretopodidae (Insecta, Ephemeroptera) in Baltic amber – Part 4: Description of two new species of *Siphloplecton* Clemens, 1915, with notes on the new *S. jaegeri* species group and with key to fossil male adults of Siphloplecton. *ZooKeys* **898**, 1–26 (2019).

10. Kluge, N. J. *The Phylogenetic System of Ephemeroptera* (Kluwer Academic Publishers, Dordrecht, 1–442 (2004).

11. Peters, W. L., Peters, J. G. Discovery of a new genus of Leptophlebiidae: Leptophlebiinae (Ephemeroptera) in Cretaceous amber from New Jersey. In *Studies on fossils in amber, with particular reference to the Cretaceous of New Jersey* (ed. Grimadi, D.) 127–131 (Backhuys Publ., Leiden, Netherlands, 2000).

12. Zhang, J.-F. *Fossil insects from Shanwang, Shandong, China* (Jinan: Shandong Science and Technology Publishing House, 1–459 (1989). [in Chinese with English summary]

13. Hong, Y.-Ch. *Middle Yrassic fossil insects in northern China* (Geologicl Publishing House, Peking, 1–223 (1983).

14. McCafferty, W. P. Discovery and analysis of the oldest mayflies (Insecta, Ephemeroptera) known from amber. *Bulletin de la Société d'histoire naturelle de Toulouse* **133**, 77–82 (1997).

15. Savage, H. M. Systematics of the *Terpides* lineage from the Neotropics: Definition of the *Terpides* lineage, methods, and revision of *Fittkaulus* Savage & Peters. *Spixiana* **9** (3), 255–270 (1986).

16. Peters, W. L. A redescription of the imago of *Castanophlebia* Barnard, 1932 from South Africa (Ephemeroptera: Leptophlebiidae: Atalophlebiinae). In *Ephemeroptera & Plecoptera. Biology-Ecology-Systematics. Proc. 8th Int. Conf. Ephemeroptera & 12th Int. Symp. Plecoptera, 14–20 August 1995, Lausanne, Switzerland* (eds Landolt, P. & Sartori, M.) 449–454 (Mauron+Tinguely & Lacht SA, Fribourg/Switzerland, 1997).

17. Monjardim, M., Paresque, R. & Salles, F. F. Phylogeny and classification of Leptophlebiidae (Ephemeroptera) with an emphasis on Neotropical fauna. *Systematic Entomology* **45** (2), 415–429 (2020).

18. Zhang, J.-F., & Kluge, N.J. Jurassic larvae of mayflies (Ephemeroptera) from the Daohugou Formation in Inner Mongolia, China. *Oriental Insects* **41**, 351–366 (2007).

19. Staniczek, A. H. Ephemeroptera: mayflies. In *The Crato fossil beds of Brazil: Window into an ancient world* (eds Martill, D. M. & Bechly, G.) 163–184 (Cambridge University Press, Cambridge, 2007).

20. Staniczek, A. H. New fossil mayflies from Dominican amber (Insecta: Ephemeroptera: Leptophlebiidae: Atalophlebiinae). *Stuttgarter Beiträge zur Naturkunde (Ser.B. Geologie und Paläontologie)* **341**, 1–22 (2003).

21. Godunko, R. J. & Krzemiński, W. New fossil findings of the mayfly genera *Balticobaetisca* Staniczek & Bechly, 2002 (Ephemeroptera: Baetiscidae) and *Borinquena* Traver, 1938 (Leptophlebiidae: Atalophlebiinae). In: International Perspectives in Mayfly and Stonefly Research. Proceedings of the 12th International Conference on Ephemeroptera and the 16th International Symposium on Plecoptera, Stuttgart. *Aquatic Insects* **31***, Suppl. 1*: 125–136 (2009).

22. Staniczek, A. H., Godunko, R. J. & Krzemiński, W. A new fossil mayfly species of the genus *Borinquena* Traver, 1938 (Insecta: Ephemeroptera: Leptophlebiidae: Atalophlebiinae) from Miocene Dominican amber. *Annales Zoologica* **67** (1), 113–119 (2017).

23. Solórzano Kraemer, M. M. Systematic, palaeoecology, and palaeobiogeography of the insect fauna from Mexican amber. *Palaeontographica Abteilung* *A* **282**, 1–133 (2007).

24. Riek, E. F. A re-examination of the Upper Tertiary mayflies described by Etheridge and Olliff from the Vegetable Creek Tin-field. *Records of the Australian Museum* **23** (4), 159–160 (1954).


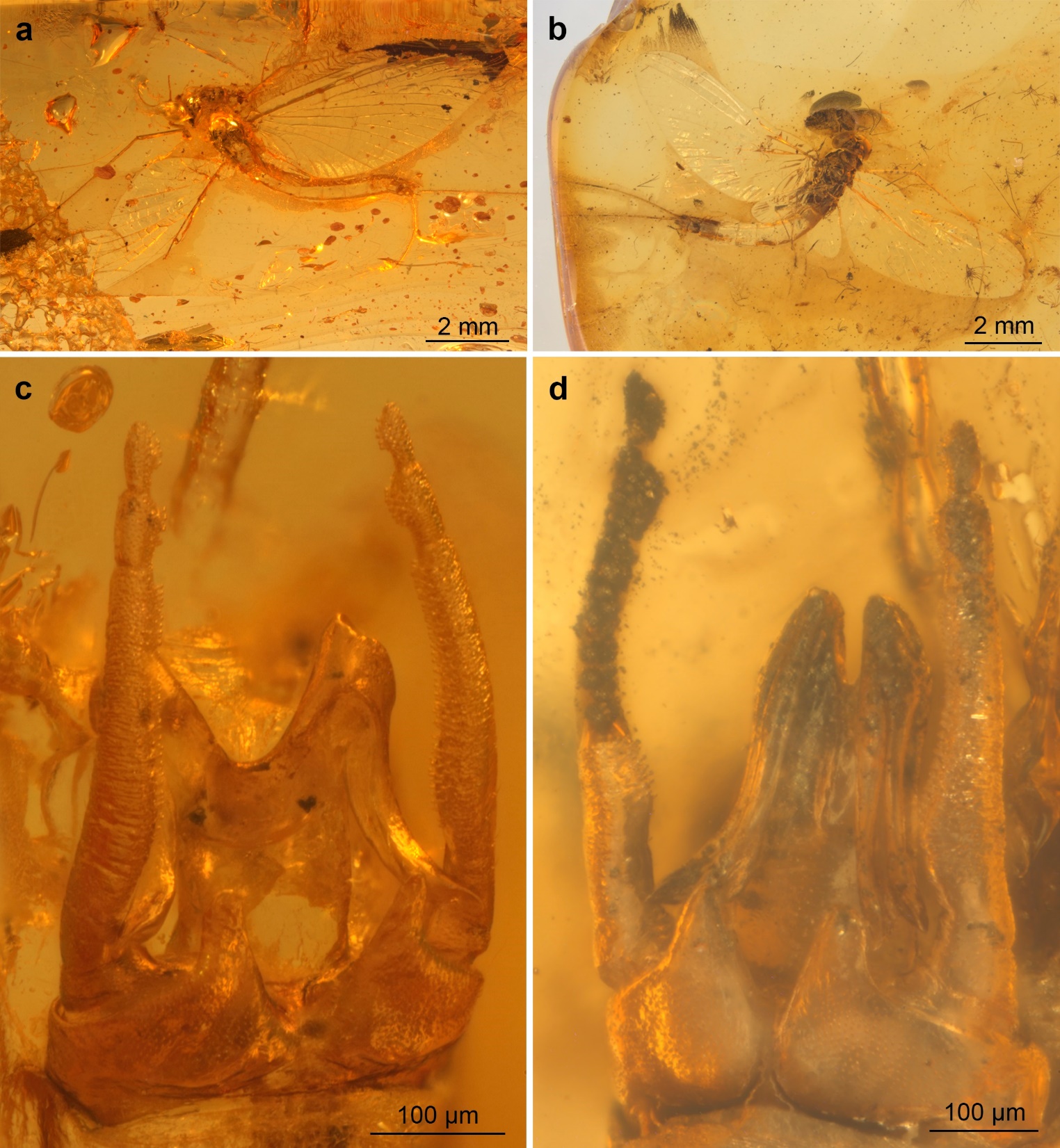


**Figure S1.** *Paraleptophlebia electra* (Kluge, 1993) (**a, c**), male imago; Eocene Baltic amber; Christel and Hans-Werner Hoffeins collection, nr. 1039: (**a**) total lateroventral view from left side; (**c**) genitalia in ventral view; *Paraleptophlebia* cf. *prisca* (Pictet & Hagen, 1856) (**b, d**), male imago, Eocene Baltic amber; Christel and Hans-Werner Hoffeins collection, nr. 363 [conspecifity of this specimen with lectotype of *P. prisca* is suggested based on morphometrics and wing venation]: (**b**) total ventral view; (**d**) genitalia in ventral view.


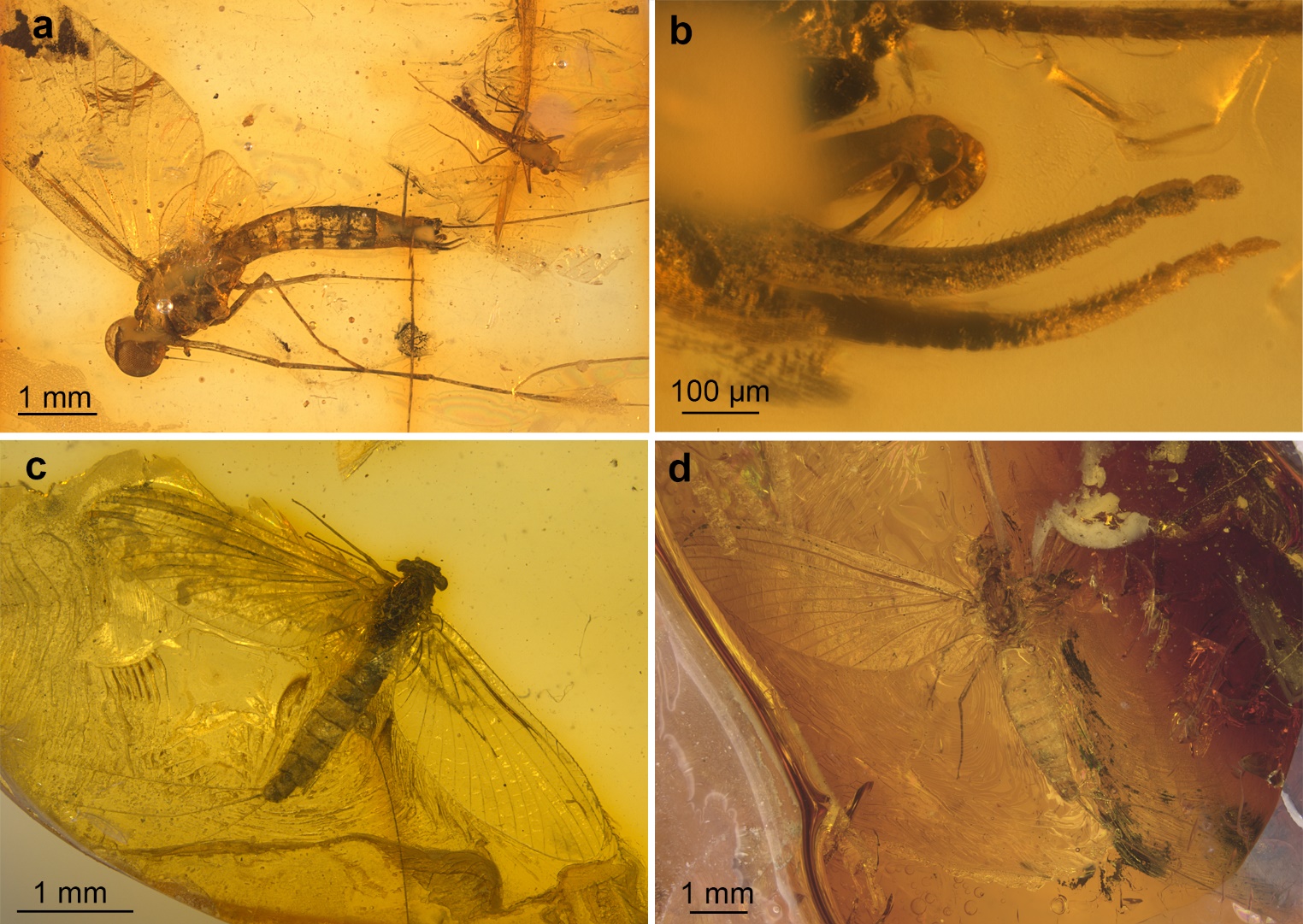


**Figure S2.** *Paraleptophlebia* sp. (**a, b**), male imago; Eocene Baltic amber; Christel and Hans Werner Hoffeins collection, nr. 1782; presumably a new undescribed species: (**a**) body of male embedded in piece of amber in left lateral view; (**b**) genitalia in left lateral view; *Paraleptophlebia* spp. (**c, d**), female imagines; Eocene Rovno amber, Schmalhausen Institute of Zoology, Kyiv collection, nr. K-3699 (**c**) and nr. K-2472 (**d**); presumably new undescribed species.


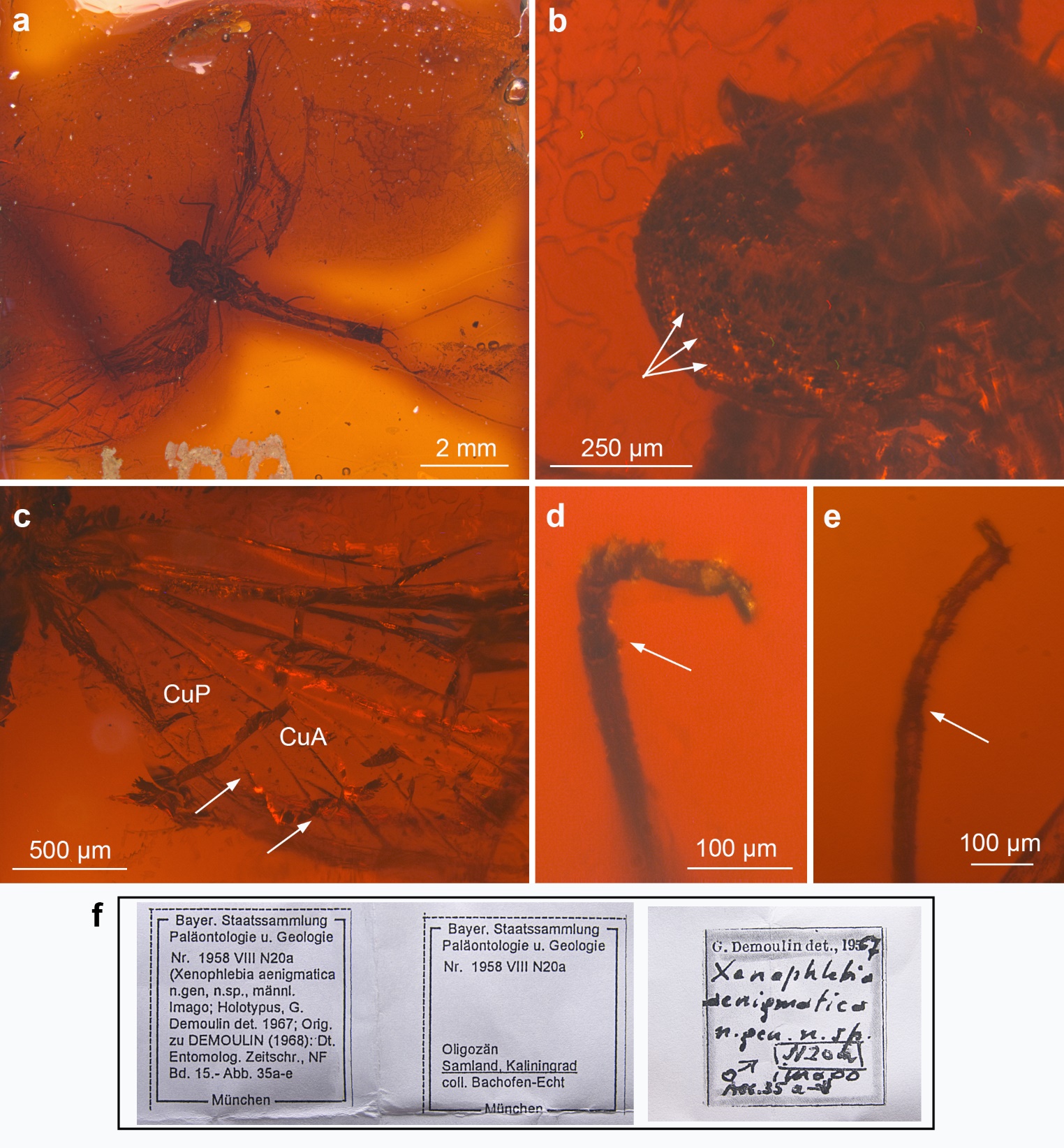


**Figure S3.** *Xenophlebia aenigmatica* Demoulin, 1968 (**a–e**), holotype, male imago; Eocene Baltic amber; Bayerische Staatssammlung für Paläontologie und Geologie, Nr. 1958 VIII N20a: (**a**) body of male embedded in piece of amber in dorsal view; (**b**) left compound eye in dorsal view [hexagonal facets are marked by arrows]; (**c**) cubital field of right forewing [two free elongated intercalaries are marked by arrows]; (**d**) tarsus of right middle leg [fusion of first tarsomere with tibia is marked by arrow]; (**e**) tarsus of right hind leg [fusion of first tarsomere with tibia is marked by arrow]; (**f**) original labels of holotype.


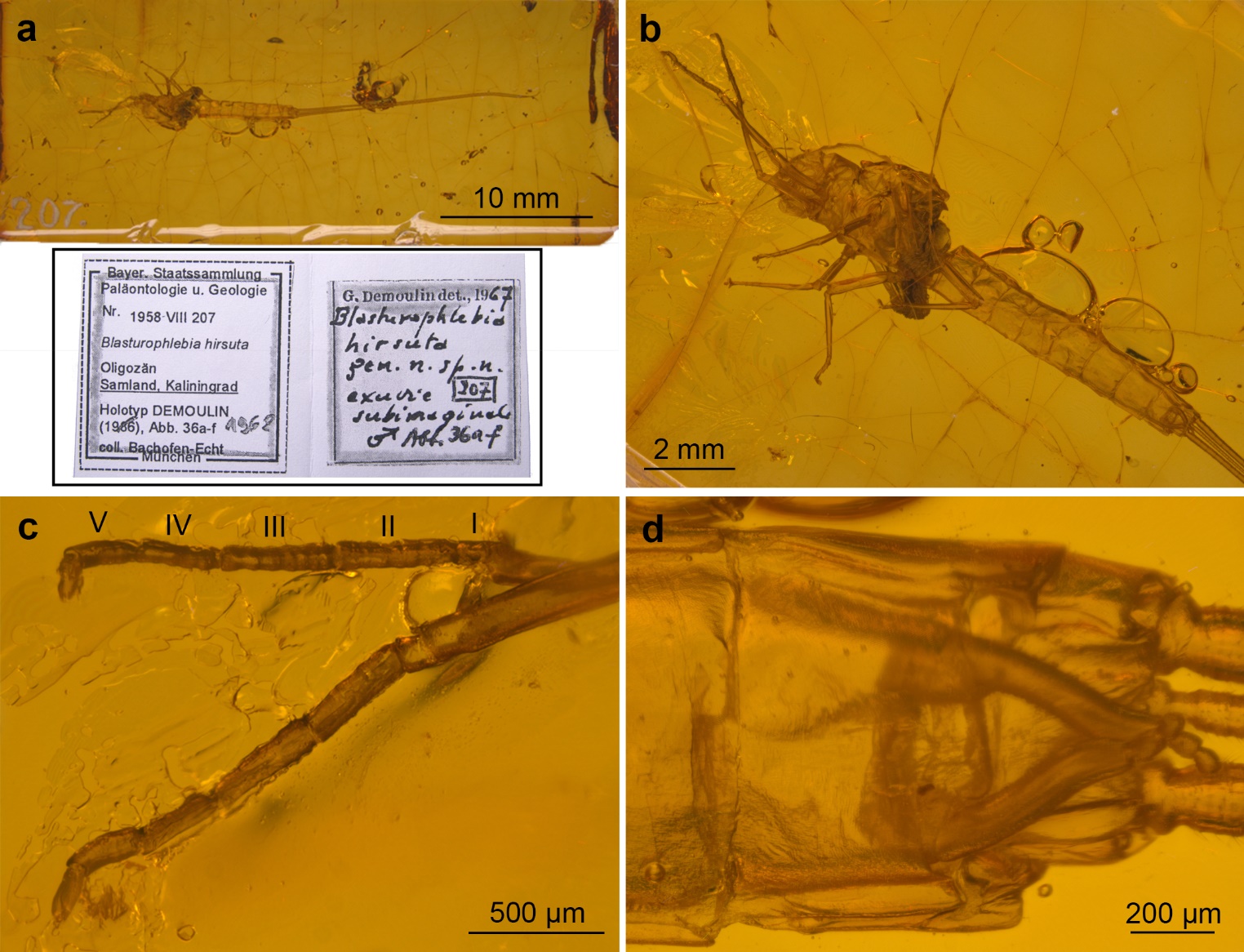


**Figure S4.** *Blasturophlebia hirsuta* Demoulin, 1968 (**a–d**), holotype, male subimaginal exuvia; Eocene Baltic amber; Bayerische Staatssammlung für Paläontologie und Geologie, Nr. 1958 VIII 207: (**a**) exuvia embedded in piece of amber in dorsal view and original labels accompanying of holotype; (**b**) exuvia in left lateral view; (**c**) forelegs [tarsomeres I–V are marked by respective Roman numbers]; (**d**) genitalia in ventral view.


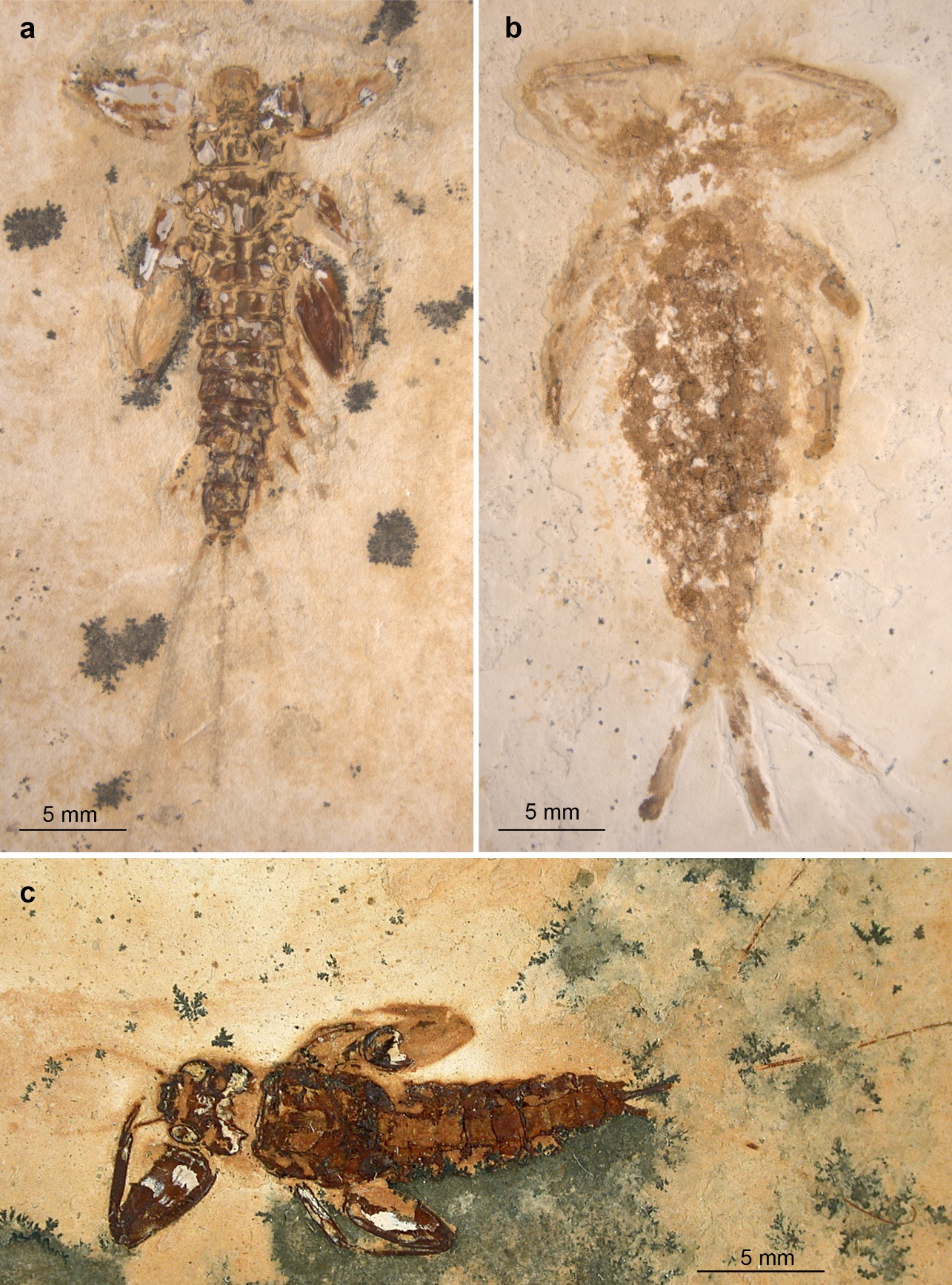


**Figure S5.** Nymphs of Leptophlebiidae from the Crato Formation (Aptian, Lower Cretaceous) of Brazil; (**a, b**) SMNS collection; (**c**) MSF collection: (**a**) nymph in ventral view, SMNS 66622; (**b**) nymph in ventral view, SMNS 66625; (**c**) nymph in dorsal view, MSF Z2.

**Table S3.** List of extinct Eocene Leptophlebiidae

| **Higher described taxon** | **Species and reference** | **Material, collection and inventory number** | **Holotype ontogenetic stage** | **Holotype provenance** | **Age** |
| --- | --- | --- | --- | --- | --- |
| *Paraleptophlebia* Lestage, 1917 | *Paraleptophlebia prisca* (Pictet & Hagen, 1856)  [orig. in *Potamanthus*; Die *im Bernstein befindlichen Neuropteren der Vorwelt*: 77, tab. VI, fig. 3] | Holotype  MNB coll. [in G.C. Berendt coll.] | Male imago | Baltic amber  Europe | Eocene  Lutetian  34–48 Ma |
| *Paraleptophlebia* Lestage, 1917 | *Paraleptophlebia electra* (Kluge, 1993) [orig. in *Leptophlebia*; *Paleontol*. *Zhurn*., 27 (1A): 47, fig. 6a] | Holotype  PIN coll.  Nr. 363/90 | Male imago | Baltic amber  Europe | Eocene  Lutetian  34–48 Ma |
| *Oligophlebia* Demoulin, 1965 | *Oligophlebia calliarcys*  Demoulin, 1965  **[***Entomologiske Meddelelser*,  34: 146, fig. 2] | Holotype  ZMUC coll.  ZMUC-900396 | Male imago | Baltic amber  Europe | Eocene  Lutetian  34–48 Ma |
| *Oligophlebia* Demoulin, 1965 | *Oligophlebia*? *longiceps*  Demoulin, 1965  **[***Entomologiske Meddelelser*,  34: 147, fig. 3A, B] | Holotype  ZMUC coll.  ZMUC-900393 | Male imago | Baltic amber  Europe | Eocene  Lutetian  34–48 Ma |
| *Calliarcys* Eaton, 1881 | *Calliarcys antiquus* **sp. nov.**  [*described here*;  Figs. 1–14, Tables 1, 2] | Holotype  SMNS coll.  SMNS BB-2515 | Male imago | Baltic amber  Europe | Eocene  Lutetian  34–48 Ma |
