# Supplementary material for: Cenozoic origins of the genus Calliarcys (Insecta, Ephemeroptera) revealed by Micro-CT, with DNA barcode gap analysis of Leptophlebiinae and Habrophlebiinae
Source: Sci Rep. 2022 Sep 8;12:15228. doi: 10.1038/s41598-022-18234-4 (PMC9458648; doi:10.1038/s41598-022-18234-4)
Supplement: Supplementary file 8 — Supplementary Information 6. [file 41598_2022_18234_MOESM8_ESM.docx]

**Supplementary video legends**

*Supplementary materials S2 and S3 [Videos (mp4)]*

*Supplementary information to*: Cenozoic origins of the genus Calliarcys (Insecta, Ephemeroptera) revealed by Micro-CT, and DNA barcode gap analysis of Leptophlebiinae and Habrophlebiinae

*Roman J. Godunko, Javier Alba-Tercedor, Michal Grabowski, Tomasz Rewicz, Arnold H. Staniczek*

**Supplementary Video S1**. Animated video of the holotype of *Calliarcys* *antiquus* **sp. nov.,** showing the scanning procedure and volume rendered images with details of the insect in different perspectives (Most the volume-rendered images were obtained with Amira software).

**Supplementary Video S2.** Animated video of the holotype of *Calliarcys* *antiquus* **sp. nov.,** showing the scanning procedure and volume rendered images with details of the insect in different perspectives (volume-rendered images were obtained with CTvox software).
